# A practical guide to unbiased quantitative morphological analyses of the gills of rainbow trout (*Oncorhynchus mykiss*) in ecotoxicological studies

**DOI:** 10.1371/journal.pone.0243462

**Published:** 2020-12-09

**Authors:** Sonja Fiedler, Hannah Wünnemann, Isabel Hofmann, Natalie Theobalt, Annette Feuchtinger, Axel Walch, Julia Schwaiger, Rüdiger Wanke, Andreas Blutke

**Affiliations:** 1 Institute of Veterinary Pathology at the Center for Clinical Veterinary Medicine, Ludwig-Maximilians-Universität München, Munich, Germany; 2 Unit 73 Aquatic Ecotoxicology, Microbial Ecology, Bavarian Environment Agency, Wielenbach, Germany; 3 Research Unit Analytical Pathology, Helmholtz Zentrum München, Neuherberg, Germany; University of Campinas, BRAZIL

## Abstract

Rainbow trout (*Oncorhynchus mykiss*) are frequently used as experimental animals in ecotoxicological studies, in which they are experimentally exposed to defined concentrations of test substances, such as heavy metals, pesticides, or pharmaceuticals. Following exposure to a broad variety of aquatic pollutants, early morphologically detectable toxic effects often manifest in alterations of the gills. Suitable methods for an accurate and unbiased quantitative characterization of the type and the extent of morphological gill alterations are therefore essential prerequisites for recognition, objective evaluation and comparison of the severity of gill lesions. The aim of the present guidelines is to provide practicable, standardized and detailed protocols for the application of unbiased quantitative stereological analyses of relevant morphological parameters of the gills of rainbow trout. These gill parameters *inter alia* include the total volume of the primary and secondary gill lamellae, the surface area of the secondary gill lamellae epithelium (*i*.*e*., the respiratory surface) and the thickness of the diffusion barrier. The featured protocols are adapted to fish of frequently used body size classes (300–2000 g). They include well-established, conventional sampling methods, probes and test systems for unbiased quantitative stereological analyses of light- and electron microscopic 2-D gill sections, as well as the application of modern 3-D light sheet fluorescence microscopy (LSFM) of optically cleared gill samples as an innovative, fast and efficient quantitative morphological analysis approach. The methods shown here provide a basis for standardized and representative state-of-the-art quantitative morphological analyses of trout gills, ensuring the unbiasedness and reproducibility, as well as the intra- and inter-study comparability of analyses results. Their broad implementation will therefore significantly contribute to the reliable identification of no observed effect concentration (NOEC) limits in ecotoxicological studies and, moreover, to limit the number of experimental animals by reduction of unnecessary repetition of experiments.

## Introduction

In ecotoxicological studies, the rainbow trout (*O*. *mykiss*) is frequently used as a sensitive experimental fish species to examine the effects of various surface water pollutants, including diverse chemicals, pharmaceuticals, heavy metals, as well as solid particles such as microplastic, on aquatic organisms [1–5]. In a typical experimental approach, different groups of fish are exposed to various concentrations of a test substance under defined experimental conditions [6]. The patterns and severities of observed (histo-) morphological organ/tissue alterations, combined with hematological analysis findings and clinical-chemical test results, are then used to define *inter alia* the no observed effect concentration (NOEC) of the examined test substance [4,6,7]. These findings often have far-reaching consequences, as they are included in risk assessment of test substances and regularly provide the basis for specification of the legal concentration limits of the substance in surface waters (predicted no effect concentration (PNEC)) [8–11]. Therefore, the comparability and reproducibility of analyses results of different ecotoxicological studies examining rainbow trout for detection of NOEC of a specific test substance are particularly important. However, there are some examples of aquatic toxicology studies examining the effects of exposure of rainbow trout to the same substance, in which the NOEC differs significantly over multiple orders of magnitude [4,7,12,13]. Histopathological diagnoses and, in particular, qualitative gradings of the severities of detected lesions often exhibit a substantial variability between different observers and different studies. This is especially relevant for the evaluation of histopathological alterations in experimental animals exposed to low concentrations of test substances, where lesions might be subtle and not manifested in all individuals, respectively in all examined samples of one animal [7,8,14]. Following exposure to a broad variety of different aquatic pollutants, early detectable morphological alterations in fish often tend to manifest in the gills due to their delicate histomorphology and continuous exposure to the ambient water [15–17]. Next to respiration, fish gills are also the primary site for osmoregulation, excretion of nitrogenous waste products and metabolism of hormones and xenobiotics [18]. Thus, histopathological gill lesions can serve as sensitive indicators of toxic effects of low exposure concentrations of aquatic pollutants [4,13,16,19]. Besides easily recognizable qualitative histomorphological gill lesions, such as fusion of adjacent secondary gill lamellae, thickening of filament tips, inflammatory cell infiltrations, focal cell proliferations and erosive/ulcerative lesions [3], relevant alterations also affect different quantitative morphological gill properties, which cannot be adequately assessed by microscopic examination alone [20–23].

Relevant quantitative parameters characterizing gill morphology *e*.*g*., include the total volume of the secondary gill lamellae, the total surface area of the respiratory epithelium of the gills and the thickness of the diffusion barrier (*i*.*e*., the distance between the epithelial cell surface and the capillary space in the secondary lamellae). In a given study, also any other quantitative morphological parameter might be of interest to characterize distinct histomorphological or ultrastructural gill alterations, including, but not limited to *e*.*g*., the total volume of an inflammatory infiltrate present in the gills, the total number and the mean cellular volume of a specific cell type, or the volumes of distinct cell organelles in a particular cell type. Due to the complex 3-D tissue-architecture of gills, these quantitative morphological parameters cannot adequately be determined in standard histological sections taken from a few deliberately chosen gill locations. Accurate, *i*.*e*., precise and unbiased estimates of quantitative morphological gill parameters can be obtained using so-called “unbiased quantitative stereological analysis” methods and techniques, warranting for a reproducible and objective quantitative characterization of relevant organ alterations [8,14,24–26]. Using appropriate sampling designs, probes and test systems, quantitative stereological analyses examine two-dimensional (2-D) histological sections to provide estimates of three-dimensional (3-D) morphological parameters (*i*.*e*., volumes, surface areas, lengths, and numbers) of the examined tissue structures of interest with statistically defined error probabilities [24,25]. During the last five decades, quantitative stereological analysis techniques have been continuously refined and have become the generally accepted “gold standard” for objective quantification of morphological tissue properties in diverse life science disciplines [25,27,28]. By now, several scientific societies and high-impact journals have released editorial policies, demanding stereological analysis techniques for studies reporting quantitative morphological data of biological samples [27,29–32].

In the 70´s, 80´s and 90´s of the past century, several early, basic research studies examined morphological gill parameters, such as the gill respiratory area or the oxygen diffusion barrier in diverse fish species, using simple morphometric analysis tools [20,33–35]. However, modern unbiased quantitative stereological analysis approaches have rarely been implemented to characterize morphological parameters of fish gills, including determination of the volume of interbranchial lymphoid tissue in Atlantic salmon (*Salmo salar*) [36], volume- and numerical volume densities of the structural gill filament components of the Nile tilapia (*Oreochromis niloticus*) [21], gill volume, surface and water-blood barrier thickness of the gills of South American lungfish (*Lepidosiren paradoxa*) [37], the Brasilian pirarucu (*Arapaima gigas*) [38,39], or the striped catfish (*Pangasianodon hypophthalamus*) [40,41].

Due to considerable differences in the size of studied fish species and correspondingly the size of their gills, several of the previously described quantitative stereological analysis methods cannot practically be applied for examination of the gills of rainbow trout with body sizes of 300–2000 g, commonly used in ecotoxicological studies [42–46].

In the past decade, several “deep tissue imaging” methods based on examination of optically cleared (*i*.*e*., transparent) samples by laser light sheet fluorescence microscopy (LSFM) have been developed, allowing microscopic examination in 3-D without the necessity of preparation of 2-D histological sections [47–53]. Besides visualization of complex 3-D architectural tissue properties, such as *e*.*g*., vascularization patterns [54,55], LSFM of optically cleared samples also provides an elegant, fast and effective approach for the quantification of diverse histomorphological parameters, thus holding a great potential for quantitative characterization of the morphology of gill samples [53,56–58]. However, qualitative or quantitative examinations of optically cleared gills by LSFM have not been reported so far.

Aim of the present article is to provide a comprehensive collection of practicable methods for unbiased quantification of relevant morphological parameters of rainbow trout gills, featuring both “classical” unbiased quantitative stereological sampling and analysis methods based on examination of light- and electron microscopic sections, as well as protocols for LSFM-based quantitative morphological analyses of optically cleared gills. The featured methods and protocols shown here provide a basis for standardized and representative state-of-the-art quantitative morphological analyses of trout gills in ecotoxicological studies, ensuring the unbiasedness and reproducibility as well as the inter- and intra-study comparability of analysis results. This will significantly contribute to reliably identify NOEC limits of aquatic pollutants and help to reduce the number of sacrificed fish in ecotoxicological studies by avoiding unnecessary repetitions of experiments.

## Experimental fish, ethical statement

For development and demonstration of the methods shown in the present study, rainbow trout (*O*. *mykiss*) (n = 5) of both sexes and body weights ranging from 300 to 2000 g were sacrificed. For comparison of the analysis results of different quantitative morphological analysis methods, gill samples of four different fish were used. The fish were obtained from the breeding facility of the Bavarian Environment Agency in Wielenbach, Germany. Fish were euthanized with tricaine methanesulphonate solution (500 mg/l, Tricaine Pharmaq^®^ 1000 mg/g (Pharmaq Ltd., United Kingdom)) and subsequent mechanical disruption of the brain after circulatory arrest, using a sharp 14 gauge cannula (Braun^®^ Sterican^®^, B.Braun Melsungen AG, Germany). The use of the fish in this study was approved by the institutional review board of the Institute for Veterinary Pathology at the Center for Clinical Veterinary Medicine of the Ludwig-Maximilians University Munich and performed in accordance with the relevant guidelines and regulations and with permission of the local authorities.

## Contents

**Section 1** provides an initial, short introduction to the basic principles and methodological aspects of unbiased quantitative stereological analyses. Essential aspects of trout gill morphology, histology and ultrastructure are recapitulated in **Section 2**. **Section 3** outlines the relevant quantitative morphological gill parameters. **Sections 4–7** illustrate the work steps from gill-preserving killing to gill dissection and adequate processing for further analyses. **Sections 8–15** sequentially guide through the sampling- and analysis procedures for quantitative stereological analysis of relevant morphological gill parameters. The application of LSFM of optically cleared gill samples in quantitative histomorphological analyses is described in **Section 16**.

**Table:** 

Topic	**Section**
Basic principles of unbiased quantitative stereological analyses	1
Trout gill morphology and nomenclature	2
Relevant quantitative stereological gill parameters	3
General experimental design for quantitative stereological analyses of trout gill morphology in ecotoxicological studies	4
Adequate killing methods for quantitative stereological gill analyses	5
Vascular perfusion fixation of the gills	6
Excision and fixation of the gills	7
Determination of the gill filament volume	8
Systematic uniform random (SUR) sampling of representative gill filament samples	9
Randomization of the orientation of the sample section plane	10
Determination of plastic embedding-related three-dimensional shrinkage of the gill filaments	11
Estimation of volume densities and total volumes of distinct gill filament structures	12
Estimation of the surface area of the secondary lamellae in the gill filaments	13
Estimation of the total number, the total volume and the mean volume of epithelial cells in the secondary gill lamellae	14
Determination of the true harmonic mean of the diffusion barrier thickness in the secondary gill lamellae	15
Laser light sheet fluorescence microscopy (LSFM) of optically cleared samples and its application for quantitative morphological analyses of trout gills	16.1
LSFM-based determination of volume- and surface area densities of secondary gill lamellae in the gill filaments	16.2

### 1. Basic principles of unbiased quantitative stereological analyses

**Fig 1:**
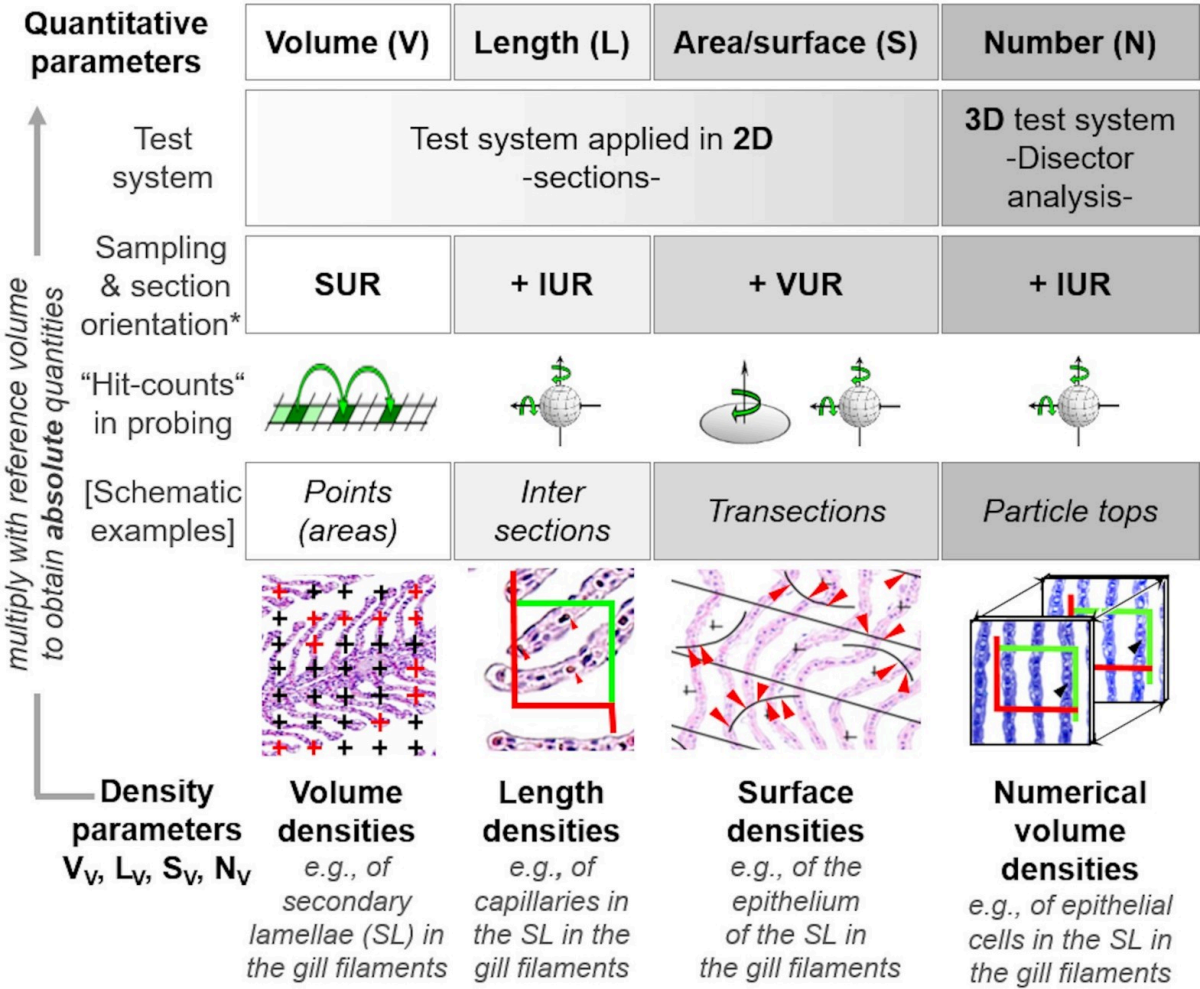
Quantitative parameters, stereological test systems, sampling, section orientation and stereological probes in quantitative stereological analyses. Volume densities (V_V(structure of interest/reference space)_), length densities (L_V(structure of interest/reference space)_) and surface area densities (S_V(structure of interest/reference space)_) are estimated in representative, systematically uniform random (SUR) sampled 2-D sections of the reference space. Volume densities are deduced from the fractional areas of the structure of interest and the reference space, determined *e*.*g*., by point counting. Length densities are estimated on isotropic uniform random (IUR) sections from the number of intersections of the structure of interest with the section area. (*Note that the present guidelines do not cover quantitative stereological estimation of length parameters of gill structures*. *Determination of the true harmonic mean of the diffusion barrier thickness in the secondary lamellae (SL) is described in* ***Section 15***.) For estimation of surface area densities, the number of interactions of the examined surface area with appropriate stereological probes is counted in vertical uniform random (VUR) sections. Estimation of numerical volume densities (N_V(structure of interest/reference space)_) requires 3-D test systems, such as the physical disector, to sample and count particles. A physical disector is a stereological probe used for unbiased counting and sampling of particles. It consists of two parallel histological sections (a reference section and a look-up section) with a defined distance, thus defining a known tissue volume. Particles that are sectioned in the reference section, but not in the look-up section are counted (Q^-^), using the unbiased counting frame. Estimation of N_V(structure of interest/reference space)_ using the physical disector is described in detail in **Section 14**. Absolute quantities of volumes, lengths, surfaces and numbers are obtained from the respective densities and the total reference space volume. Mean particle volumes are calculated from their volume densities and their numerical volume densities in the reference space, as described in **Section 14**. *The section plane orientation illustrated for the corresponding parameters is highly recommended, but there are several options for most morphological parameters regarding the orientation of the section plane.

Commonly applied quantitative stereological analysis approaches follow few fundamental principles, which are briefly outlined below and schematically illustrated in **Fig 1**. Quantifiable morphological tissue parameters generally comprise volumes, surface areas, lengths and numbers of tissue structures within the organ, tissue, or organ compartment harboring these structures (*i*.*e*., the reference compartment). Quantification of these 3-D morphological parameters is achieved by analysis of representative (2-D) histological sections of the reference compartment [25,26].

Systematic uniform random sampling (SURS) methods are used to generate a sufficient number of representative samples from the entire reference compartment, adequately reflecting the quantitative morphological parameters of interest in the examined organ/tissue. To warrant unbiased analysis results, appropriate random sampling procedures are applied on all hierarchical sampling levels, *i*.*e*., sampling of the histological sections cut from the blocks of embedded specimens as well as sampling of the test fields to be examined within these sections [26,59]. The dimensional reduction that is associated with the examination of 2-D sections of 3-D tissue structures, is inevitably associated with a loss of structural information: in 2-D sections, 3-D volumes will be represented as areas, surfaces present as transection lines and lengths as intersection points with the section plane, whereas the number of particles in a 3-D reference volume has no direct equivalent in a 2-D section [14,25,60]. Moreover, the areas, shapes and number of (anisotropic) 3-D particles in 2-D sections, as well as the lengths of transections of surfaces or the number of intersections of lengths within the plane of a histological section generally depend on the orientation of the section plane relative to the sectioned sample, as well as on the sizes, shapes and the spatial distributions of the sectioned structures within the reference compartment [8,25,26,60]. Finally, quantitative stereological determination of surface areas, lengths and particle numbers is affected by the tissue shrinkage that occurs during the histological embedding process of the specimen [26,60,61].

In quantitative stereological analyses these issues are addressed by analysis of 2-D histological sections with randomly oriented section planes (isotropic uniform random (IUR) or vertical uniform random (VUR) sections) [14,24,62], by using suitable histological (plastic-) embedding media allowing for estimation and correction of embedding-related tissue shrinkage, using the linear tissue shrinkage correction factor f_s_ [26,60,63,64], and by appropriate stereological probes and test systems for analysis of the sections [24,65]. **Table 1** provides a brief summary of the appropriate sample section plane orientations, embedding media and adequate tissue shrinkage correction factors for the relevant quantitative morphological parameters.

**Table 1:** Adequate sample section plane orientation, embedding medium and tissue shrinkage correction factor (f_s_) for different quantitative morphological parameters.

Parameter	Section plane orientation	Embedding medium	f_s_
**Volume density (V**_**V(X/Y)**_**)**^**a**^	Arbitrary, VUR, IUR	Paraffin, plastic resin	-
**Surface area density (S**_**V(X/Y)**_**)**^**b**^	VUR, IUR	Plastic resin^e^	f_s_
**Length density (L**_**V(X/Y)**_**)**^**c**^	IUR	Plastic resin^e^	f_s_^2^
**Numerical volume density (N**_**V(X/Y)**_**)**^**d**^	Arbitrary, VUR, IUR	Plastic resin^e^	f_s_^3^

^**a**^V_V(X/Y)_, the volume density of a tissue compartment or cell type within the reference compartment can be determined using arbitrary-, VUR- or IUR sections of SUR sampled specimen. As a dimensionless parameter, volume densities are generally independent of the effect of (homogenous, *i*.*e*., an overall equal extent of embedding-related tissue shrinkage of different histological gill structures) embedding-related tissue shrinkage, so embedding in plastic resin medium or paraffin wax is appropriate and no correction for embedding-related tissue shrinkage is performed. **X**: Structure of interest, **Y**: Reference compartment.

^**b**^Estimation of S_V(X/Y)_ is principally feasible in VUR- or IUR sections of plastic resin-embedded samples. As a shrinkage-sensitive parameter, S_V(X/Y)_ needs to be corrected for embedding-related tissue shrinkage, *i*.*e*., it needs to be multiplied by f_s_.

^**c**^Estimation of L_V(X/Y)_ has to be performed on IUR sections of plastic resin-embedded samples. As a shrinkage-sensitive parameter, L_V(X/Y)_ needs to be multiplied by f_s_^2^ for correction of embedding-related tissue shrinkage.

^**d**^Estimation of N_V(X/Y)_ is feasible in arbitrary-, VUR- or IUR sections of plastic resin-embedded specimen, N_V(X/Y)_ as a shrinkage-sensitive parameter needs to be multiplied by f_s_^3^ for embedding-related tissue shrinkage correction. ^e^Estimation of S_V(X/Y)_, L_V(X/Y)_ and N_V(X/Y)_ is also possible using paraffin as embedding medium [66], however it is not recommended due to the paraffin embedding-related pronounced tissue deformation. *For details, the interested reader is referred to several excellent publications [14,24–26,62,67,68]*.

In the outlined experimental approach (**Section 4**), the analysis of histological sections yields relative quantities of the examined morphological parameter per volume unit of the reference compartment (*i*.*e*., volume-, surface area-, length densities and numerical volume densities). Interpretation of these relative parameters alone, however, may be inconclusive, since changed densities may result from changes of the target structures, as well as from altered volumes of the reference compartments, or both, which is referred to as the “reference trap” [25,67]. The eventually relevant data, *i*.*e*., the absolute quantities of volumes, surface areas, lengths or numbers of the examined tissue structures of interest, are calculated by multiplication of the respective density parameter by the volume of the complete reference compartment [24,67]. Therefore, determination of the reference compartment’s volume must not be omitted [69,70]. Since determination of different quantitative morphological parameters may require different sampling designs, special sample processing procedures and histotechniques, as well as application of distinct stereological test systems, an adequate planning of the sampling strategy in advance is essential in any quantitative stereological study [70]. For a more detailed discussion of the general principles of quantitative stereology, the interested reader is referred to the standard textbooks of stereology and several excellent reviews of quantitative stereological analyses in biomedical research [25,26,32,71].

### 2. Trout gill morphology and nomenclature

**Fig 2:**
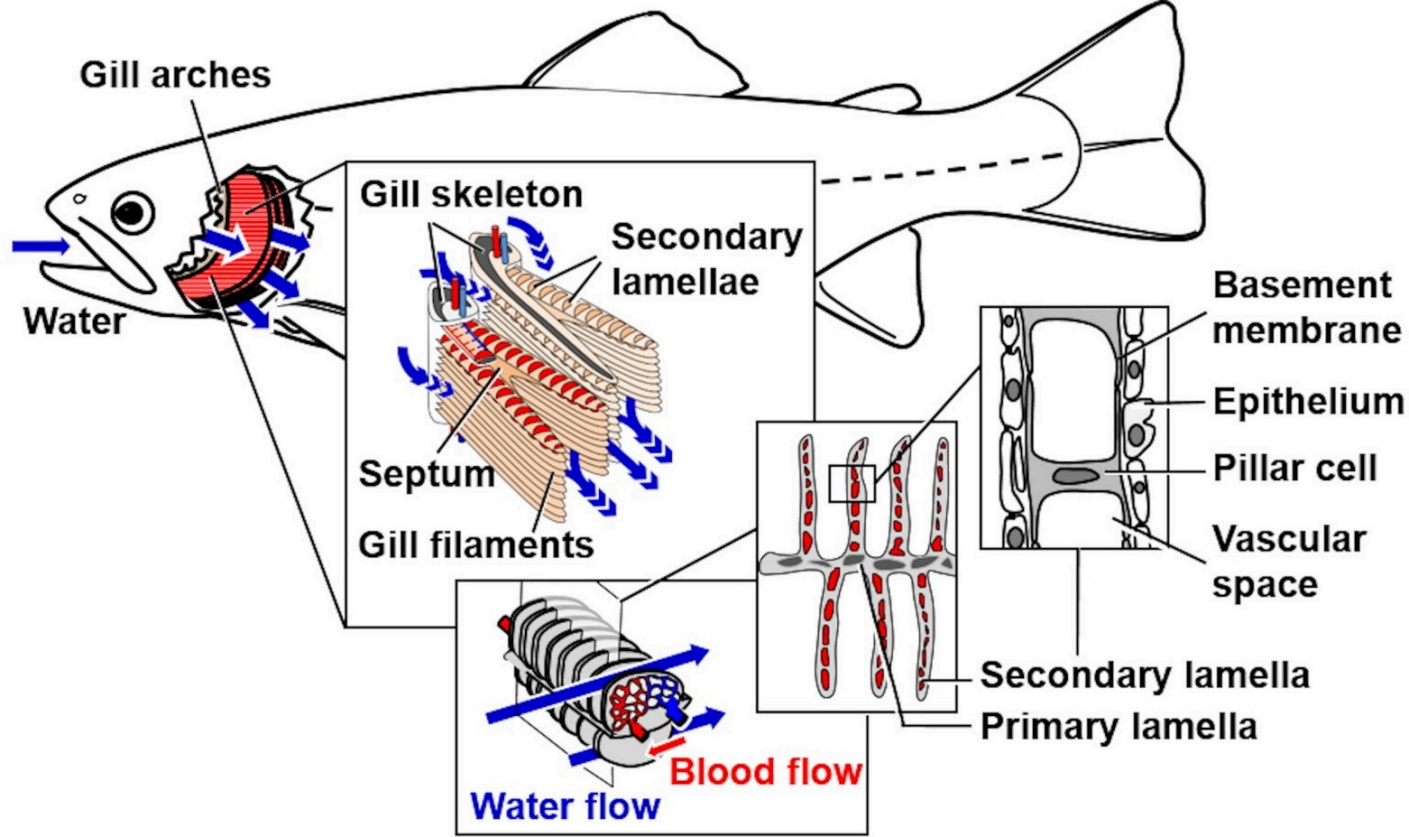
Schematic illustration of the 3-D histo-architecture of the gills and corresponding 2-D histological sections.

**Fig 3:**
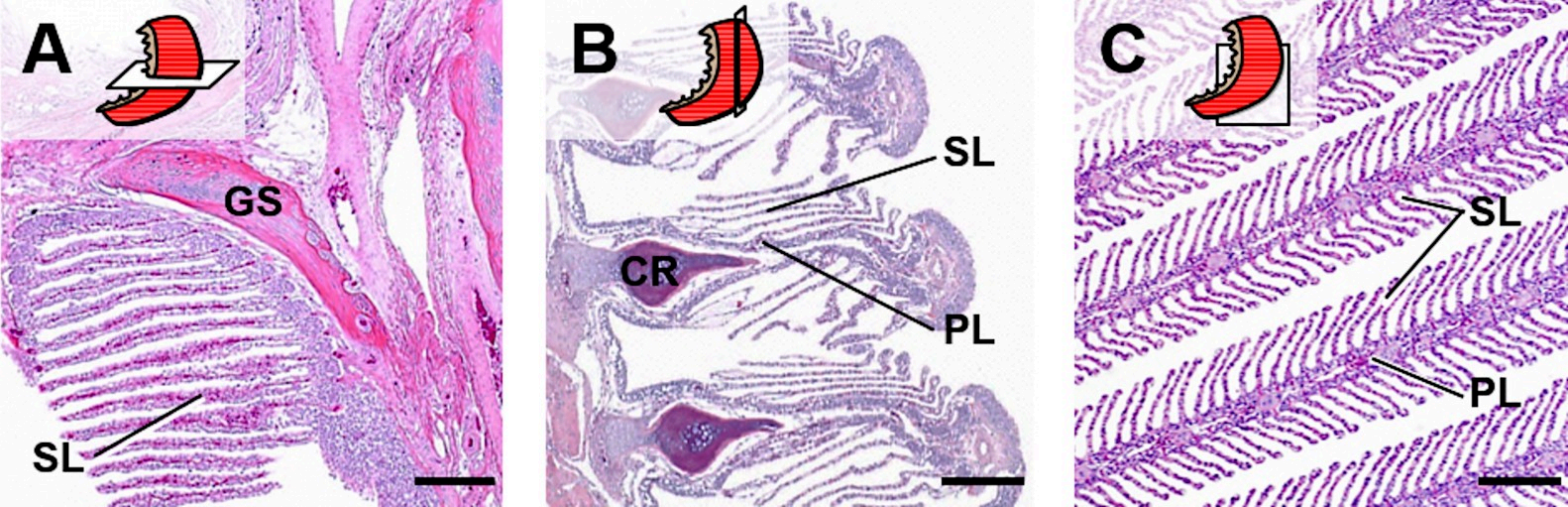
Appearance of histological gill section profiles in different section plane orientations (schematically indicated). **A.** Transverse section. **B.** Frontal section. **C.** Sagittal section. Important morphological structures are indicated: **GS**: Gill arch support skeleton, **CR**: Cartilage rod, **PL**: Primary gill lamellae, **SL**: Secondary gill lamellae. Haematoxylin and Eosin (HE)-stained sections of formalin-fixed and paraffin-embedded (FFPE) gills. Bars = 200 μm.

**Fig 4:**
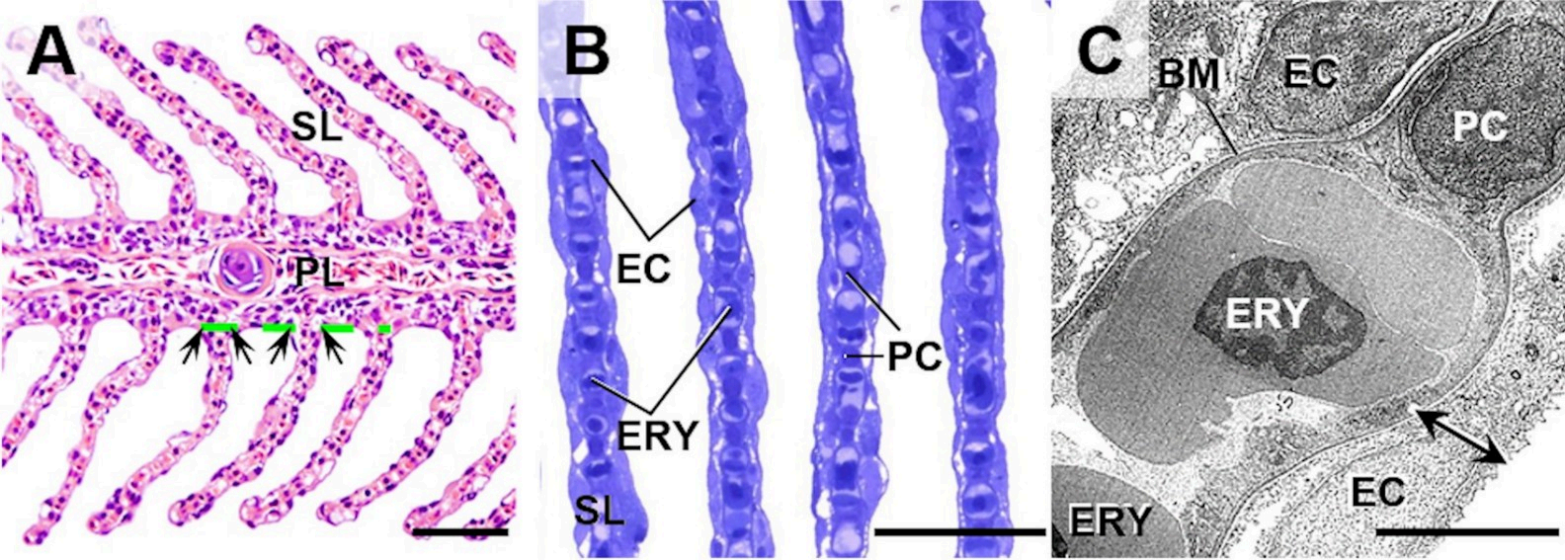
Gill histomorphology and ultrastructure. Important morphological structures are indicated: **PL**: Primary gill lamellae, **SL**: Secondary gill lamellae, **PC**: Pillar cell, **EC**: Epithelial cell, **ERY**: Nucleated erythrocyte inside a SL-capillary, **BM**: Basement membrane of the SL-capillary. **A, B.** Light-microscopic histomorphology of gill filaments (sagittal section). **A.** The anatomical border between the SL and the PL is indicated by arrows and a dashed green line. FFPE. HE. **B.** Semithin section of Epon-embedded gill filaments. Toluidine blue (TB) staining. Bars = 50 μm. **C.** Ultrastructure of the secondary gill lamella. The oxygen diffusion barrier between the epithelial surface of the SL and the capillary lumen is indicated by a double arrow. Transmission electron micrograph. Bar = 5 μm.

Analyses of qualitative and quantitative histomorphological gill alterations require consideration of the physiological functions and the complex 3-D gill architecture, which are briefly recapitulated here and illustrated in **Figs 2–4** and **S1**. As a teleost freshwater fish species, the rainbow trout possesses four pairs of gills (*holobranchs*) (I-IV, from rostral to caudal, the pseudobranch is not taken into account here) [18,72]. Each holobranch is composed of a bony gill arch, bearing gill rakers on its rostral concave margin and hemibranchs (*i*.*e*., two rows of gill filaments) on its caudal convex margin (**Figs 2** and **S1**). In trout, the hemibranchs are supported by an interbranchial septum that extends from the basis of the filaments up to 60% of the gill filament length and contains lymphoid tissue [72,73]. Each gill filament (*primary lamella*) is supported by a cartilaginous rod and bears numerous, parallel oriented, delicate respiratory lamellae (*secondary lamella*) originating from the dorsal and ventral side of the primary lamella. Representing the functional unit of the gills, the secondary lamella is the site of gas and ion exchange as well as metabolism of diverse endogenous and exogenous substances [18]. In histological gill sections, we define the border between the secondary and the primary lamellae by an imaginary line tangential to the epithelial surface of the primary lamella between two adjacent secondary lamellae at the transition of the multilayered primary lamella epithelium into the thinner epithelium covering the secondary lamellae [74] (**Fig 4A**). The respiratory surface area is increased by forming plate-like secondary lamellae which are composed of complex vascular networks defined by pillar cells and an epithelium composed of different specialized cell types, mainly pavement-, but also *e*.*g*., chloride- or goblet cells [74]. The gas exchange barrier (**Figs 2** and **4C**) between the water and the vascular spaces consists of the secondary lamellar gill epithelium, its basement membrane and the pillar cell flanges delimiting the lamellar blood spaces, endothelial cells are only partially present as lining of the marginal channel [33,72]. The perfusion of the secondary lamellae is regulated by contraction of the pillar cells and varies in response to *e*.*g*., stress, hypoxia or increased activity [75]. As indicated in **Figs 2** and **S1**, the water flows from the buccal chamber into the opercular chamber, passing between the secondary lamellae. The flow rates of water perfusing the gills at a time differ between fish species as well as between individual gills and different parts of one gill, and can be regulated (*e*.*g*., by opercular movement) depending on the current oxygen demand of the fish. Within the vascular network of the secondary lamellae, the direction of blood flow is opposed to the direction of the water flow. Due to this counter-current principle, the large surface of the secondary lamellae, the short oxygen diffusion distance as well as the adaptive regulation of water perfusion through different gill regions, the gas exchange at the secondary lamellar epithelium is highly efficient [18,72,76].

Due to the highly anisotropic 3-D architecture of the gills (*i*.*e*., the strictly directed spatial orientations and positional relations of different structural elements), the presentation of different gill structures in histological (2-D) sections strongly depends on the position and the 3-D orientation of the section plane relative to the sectioned gill specimen, as illustrated in **Fig 3**.

### 3. Relevant quantitative stereological gill parameters

The present guidelines feature approaches for the determination of selected quantitative stereological parameters (listed in **Table 2**), which are highly relevant for the detection and characterization of (trout) gill lesions in ecotoxicological studies.

**Table 2:** Relevant quantitative stereological gill parameters.

Parameters	Abbreviation
Total gill filament (GF) volume	**V**_**(GF)**_
Volume density of secondary lamellae (SL) in the GF	**V**_**V(SL/GF)**_
Total volume of SL in the GF	**V**_**(SL,GF)**_
Surface area density of the SL in the GF	**S**_**V(SL/GF)**_
Total surface area of SL in the GF	**S**_**(SL,GF)**_
Volume density of epithelial cells (EC) in the SL	**V**_**V(EC/SL)**_
Total volume of EC in the SL	**V**_**(EC,SL)**_
Numerical volume density of the EC in the SL	**N**_**V(EC/SL)**_
Total number of EC in the SL	**N**_**(EC,SL)**_
Mean cellular volume of EC in the SL	${\bar{v}}_{\left(\mathrm{EC},\mathrm{SL}\right)}$
True harmonic mean of the diffusion barrier (DB) thickness of the SL	**T**_**h(DB)**_

These morphological parameters are directly related to physiologically relevant gill functions such as the capacity of gas or ion exchange (*i*.*e*., the respiratory surface or the thickness of the diffusion barrier), or to common pathophysiological reaction patterns of gills exposed to different noxious agents (*e*.*g*., proliferation or loss of cells in the gill epithelium, characterized by the total number of cells). Previous studies have shown that the exposure to aquatic pollutants and diverse chemical compounds frequently manifests in a large variety of histopathological gill alterations and associated changes in the featured quantitative morphological gill parameters [3,16,33,76,77]. They can therefore be used as sensitive indicators of toxic effects of aquatic xenobiotics in ecotoxicological studies, as well as for the objective quantification and comparison of the extent of defined gill lesions in different (ecotoxicological and non-ecotoxicological) experimental settings or the quantification of morphological gill parameters in non-pathological experiments. Using the analysis protocols described below, values for V_(GF)_, V_V(SL/GF)_, V_(SL,GF)_, S_V(SL/GF),_ S_(SL,GF)_, V_V(EC/SL)_ and V_(EC,SL)_ were determined in the gills of rainbow trout of ~1300 g to attest the feasibility of the featured “classical” quantitative stereological methods and to evaluate the results of the methods with quantitative morphological analysis results based on laser light sheet fluorescence microscopic (LSFM) examination of gill filament samples (**Section 16**).

### 4. General experimental design for quantitative stereological analyses of trout gill morphology in ecotoxicological studies

**Fig 5:**
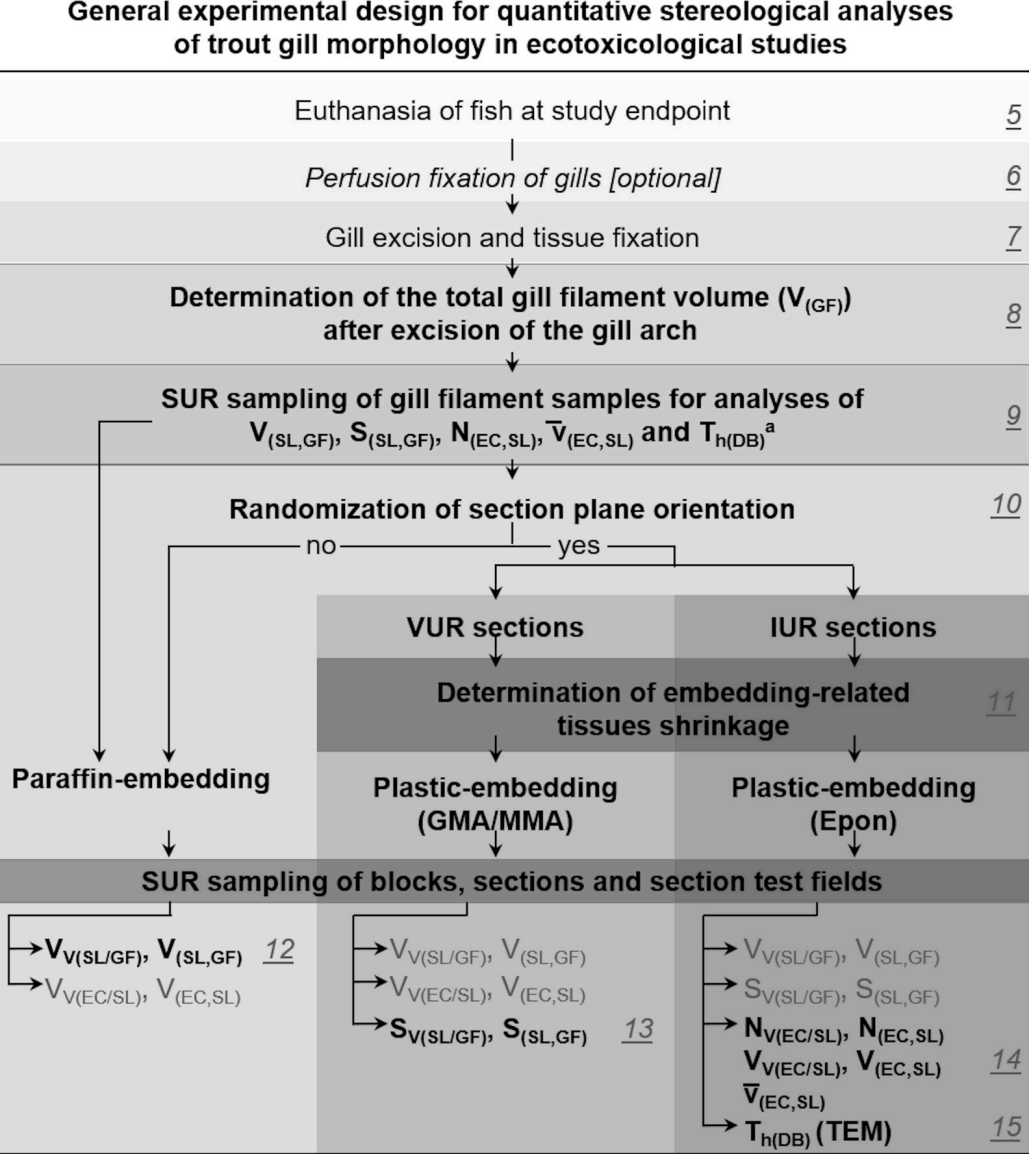
General experimental design for quantitative stereological analyses of trout gill morphology in ecotoxicological studies. The paper sections containing the respective sample processing and analysis steps are indicated (5–15). At the end point of the study, fish are euthanized, using a gill-preserving killing method (**Section 5**). Optional perfusion fixation, excision-, fixation- and volumetry of gills after removal of the gill arch are described in **Sections 6–8**. **Section 9** presents the systematic uniform random (SUR) sampling of representative gill filament specimens for stereological analyses. ^a^After SUR sampling of the specimens, it is strongly recommended to store the remaining fixed gills and not discard them until final completion of the study. The subsequent processing steps and analysis methods depend on the individual quantitative stereological parameters of interest (**Sections 10–15**).

To provide accurate estimates of quantitative morphological tissue parameters, any quantitative stereological study essentially depends on an appropriate experimental sampling design. The sampling design has to consider the determination of the volume of the reference compartment of interest (*e*.*g*., the total gill filament volume), the application of efficient approaches for random sampling of organ/tissue locations, histological sections and section test fields, as well as appropriate methods for the randomization of the 3-D orientation of the section planes and for consideration of tissue shrinkage related to the histological embedding process [70]. Depending on the analysis parameter(s) of interest (**Tables 1** and **2**), different stereological test systems and probes (**Fig 1**) are required and, correspondingly, also different histotechniques and sample processing steps must be applied (paraffin- or plastic-embedding media, light- (LM) or electron microscopy (EM)). Since inappropriately processed samples can generally not be used for retrospective analyses of quantitative stereological parameters, the experimental sampling and analysis design of a stereological study must consider all these eventualities in advance. A detailed schematic overview of an experimental study design, covering all the relevant quantitative morphological gill parameters listed in **Table 2**, is provided in **Fig 5**. This design can be individually adapted to the requirements of a given study. From **Fig 5** it is evident that volumetry and SUR sampling of the gill filaments is mandatory, whereas randomization of the section plane orientation and tissue shrinkage correction, the choice of the appropriate histological embedding medium and the subsequent stereological analysis procedures depend on the individual morphological parameter(s). For estimation of quantitative morphological parameters that are shrinkage-sensitive and/or require generation of thin sections with verifiable thicknesses (S_(SL,GF),_ N_(EC,SL)_, ${\bar{v}}_{\left(\mathrm{EC},\mathrm{SL}\right)}$, T_h(DB)_), samples are embedded in plastic resin-based embedding media, such as glycol methacrylate/methyl methacrylate (*i*.*e*., GMA/MMA) [78] or epoxy resin (Epon, *aka* Glycid ether 100; Serva, USA). Compared to paraffin, embedding in plastic resins causes less and more homogenous 3-D tissue shrinkage and allows for sectioning of thinner sections with consistent thicknesses [14,64,79,80]. Plastic-embedding media are therefore often preferred in quantitative stereological studies [14]. In contrast, estimation of volume density parameters (*e*.*g*., V_V(SL/GF)_) is largely independent of embedding-related (homogenous) tissue shrinkage and can therefore be performed using paraffin sections [25,26]. Estimation of volume- and numerical volume density in SUR sampled specimen is independent of the orientation of the samples and can be performed in arbitrarily oriented sections [26]. Estimation of surface area- and length density parameters requires generation of sections with randomly oriented section planes [24,25], such as VUR- or IUR sections (**Section 10**, **Table 1**).

Independent of the parameter(s) of interest in a given study, it is in any case strongly recommended to sample and process a sufficient number of supplementary gill samples suitable for analysis of additional quantitative stereological parameters, which might be of interest in the later course of the experiment.

### 5. Adequate killing methods for quantitative stereological gill analyses

The chosen killing method has to conform to the applicable legal animal welfare regulations and must not interfere with the analysis of the experimental results [81,82]. For morphological gill analysis, physical killing methods damaging the gills, such as the commonly used “blow-on-the-head” that often leads to gill hemorrhage, should be avoided. Instead, killing by immersion exposure to overdosed anesthetics, such as buffered tricaine methanesulphonate solution (500 mg/l; pH 6–8.5; Tricaine Pharmaq^®^ 1000 mg/g (Pharmaq Ltd., United Kingdom)) (if compatible with the given study [83,84]) and securing of the euthanasia by subsequent mechanical brain destruction (*e*.*g*., by a thick, sharp trocar) or bleeding (throat-cut) can be recommended.

### 6. Vascular perfusion fixation of the gills

In various experimental medical research disciplines, vascular perfusion fixation of organs and tissues of laboratory animals is standardly used to generate samples for subsequent morphological analyses [85–89]. For quantitative stereological analyses, vascular perfusion fixation is often particularly advantageous, since it removes all cellular blood compartments from the vasculature and causes an *in situ* fixation of the tissue (histo-) morphology and ultrastructure [90,91]. In ecotoxicological studies using trout, vascular perfusion fixation is, for example, used for examination of the liver [92,93] or kidney [94,95]. Vascular perfusion fixation has also frequently been used in several morphometric studies examining the physiology or histomorphology of gills originating from fish of diverse species [37,96]. For vascular perfusion fixation of rainbow trout gills a cardiac perfusion technique is recommendable, whereas retrograde vascular perfusion through the dorsal aorta is often insufficient [97]. The technique of cardiac perfusion fixation of trout gills is comprehensively described and illustrated in **S2 Fig** and elsewhere [97]. However, if not performed properly, vascular perfusion fixation can easily cause severe artifacts in the delicate gills (*e*.*g*., epithelial detachment from the SL due to inadequately high perfusion pressures and flow rates) and substantially impede subsequent qualitative and quantitative morphological analysis (**S3** and **S4 Figs**). Since the quantitative stereological gill parameters featured here can be reliably determined in non-perfusion fixed gills as well, vascular perfusion fixation is not unconditionally recommended for quantitative morphological analysis of the gills.

### 7. Excision and fixation of the gills

**Fig 6:**
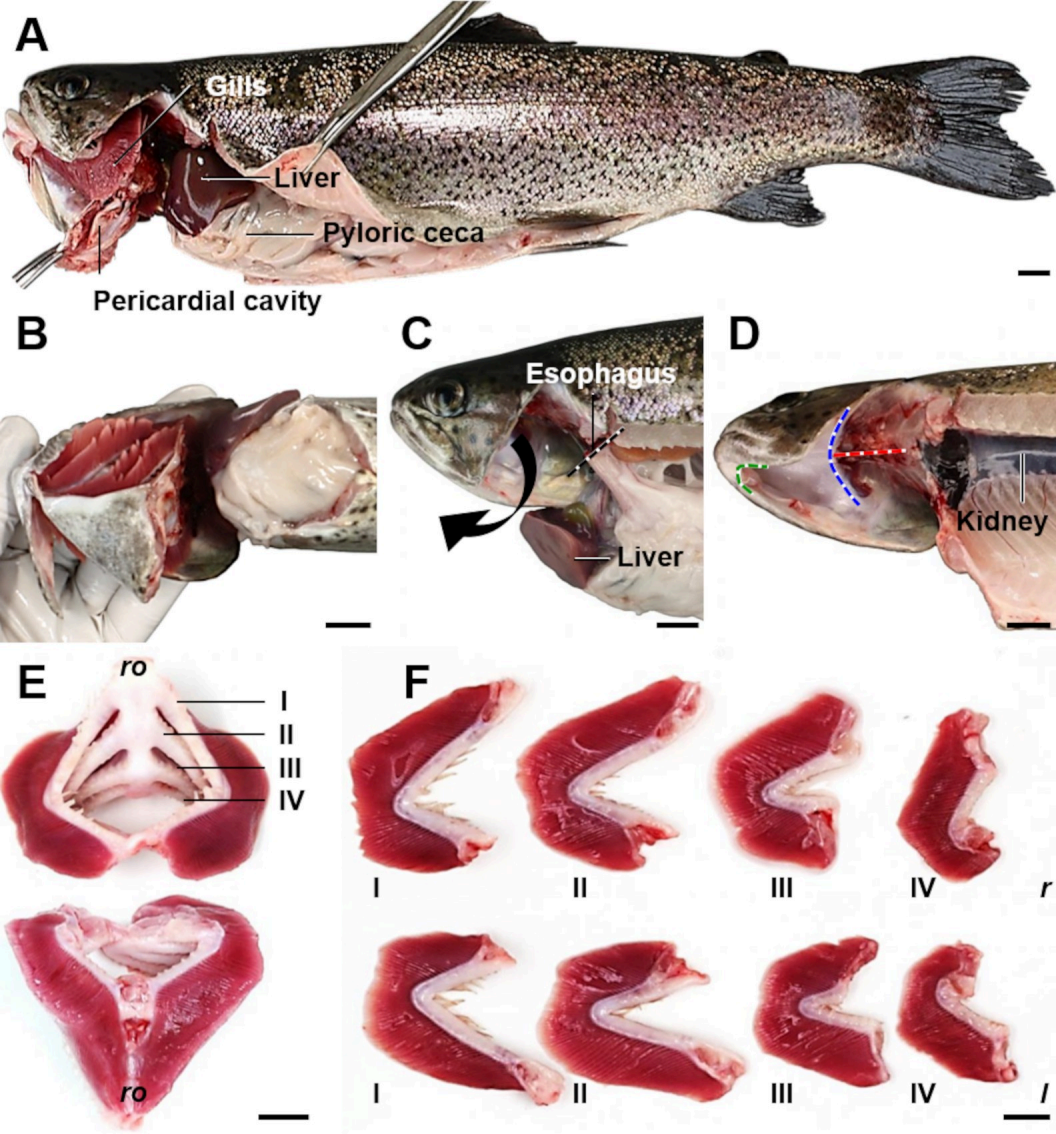
Excision of trout gills. Using scissors, the peritoneal cavity is opened, starting with a transverse incision ventro-caudal to the base of the pectoral fins (**B**). This incision is elongated caudally along the ventral midline, up to a few millimeters cranial of the anogenital papilla (**A**). Just behind the pectoral fins and the cleithrum, the incision is continued in dorso-cranial direction, ending in the opercular chamber by severing the cleithrum (**A**). Then, the operculum is removed and the ventral part of the gill basket is disconnected from the viscerocranium (in **D**, the orientation of the incision line is indicated by a green dotted line). The dorso-cranial connection between the gill basket and the skull is disconnected by severing the rostral pharyngobranchial bones (indicated by a blue dotted line in **D**). Subsequently, the esophagus is cut through (indicated by a black dotted line in **C**), the dorsal connection between the gills and the skull is transected (indicated by a red dotted line in **D**) and the gill basket is removed from the body by gently pulling the gill arches in ventral direction (arrow in **C**). **E.** Dissected gill basket. Top: dorsal aspect. Gills I-IV are indicated. Bottom: ventral aspect. Rostral (*ro*). **F.** Dissected gill arches of the left (*l*) and right (*r*) side, prior to immersion fixation. Bars = 1 cm.

With regard to the fragile histo-architecture of the secondary lamellae, preparation of the gills should be performed immediately after killing. A practicable gill excision technique and subsequent preparation of the gills is illustrated in **Fig 6**. Using scissors, the peritoneal cavity is opened in cranio-caudal direction by a longitudinal incision in the ventral midline, starting with a transverse incision of skin and muscles just behind the pectoral fins. The incision is extended cranio-dorsally to the opercular chamber (with severing of the cleithrum) (**Fig 6A and 6B**). The operculum is removed and the sectioning is proceeded cranially along the medial margins of the mandibular arches. The same procedure is repeated on the other side of the body. The bottom of the oral cavity is severed cranially of the tongue. Then, the dorsal connection of the bony gill skeleton with the viscero-cranium is also cut through (**Fig 6C and 6D**). Subsequently, the esophagus is severed and the entire gill apparatus (together with the heart, adhering parts of the flank and the cranial aspect of the esophagus) is then removed by gently pulling the gill arches in ventral direction (**Fig 6C and 6D**). After removal of the heart and the adjacent muscle tissue, the gills are immediately transferred to neutrally buffered 4% formaldehyde solution (or glutaraldehyde, if appropriate) and fixed for 36 to 48 h at room temperature with gentle agitation.

The filamentous part of the gills represents the appropriate reference compartment that has to be sampled for subsequent quantitative stereological analyses, as far as all relevant quantitative morphological gill parameters refer to gill structures which are only present in the filamentous compartment (*i*.*e*., primary and secondary lamellae). For subsequent analyses the fixed gills are therefore separated and the gill arches with rakers, bones and adherent (non-gill) soft tissues are removed, leaving two rows of gill filaments (*hemibranchs*), connected by the interbranchial septum (for convenience, hereinafter referred to as gill filaments (GF)).

### 8. Determination of the gill filament volume

**Fig 7:**
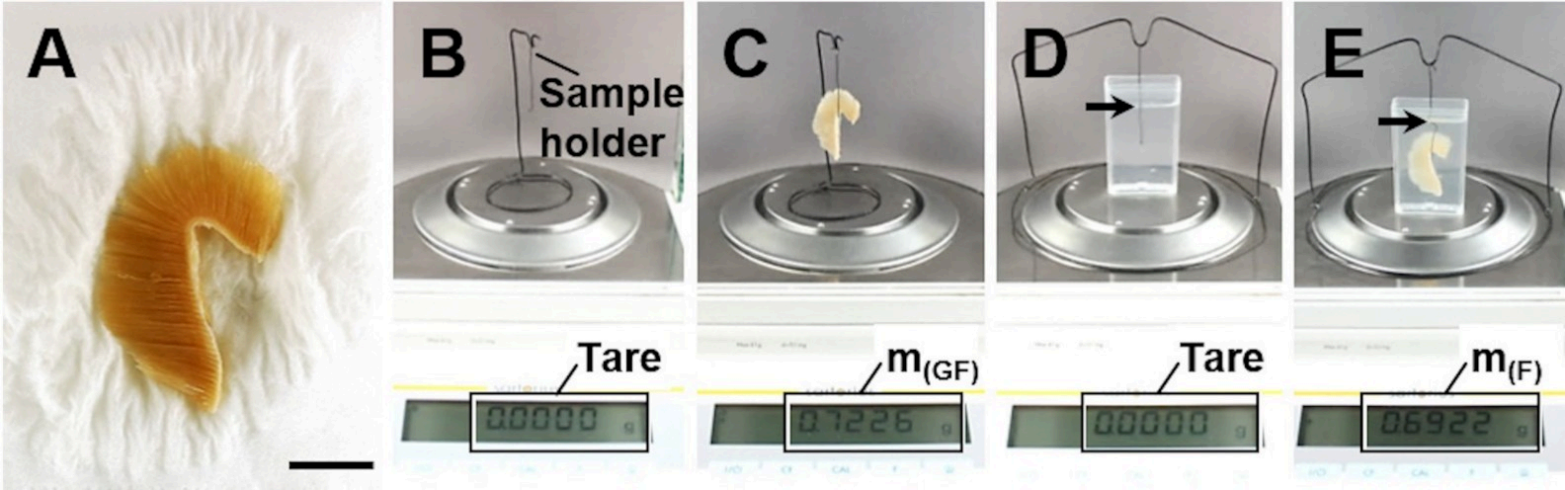
Determination of gill filament density and sample volume, using the submersion method (Archimedes`principle). **A.** Fixed gill after excision of the gill arch, briefly blotted dry on lab-paper towel to remove adhering liquid. **B.** Scale tared to the weight of the sample holder. **C.** Measurement of the GF sample weight (m_(GF)_). **D.** Scale tared to the weight of a container filled with physiological saline or fixative of known density and the submerged sample holder. Note that the sample holder is not placed on the scale sensor and does not have contact to the bottom or the walls of the container. The arrow indicates the position up to which the sample holder is submerged into the liquid. **E.** The sample is attached to the sample holder and completely submerged into the liquid up to the marked position on the sample holder. Note that the sample does not have contact to the bottom or the walls of the container. The weight of the fluid displaced by the GF sample (m_(F)_) is recorded. The sample volume is calculated from the sample weight (m_(GF)_) and the GF density (ρ_(GF)_), using **Eq 1**.

The total volume of the gill filaments (V_(GF)_) is one of the most important parameters in any quantitative stereological study of fish gills, since it provides the reference volume which is essential for calculation of all other absolute quantitative gill parameters (refer to **Sections 1** and **4**). Technically, V_(GF)_ can be determined by different means, *e*.*g*., by submersion of the GF sample in a liquid of known density (ρ). The weight of the liquid displaced by the submerged sample is measured and the corresponding liquid volume (*i*.*e*., the sample volume) is calculated from the weight and the specific weight of the displaced liquid. The same approach is also used to determine the gill filament density (**Fig 7**), which can then be used to calculate the volumes of GF samples from their individual weights [25,26,63,69].

The individual volume of a plastic medium-embedded GF sample can be determined from the histological GF profile areas measured in equidistant, parallel sections covering the entire (known) height of the embedded GF sample (principle of Cavalieri) [14,24,25]. This approach is used, *e*.*g*., for determination of the extent of embedding-related tissue shrinkage, as described in **Section 11**.

For application of the submersion technique, the (fixed or fresh) GF specimen is gently dabbed dry, using a lab-paper towel to remove any liquid adhering to the gill filaments. Subsequently, the sample is weighed to the nearest mg. The volume of the sample is then determined from the volume of liquid of known density which is displaced by the completely submerged specimen (**Eq 1**), as illustrated in **Fig 7E**. A transparent container is filled with physiological saline (ρ = 1.0046 g/cm^3^ at room temperature) or fixation solution of known density and placed on the scale. A sample holder is submerged into the liquid to a defined position without any contact to the container or the scale, and the scale is then tared. The GF sample is attached to the sample holder and completely submerged up to the marked position on the sample holder. It is important to ensure that the submerged sample does not have contact to the walls or the bottom of the container. The weight of the liquid that is displaced by the submerged sample is recorded (m_(F)_). The total volume of the GF sample (V_(GF)_) and its density (ρ_(GF)_) is calculated from the weight of the GF sample (m_(GF)_) and the density (ρ_(F)_) and weight of the displaced liquid (m_(F)_) (**Eq 1**). In studies examining large numbers of fish, it is not necessary to determine the density of all individual GF samples. Instead, the average gill filament density is determined in an appropriate number of representative samples per experimental group and collectively used for calculation of the individual sample volumes from their individual weights [25].

Using the described technique, we determined the average gill filament density (formalin-fixed) on 12 GF samples from healthy rainbow trout to account for 1.07 ± 0.02 g/cm^3^ (mean ± standard deviation (SD)).

**Eq 1**. **Calculation of the total gill filament volume (submersion method)**.

$$V_{\left(GF\right)}=m_{\left(GF\right)}/\rho_{\left(GF\right)}$$

**V**_**(GF)**_ Total volume of the gill filament (GF) sample

**m**_**(GF)**_ Weight of the GF sample (blotted dry)

**m**_**(F)**_ Weight of the fluid (F) displaced by the submerged GF sample

**ρ**_**(GF)**_ Density of the GF sample (ρ_(GF)_ = m_(GF)_/V_(gf)_)

**ρ**_**(F)**_ Fluid density at 20° C

**V**_**(gf)**_ Volume of the fluid displaced by the submerged GF sample

(V_(gf)_ = m_(F)_/ρ_(F)_)

Due to their filamentous lamellar structure, gills do have an extraordinarily high water-binding capacity. Volume determination of gill samples using the submersion technique may therefore be substantially biased by the amounts of liquid adhering to the gill filaments [69]. The volume of liquid attached to the (moist) gill sample must therefore be considered when the sample volume is determined and adequate removal of this liquid (without damaging the gills) must be guaranteed. The exact volume of liquid attached to a gill specimen can experimentally be determined by photometric measurement of the decrease of the concentration of a dyed liquid, which is diluted by the (unstained) liquid attached to a moist gill sample that is submerged in the dyed liquid. The experiment is illustrated and described in detail in **S5** and **S6 Figs**. Using this experimental approach, we tested the efficiency of the removal of the liquid attached to moist gill samples by gently dabbing the samples with lab-paper towels (**Figs 7A, S6G**). In seven tested gill samples, the volume of liquid adhering to the samples (determined as described above) and the volume of water that was removed from the samples by placing them on a lab-paper towel for approximately 10 seconds was not significantly different (mean deviation: 1.78 ± 0.01%, *p* = 0.736, paired t-test). Therefore, the volume of gill samples can be adequately determined with the submersion technique, if the samples are carefully dabbed dry in advance.

### 9. Systematic uniform random (SUR) sampling of representative gill filament samples

After volume determination, gill filament (GF) (sub-) samples are taken for quantitative stereological analyses of the morphological parameters of interest. Assuming that the gills of the right and the left side do not differ systematically in their histo-architecture, it is sufficient to randomly sample either the left or the right gills (**S1A Experimental data**).

The generated GF samples must be representative, *i*.*e*., adequately reflect the morphological properties of the entire reference compartment (*i*.*e*., the entirety of gill filaments). Representative GF samples can be generated using efficient systematic uniform random (SUR) sampling designs [63,85,98], as described below. The applied SUR sampling design ensures that every possible location in the gill filaments is sampled with the same random probability, which is a crucial prerequisite to obtain precise and unbiased estimates of quantitative parameters in the subsequent stereological analyses [25,26,59,98].

**Fig 8:**
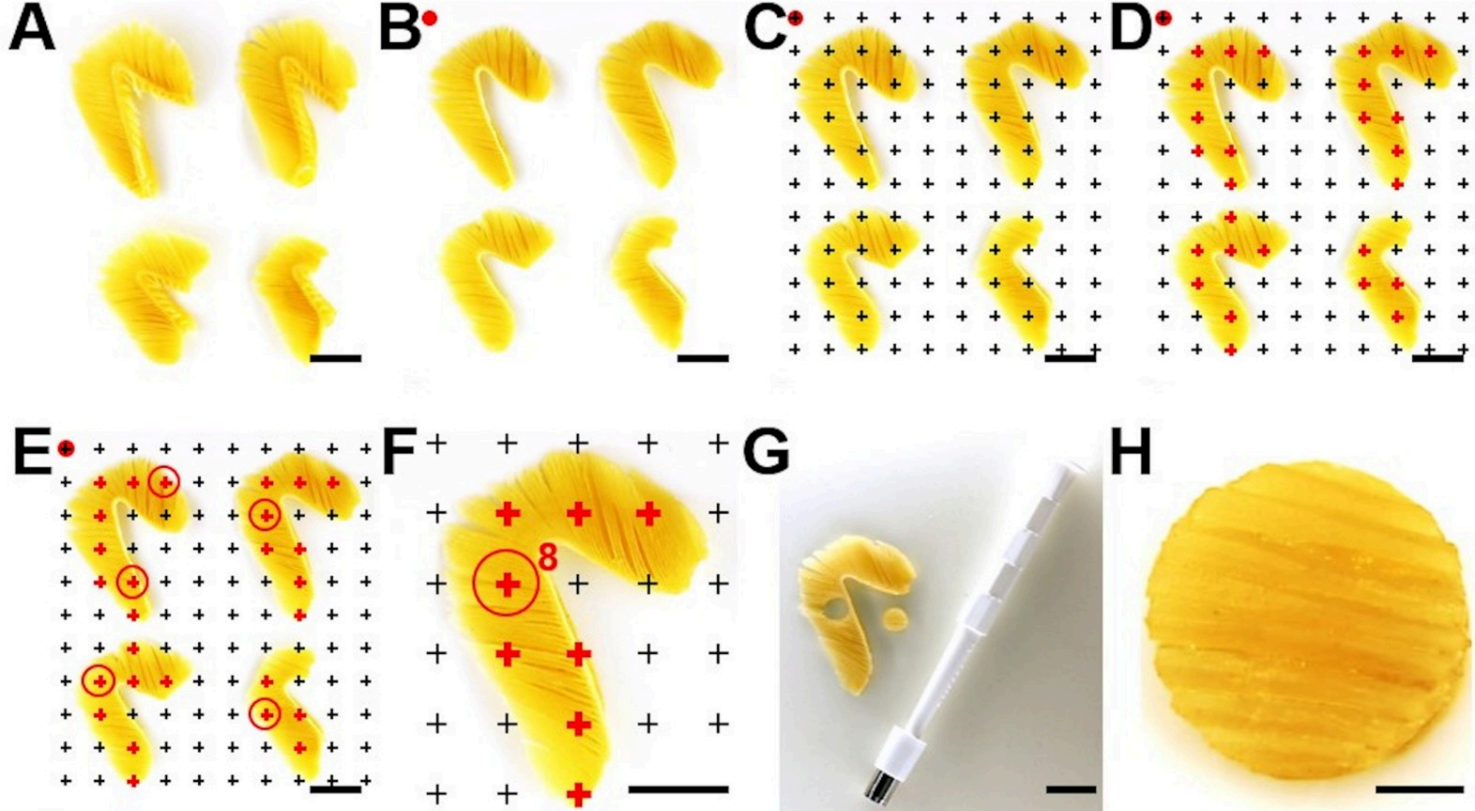
Systematic uniform random (SUR) sampling of representative gill filament samples. **A.** The 4 formalin-fixed gills of one side before the removal of the gill arches. **B.** After removal of the gill arches, the gills are placed on their opercular (*i*.*e*., lateral) sides. Optionally, the GF are briefly dipped in liquid agar for stabilization. **C.** The GF are randomly superimposed with an appropriately sized cross-grid printed on a plastic transparency. Here, a 6 mm grid is used. To randomize the position of the grid relative to the gills, the upper left cross of the grid is placed over a random point outside of the GF (indicated by a red spot). **D.** All crosses hitting the GF are marked (red crosses). In the present example, 27 crosses hit the GF. **E.** The sampling interval (i) is defined by the number of crosses hitting the sampled reference compartment (n) and the number of SUR samples to be generated (s) for analysis of a specific quantitative stereological gill parameter (i = n/s). In the present example, ≥ 5 samples are to be generated. Accordingly, every 5^th^ position (27/5 = 5.4) where a cross hits the GF is sampled. The position of the first location to be sampled is determined randomly within the sampling interval (1-i; here position N°3), using a random number table/generator. Thus, in the present example 5 GF locations are SUR sampled (N°3, N°8, N°13, N°18, N°23), as indicated by red circles. (Counting proceeds from the left to the right and from top to bottom.) **F.** Detail enlargement of the second gill with the sampling location N°8, indicated by the red circle. **G.** SUR sampled GF sites are excised, using a 6 mm biopsy punch. **H.** Detail enlargement of an excised SUR sampled GF specimen. Bars = 1 cm in A-G and = 0.25 cm in H.

SUR sampling of the reference compartment (GF) for subsequent quantitative stereological analyses of the volume fractions of the relevant quantitative gill parameters (V_V(SL/GF)_, S_V(SL/GF)_, N_V(EC/SL)_) is described below and illustrated in **Fig 8B–8G**. The absolute quantitative parameters of distinct structures/cells of the gill filaments (*e*.*g*., V_(SL,GF)_, S_(SL,GF)_, N_(EC,SL)_) are calculated from their relative volume fractions and the total GF volume (V_(GF)_). V_(GF)_ is determined by submersion volumetry/weighing of the gill filaments after removal of the gill arch as described in **Section 8**.

To prevent a loss of gill filaments and to preserve the orientation of the primary gill lamellae in the excised specimen, it is recommended to stabilize the gill filaments by briefly dipping the gills in liquid agar prior to sampling. For SUR sampling, the gills are then placed on their opercular side and randomly overlaid with a cross-grid, printed on a transparent plastic. For SUR sampling of gill filaments in the gills of trout of approximately 300–2000 g of body weight, a cross grid with 4–6 mm lateral distance between two adjacent crosses can be recommended (depending on size of the gills, the diameter of the biopsy punch, and the percentage of the gill filaments that should be sampled). Copy templates of cross grids of diverse sizes can be found at Albl et al. [85] or Howard and Reed [25]. All crosses hitting the gill filaments are counted (a cross is counted as a hit if the right upper corner of the cross hits the gill filaments [26,63,85]), and sampling localizations are chosen systematically according to a defined sampling interval (i). The sampling interval (i) is defined by the number of crosses hitting the sampled reference compartment (n) and the number of SUR samples (s) to be generated (i = n/s). The numbers of samples recommended for analysis of different quantitative morphological gill parameters are given in **Table 3**. The first sampling location is determined randomly within the sampling interval (1-i) using a random number table/generator [24,25,63,85]. The sampled locations are marked, *e*.*g*., with blank paper confetti, and the samples are excised using a biopsy punch of 4.0–6.0 mm diameter (Stiefel Biopsy punch, SmithKline Beecham Ltd., United Kingdom). Subsequently, the SUR sampled GF specimens are differentially processed, according to the specific histo-technical requirements of the respective quantitative stereological analysis methods that are applied for examination of the different morphological parameters of interest (as illustrated in **Fig 5**). An alternative, efficient sampling and embedding procedure allowing for estimation of the gill volume and the gill surface area is comprehensively illustrated in da Costa et al. [38].

**Table 3:** Recommended sampling design and sample number for quantitative stereological analysis of different morphological gill parameters.

Parameter	Number^a^ of SUR samples	Sample processing		Paper Section
		Sample orientation	Embedding medium	
**V**_**V(SL/GF)**_	5	Arbitrary	Paraffin^**b**^	12
**S**_**V(SL/GF)**_	5	VUR^**c**^	GMA/MMA	13
**V**_**V(EC/SL)**_**, N**_**V(EC/SL)**_, ${\bar{v}}_{\left(\mathrm{EC},\mathrm{SL}\right)}$	5	IUR^**d**^	Epon^**e**^	14
**T**_**h(DB)**_	5	IUR	Epon	15

^**a**^The indicated sample numbers refer to the gills of one body side and represent orientation values based on a previous study, examining trout with body weights of ~300 g [99]. In a given study, the number of samples may have to be individually adapted to the specific experimental settings and examined parameters.

^**b**^Paraffin-embedding facilitates identification of distinct tissue structures or cell types by immunohistochemistry or special histological staining.

^**c**^Estimation of S_V(SL/GF)_ is performed on VUR sections, since a "preferred" sample orientation can be obtained thanks to the unrestricted choice of VA orientation. The estimation of S_V(X/Y)_ on VUR sections is the method of choice for most design-based studies [25].

^**d**^The generation of IUR sections of SUR sampled and Epon-embedded GF samples is highly recommended for estimation of N_V(EC/SL)_, since all other relevant quantitative morphological gill parameters can be estimated on these sections, if necessary.

^**e**^Epon-embedding enables for preparation of semithin serial sections or ultrathin sections for TEM analysis.

The generation of 5 GF samples of the four gills from either the left or the right side is considered as sufficient for an efficient, unbiased estimation of the relevant quantitative stereological gill parameters (total coefficient of variance (CV) = 1.7% [25]) (**S1B Experimental data**). However, a higher number of samples might be necessary in experimental settings where the distribution of lesions in the experimental group may be irregular.

### 10. Randomization of the orientation of the sample section plane

Quantitative stereological analyses of surface areas and lengths of distinct tissue structures in histological sections depend on the 3-D orientation of the analyzed tissue structures relative to the orientation of the 2-D section plane(s) [25,26]. Therefore, the spatial orientation of the analyzed sections (relative to the examined samples or *vice versa*) has to be randomized in quantitative stereological analyses of these parameters (**Tables 1** and **3**, **Figs 1** and **5**) [25,68].

For an efficient randomization of the orientation of a section plane cut through an individual sample, different methods have been developed [25,62,68,100,101]. In isotropic uniform random (IUR) sections, the orientation of the sample (respectively of the section plane cut through the sample) is randomized in all three dimensions of space, IUR sections can be used for analysis of all quantitative stereological parameters [14,24,68,102,103]. However, since the spatial orientation of each individual IUR section is completely random, the histological appearance of IUR sections is variable, and often divergent from the “familiar” histology the pathologist is used to [24]. This is especially relevant for organs with a highly anisotropic histo-architecture, such as fish gills (**Figs 2** and **3**).

**Fig 9:**
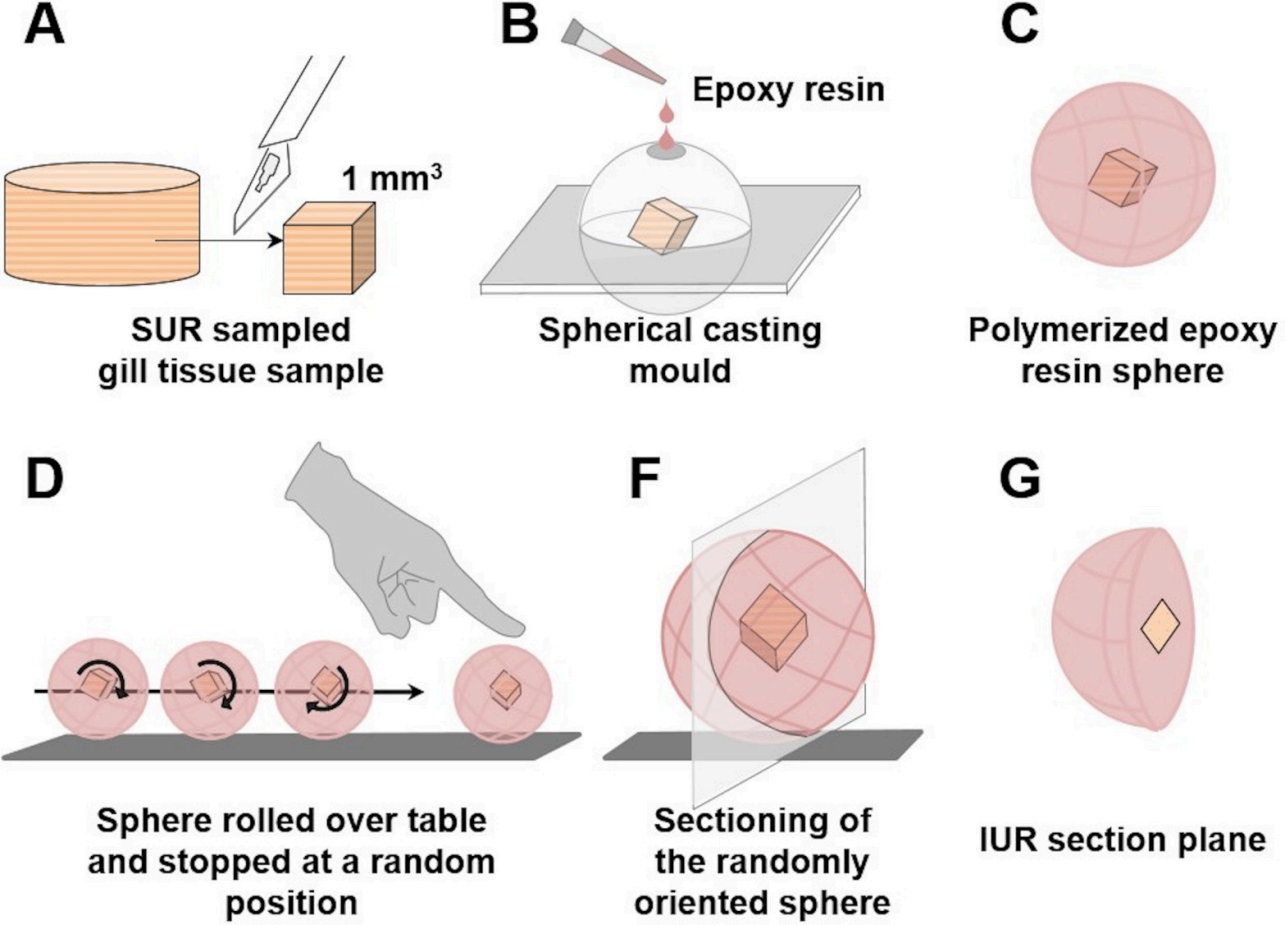
Generation of IUR sections of a SUR sampled gill filament specimen with the *Isector* method. **A.** A SUR sampled specimen of fixed gill filaments is carefully cut to a size of approximately 1 mm x 1 mm x 1 mm (suitable for Epon-embedding and electron microscopy), preserving the secondary lamellae as structure of interest. **B.** The specimen is embedded in a sphere of epoxy resin, using a spherical casting mould. **C, D.** After polymerization of the embedding medium, the sphere is rolled across a flat surface and stopped at a random position. **F, G.** The sphere is sectioned at this random position, resulting in an IUR section plane.

IUR sections can principally be generated using different approaches, such as the *Orientator* [63,85,101] or the *Ortrip* method [100]. For generation of IUR sections of (small) gill filament (GF) samples, the one-cut *Isector* method [68] has proven suitable, as illustrated in **Figs 9** and **S7**. Generation of IUR sections with the *Isector* is reasonably easy: SUR sampled specimens are embedded in (isotropic) Epon spheres, using spherical casting molds. If electron microscopy is to be performed, the SUR sampled GF samples are previously trimmed to a size of ~1 mm^3^. After polymerization, the spherical sample is rolled across the workbench surface, stopped at a random position and sectioned at this position to receive an IUR section plane [63,85].

**Fig 10:**
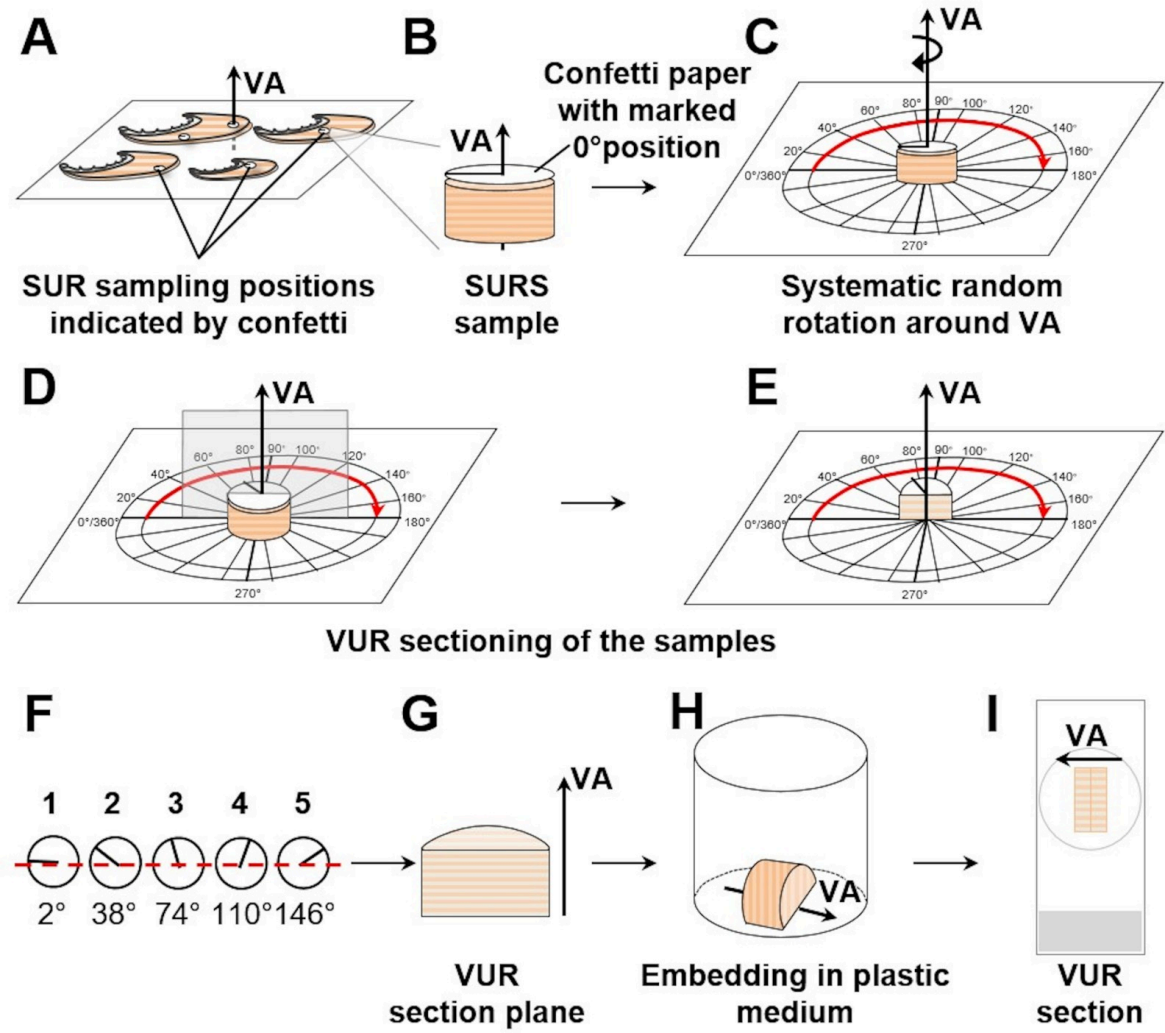
Generation of VUR sections of a SUR sampled gill filament sample. **A.** SUR sampling positions on the GF are marked by confetti paper. The vertical axis (VA) is indicated. **B.** SURS sample excised with a biopsy punch. The orientation of the sample relative to the gill is marked by a black line on the confetti paper (0°-180°-line). **C.** The sample is placed on an equiangular circle, corresponding to the 0°-180°-mark on the confetti paper. **D-G.** The first SURS specimen is randomly rotated around the VA by an angle between 0° and 36°, determined using a random number generator (here: 2°). The following four samples are systematically rotated around their VA in a predefined rotation interval of 36° (here: 38°, 74°, 110°, and 146°). The samples are sectioned at the corresponding positions (parallel to the VA). **H.** The samples are embedded in plastic medium (*e*.*g*., GMA/MMA), maintaining the orientation of their VUR section surfaces, the VA is still identifiable in light microscopy. **I.** The resulting histological sections are VUR sections, used for analysis of the surface area densities of the secondary gill lamellae in the gill filaments (S_V(SL/GF)_), as described in **Section 13**.

Surface area densities, such as the surface area density of the secondary lamellae in the gill filaments (S_V(SL/GF)_) can be efficiently analyzed in vertical uniform random (VUR) sections [62]. In VUR sections a fixed vertical axis (VA) of the sample is defined, by which the orientation of the section through the sample is only randomized in the two remaining directions of space. The VA can be freely chosen, as long as it is clearly recognizable in all sections. This allows generation of histological sections with a more “habitual” appearance, facilitating quantitative stereological analyses of surface area density parameters [62,104]. For generation of VUR sections of SUR sampled GF specimens, the technique shown in **Figs 10** and **S8** is recommended. SUR sampling of 5 GF specimens is performed as described in **Section 9**. VA is defined as the axis perpendicular to the gills placed on the flat workbench with their opercular side (*i*.*e*., the horizontal plane). The original orientation of the SUR GF samples (relative to the gill) is marked on paper confetti placed on the samples (**Figs 10B and 10F** and **S8A–S8C**). The excised samples are then systematically rotated around their VA in a predefined rotation interval (i) of 36° (*i*.*e*., 180°/5 SUR sampled specimens (s)), with the first sample being rotated at a random angle within the rotation interval (**Figs 10C–10F** and **S8B and S8C**). To receive VUR GF section planes, the systematically randomly rotated samples are vertically cut through (parallel to their VA) in their respective orientations (**Figs 10E–10G** and **S8D and S8E**). Maintaining the orientation of their VUR section planes, the samples are then embedded in a histological plastic embedding medium, sectioned and stained (**Fig 10G–10I**).

### 11. Determination of plastic embedding-related three-dimensional shrinkage of the gill filaments

**Fig 11:**
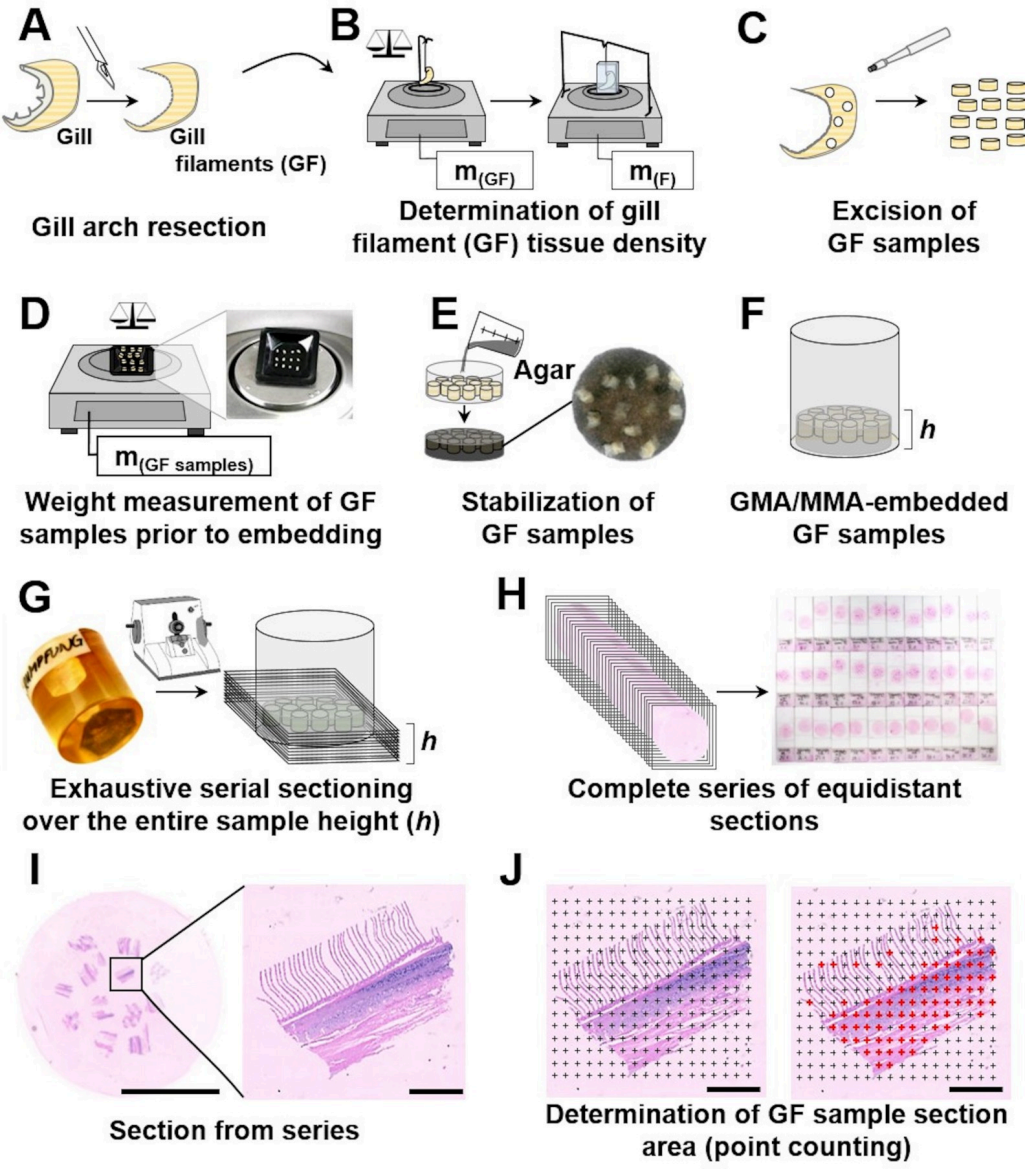
Determination of volume shrinkage of gill filaments due to embedding in plastic embedding media. **A-D. Volume determination of SUR sampled GF samples prior to embedding.** After dissection of the gill arch (**A**), the density of the (fixed) GF samples is determined (**B**). The GF samples are excised by biopsy punch of 0.2 cm diameter (**C**) and the samples weight is recorded (**D**). GF sample volume is calculated from GF density and sample weight (submersion technique, refer to **Section 8** and **Eq 1**). **E-H. Embedding of GF samples in plastic medium and exhaustive serial sectioning of the embedded samples.** For stabilization and visual contrast, the GF samples are embedded in ink-dyed black agar (**E**) and subsequently routinely processed and embedded in plastic embedding medium (**F**) (here: GMA/MMA). The embedded samples are then exhaustively sectioned over the entire sample height (h) with a defined section thickness (**G**). From the section series (**H**), sections are taken in a defined interval (*e*.*g*., every 40^th^ section) and mounted on a glass slide. The factual thicknesses of the individual sections are determined by spectral reflectance measurement (not shown) [80]. **I-J. Determination of GF sample section areas in equidistant serial sections.** The section profile areas of all samples in all examined sections are determined (here: point counting). Digital microscopic section images are randomly overlaid with a grid of equally spaced test points (crosses) of known distance at the given magnification (*i*.*e*., every point (P) is associated with a defined area (A)). The number of points hitting GF section profiles are counted. Since the entire height (h) of the GF samples was sectioned into equidistant, parallel sections, the volume of the embedded GF samples can be calculated according to the principle of Cavalieri [24,59,63], from the total section profile area of all samples in all sections (A_(GF sample section profiles)_) and the mean distance between two examined sections (*i*.*e*., section thickness x section interval). A_(GF sample section profiles)_ is calculated from the total number of counted points (∑P) and the area associated with each point (A/P) (refer to **Eqs 2** and **4**). The proportional volume shrinkage of GF samples associated with the embedding in the histological plastic embedding medium is calculated from the quotient of the sample volume prior to and after embedding. The linear tissue shrinkage factor (f_s_) for gill filaments embedded in plastic medium is calculated as shown in **Eq 3**. Bar = 1 cm in I (left image side) and = 500 μm in I (right image side) and J.

Embedding of samples and preparation of histological sections is generally associated with a 3-D shrinkage of the samples. The extent of shrinkage depends on the tissue, the embedding medium, as well as on the sample size and -volume [14,26,61,64]. Plastic-resins, such as GMA/MMA or Epon, are commonly used as histological embedding media for quantitative analyses of morphological tissue parameters affected by embedding-related tissue shrinkage, since the embedding-related tissue shrinkage is lower and more uniform, as compared to paraffin [14,61,64,79,80]. To obtain unbiased quantitative estimates of shrinkage-sensitive parameters, such as surface area-, length- and numerical volume densities, the extent of the embedding-related tissue shrinkage has to be considered in quantitative stereological studies [26]. The extent of the 3-D embedding-related tissue shrinkage is determined by comparing the sample volumes before and after embedding (**Fig 11**). Assuming a uniform shrinkage in all three dimensions of space, the embedding-related shrinkage of solid tissue samples (*e*.*g*., liver) can conveniently be determined by comparison of the areas of corresponding organ/tissue section surfaces prior to and after embedding [63,64,80]. For gill samples, however, this approach is not applicable because of the microscopic lamellar architecture of the gill filaments (GF). Instead, the volumes of fixed GF samples are determined prior to and after embedding [71]. The volume of the fixed GF samples is determined directly from their weight and density (refer to **Section 8** and **Fig 11A–11D**). The GF sample volume after embedding in a plastic embedding medium is determined according to the principle of Cavalieri [24,25,59]. For this purpose, the embedded samples are exhaustively sectioned (*i*.*e*., over the entire sample height) into equidistant, parallel sections (**Fig 11E–11H**). The volume of the embedded samples is calculated from the cumulative section profile area of the samples in all examined sections and the average distance between two consecutively examined sections (**Eq 2**). The GF section profile areas can be determined, *e*.*g*., by point counting, as shown in **Fig 11J**. The linear tissue shrinkage factor (f_s_) used for shrinkage correction of quantitative stereological estimates of surface area-, length- and numerical volume densities is calculated according to **Eq 3** [26,63], the adequate application of f_s_ for the relevant quantitative morphological parameters is given in **Table 1**.

Note that the extent of embedding-related tissue shrinkage does not have to be determined for each individual sample. Instead, the average extent of embedding-related shrinkage determined for identically processed samples of the same organ/tissue and a specific embedding medium are concordantly used for shrinkage correction in a given study. In own experiments, we determined f_s_ for formalin-fixed, GMA/MMA-embedded rainbow trout gill filaments to account for f_s_ = 0.87, corresponding to a volume shrinkage of 34.31% (**S1C Experimental data**).

**Eq 2**. **Volume of plastic-embedded gill filament samples**.

$$V_{\left(embedded\;GF\;samples\right)}=d\times \sum A_{\left(GF\;sample\;section\;profiles\right)}$$

**V**_**(embedded GF samples)**_ Stereologically estimated volume of gill filament (GF) samples embedded in histological plastic embedding media

**d** Mean distance between two adjacent examined sections (d = h/n)

**h** Sample height (*i*.*e*., the length of the sample axis perpendicular to section plane orientation, calculated from factual mean individual section thickness and total number of sections)

**n** Number of (parallel, equidistant) examined sections (*i*.*e*., sections are SUR sampled for GF section profile area estimation by point counting in a predefined sampling interval)

**∑A**_**(GF sample section profiles)**_ Cumulative sample section profile area of the gill filament samples in all examined sections per case

**Eq 3**. **Linear tissue shrinkage factor for plastic-embedded gill filament samples**.

$$f_{s}=\sqrt[{3}]{V_{\left(embedded\;GF\;samples\right)}/V_{\left(GF\;samples\right)}}$$

**f**_**s**_ Linear tissue shrinkage factor

**V**_**(embedded GF samples**)_ Stereologically estimated volume of gill filament (GF) samples embedded in histological plastic embedding media

**V**_**(GF samples)**_ Volume of (fixed) GF samples prior to embedding

### 12. Estimation of volume densities and total volumes of distinct gill filament structures

**Fig 12:**
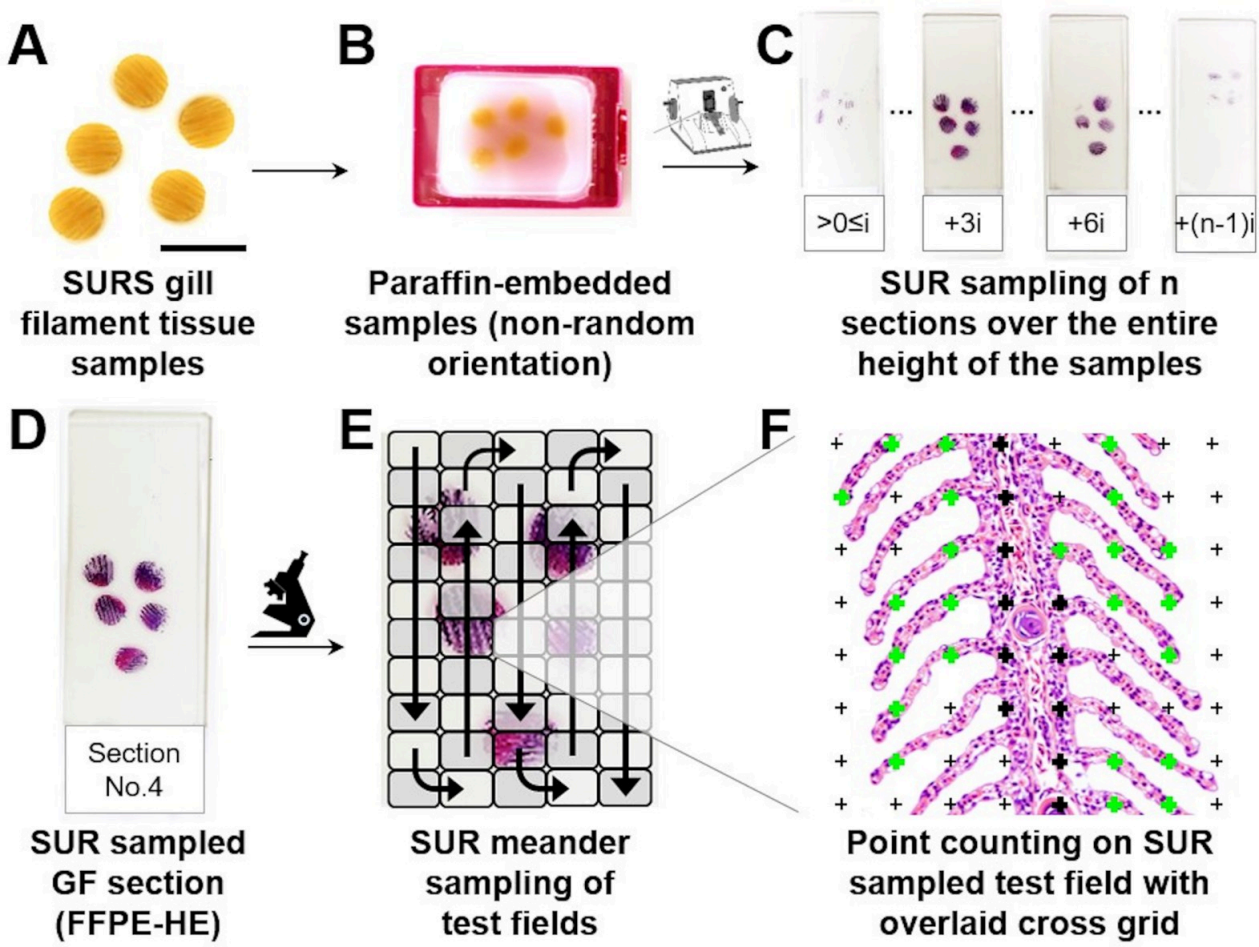
Estimation of the volume density of the secondary lamellae in the gill filaments. **A.** SUR sampled GF specimens (please compare to **Section 9**). **B, C. SUR sampling of sections from samples embedded in paraffin in non-random orientation. B.** Paraffin block with sagittally embedded GF samples. **C.** SUR sampling of sections. The entire block is exhaustively sectioned. For subsequent analyses, sections are taken in a defined sampling interval (i). The first section is randomly taken within the sampling interval (>0≤i). **D-G. Estimation of V**_**V(SL/GF)**_ **by point counting. D.** SUR sampled section of GF specimens. **E.** SUR sampling of test fields within the section, performed at a factor of magnification allowing for a reliable differentiation of PL and SL (*e*.*g*., 40x-100x microscopic magnification). All sampled sections per case are entirely screened, following a defined meander pattern and test fields are SUR sampled in a defined interval i (*i*.*e*., every i^th^ field of view containing GF section profiles), with the first field of view being randomly selected within the sampling interval. **F.** SUR sampled test field overlaid with an appropriately sized cross grid (here: 8x8 points at 100x microscopic magnification). The number of points hitting GF section profiles (P_(GF)_, indicated by bold crosses) in all sections per case are counted, as well as the number of points hitting SL section profiles (P_(SL)_, indicated by green crosses). 34 points hit the entire GF section profile, 23 points hit the SL section profile. V_V(SL/GF)_ is calculated as the point density of P_(SL)_ and P_(GF)_. The total SL volume (V_(SL,GF)_) is calculated as the product of V_V(SL/GF)_ and the GF volume V_(GF)_.

If appropriate SUR sampling designs are applied for the selection of sampled organ/tissue locations, blocks, sections and section areas, the volume densities of distinct gill structures (*e*.*g*., secondary lamellae) within their corresponding reference compartments (*e*.*g*., gill filaments) can unbiasedly be estimated from their section profile areas (principle of Delesse) [25,26,59,105]. According to Delesse, the unbiased estimate of the quotient of the section profile areas (estimated by point counting as illustrated in **Section 11**) of a structure of interest (X) and its corresponding reference compartment (Y) (*i*.*e*., the section area density A_A(X/Y)_) is an unbiased estimate of the volume density V_V(X/Y)_ (*e*.*g*., V_V(SL/GF)_) (**Eq 4**) [25,26,105]. The total volume of the structure of interest is then calculated from its volume density in the reference compartment and the total volume of the reference compartment (**Eq 5**). The applied SUR sampling designs as well as the processing and analysis procedures featured in the present guidelines ensure that the estimates of the different volume density parameters are independent of the shape and distribution of both the gill reference compartments and the analyzed gill structures, despite the highly anisotropic spatial histo-architecture of the gills. Within the sampled sections and test fields, A_A(X/Y)_ can be estimated by point counting, using points as non-direction sensitive stereological probes (**Figs 11J** and **12F**) [25,26,59]. For this, the SUR sampled section test fields are randomly superimposed with a grid of equally spaced crosses (points). The number of points hitting section profiles of the structure of interest, as well as the number of points hitting the reference compartment within all examined test fields of all sections of all samples of a case are counted and used to calculate A_A(X/Y)_ (**Eq 4**).

**Eq 4**. **Volume densities of distinct gill structures**.

$$V_{V(X/Y)}=A_{A(X/Y)}=\sum A_{\left(X\right)}/\sum A_{\left(Y\right)}=\sum P_{\left(X\right)}/\sum P_{\left(Y\right)}=P_{P(X/Y)}$$

**V**_**V(X/Y)**_ Volume density of the structure X in the reference compartment Y

**A**_**A(X/Y)**_ Area density of the structure X in the reference compartment Y

**∑A**_**(X)**_**/∑A**_**(Y)**_ Quotient of the cumulative section area of the structure X in all examined reference compartment sections per case and the cumulative section area of the reference compartment Y in the same sections

**∑P**_**(X)**_**/∑P**_**(Y)**_ Quotient of the total number of points hitting section profiles of the structure X in all examined sections per case and the total number of points hitting the reference compartment Y in the same sections

**P**_**P(X/Y)**_ Point density of the structure X in the reference compartment Y

**Eq 5**. **Total volumes of distinct gill structures**.

$$V_{\left(X,Y\right)}=V_{V(X/Y)}\times V_{\left(Y\right)}$$

**V**_**(X,Y)**_ Total volume of the structure X in the reference compartment Y

**V**_**V(X/Y)**_ Volume density of the structure X in the reference compartment Y

**V**_**(Y)**_ Total volume of the reference compartment Y

As a dimensionless parameter, volume densities are generally independent of the effect of embedding-related (homogenous) tissue shrinkage and can thus be analyzed in standard paraffin sections (**Fig 12**) [26]. This also facilitates the use of a variety of different histological stains, as well as identification of specific tissue structures by immunohistochemistry [106]. Therefore, if a randomization of the section plane orientation of SUR sampled gill filament (GF) specimens is not performed (**Table 1**, **Fig 5**), paraffin-embedded samples can be exhaustively sectioned in parallel, approximately equidistant sections of arbitrary orientation (for gill filaments, sagittal section plane orientation relative to the gill filaments may be convenient), and a subset of individual sections is systematically randomly sampled from the section series for subsequent estimation of V_V(X/Y)_ (**Fig 12C**). In contrast, exhaustive sectioning and SUR sampling of sections from one sample block is not necessary, if V_V(X/Y)_ is determined in VUR- or IUR sections of (plastic-embedded) GF samples, generated for estimation of additional quantitative morphological gill parameters, *e*.*g*., numerical volume- or surface area densities (**Table 1**, **Fig 5**), which considerably increases the analysis efficiency. Within the sampled sections, test fields (*i*.*e*., fields of view at the appropriate factor of magnification/objective) are SUR sampled, *e*.*g*., by a meander sampling approach as illustrated in **Fig 12E** or by SUR sampling with suitable stereology software tools, as comprehensively exemplified in Monteiro et al. [21]. For a reliable differentiation of secondary and primary gill lamellae and an efficient point counting process, a microscopic magnification of 40x-100x is recommendable. The SUR sampled fields of view are superimposed with an appropriately sized point grid. The number of points hitting section profiles of secondary lamellae (P_(SL)_) in all sampled test fields of all sections per case are counted, as well as the number of points hitting GF section profiles (P_(GF)_). V_V(SL/GF)_ is calculated from P_(SL)_ and P_(GF)_ using **Eq 4**. The minimal total number of points hitting the reference compartment (per case) that is necessary to achieve a V_V(X/Y)_ estimate with a defined acceptable expected relative error probability, can be obtained from a nomogram published by Weibel [26]. For a V_V(SL/GF)_ of ~0.3 (**S1D Experimental data**), a number of ~600 points hitting GF section profiles (in all examined fields of view in all sections of all samples per case) is sufficient to achieve an estimate of V_V(SL/GF)_ with an expected relative error probability of 5% of the mean V_V(SL/GF)_ (**S1E Experimental data**)_._

The total volume of the secondary gill lamellae (V_(SL,GF)_) is calculated as the product of V_V(SL/GF)_ and the total gill filament volume V_(GF)_, which is directly determined, as described in **Section 8**. The volume densities and volumes of other structures within the gill filaments, such as distinct cell types, can be determined analogously, using appropriately adapted magnification factors, sampling intervals, and point grid sizes.

### 13. Estimation of the surface area of the secondary lamellae in the gill filaments

**Fig 13:**
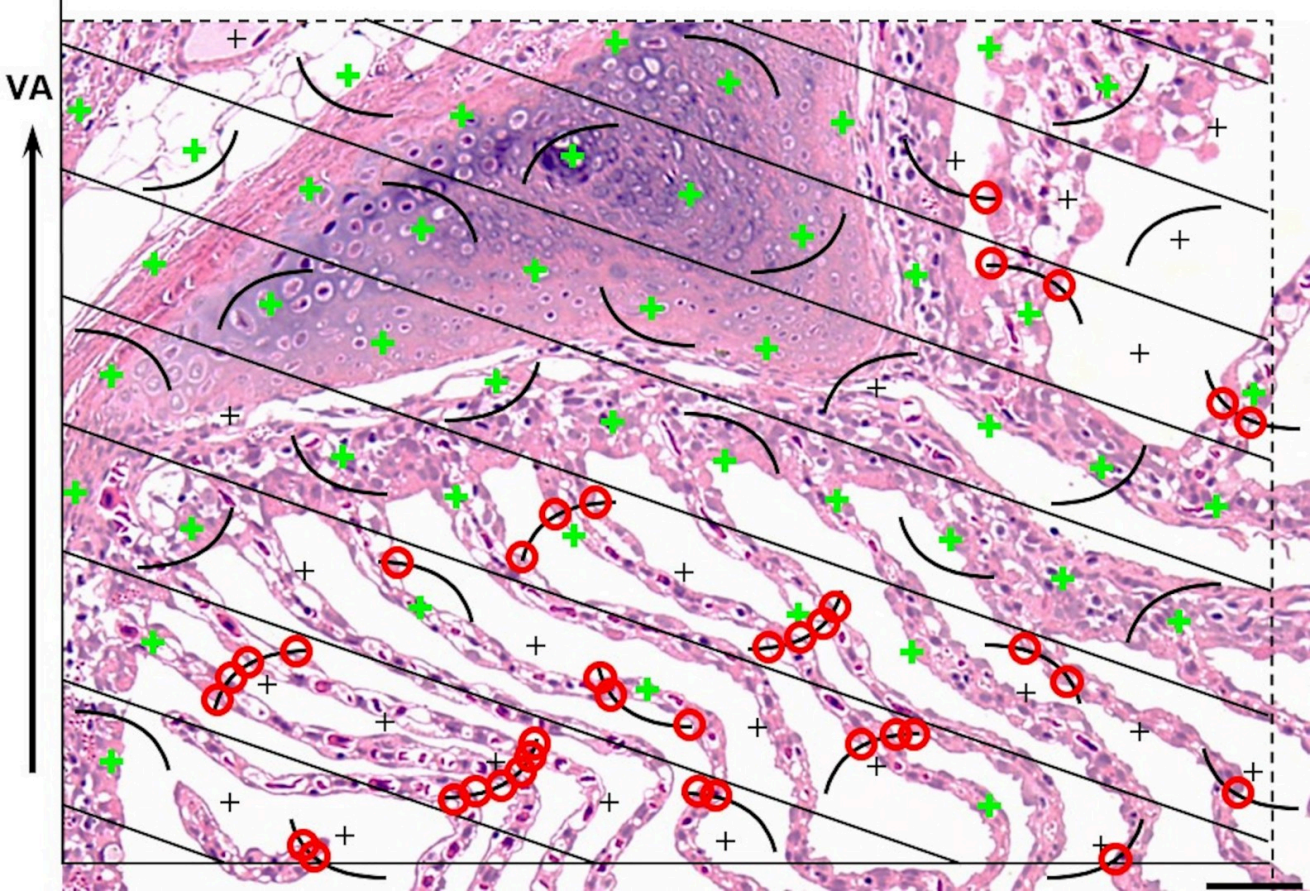
Estimation of the surface area density of the secondary lamellae in the gill filaments. A SUR sampled microscopic test field in a VUR section of a GMA/MMA-embedded (SUR sampled) GF sample is superimposed with a stereological test system combining 35 cycloids and 70 points. The short side of the rectangular frame of the system and therewith the minor axis of the cycloids is aligned parallel to the orientation of the vertical axis of the VUR GF section (VA, indicated by the arrow on the left). All points hitting GF section profiles (P_(GF)_, indicated in green) are counted, as well as all intersections of cycloids with the epithelial surface of SL section profiles (I_(SL)_, encircled in red). The SL surface area density in the GF is calculated from the sum of intersections (∑I_(SL)_) and points (∑P_(GF)_), counted in all examined test fields of all sections of all samples per case, using **Eq 6**. In the presented example, the length of one cycloid (l) = 1/10 of the frame width [62], the test curve length in general is calculated from cycloid arch height h as: l = 2 x h [104]. GMA/MMA. HE. Bar = 50 μm.

The surface area density of the secondary lamellae in the gill filaments (S_V(SL/GF)_) is determined in VUR sections of GMA/MMA-embedded SUR gill filament (GF) samples (**Sections 9** and **10**, **Figs 1** and **5**). S_V(SL/GF)_ is estimated using a stereological test system combining test points and cycloids (*i*.*e*., test lines that interact isotropically with surface section profiles in VUR sections) [24,62,104], in which a defined length of cycloid arches (at a given factor of magnification) is associated with a known number of test points. A detailed general description of the theoretical basis of surface area estimation by cycloid test systems in VUR sections is provided in Baddeley et al. [62] or Howard and Reed [25], copy templates of point/cycloid test systems with indicated p/l quotients are provided in the supplementary data of Howard and Reed [25]. **Fig 13** illustrates the practical application of the method for determination of S_V(SL/GF)_ in trout gill VUR samples. At 100x microscopic magnification, microscopic test fields are SUR sampled within the VUR sections by meander sampling (**Fig 12E**) or with appropriate stereology software tools. The point/cycloid test system is superimposed to the sampled test fields and aligned to the VUR section image so that the vertical axis (*i*.*e*., the minor axis) of the cycloids (**Fig 13**) is parallel to the vertical axis of the VUR section of the GF sample. In each test field, the number of intersections between the epithelial surface of the secondary lamellae and the cycloid test lines (I_(SL)_) is counted, as well as the number of points hitting gill filament section profiles (P_(GF)_). Additionally, points hitting profiles of secondary gill lamellae in the same test fields are counted (P_(SL)_), if V_V(SL/GF)_ is to be determined from the quotient of ∑P_(SL)_/∑P_(GF)_ (refer to **Section 12**).

S_V(SL/GF)_ is calculated from the cumulative number of intersections (∑I_(SL)_) and points (∑P_(GF)_) counted in all examined test fields in all sections of all samples per case in given magnification (**Eq 6**) [62]. S_V(SL/GF)_ is then corrected for the extent of GMA/MMA-embedding-related tissue shrinkage, using the linear tissue shrinkage factor (f_s_) for GMA/MMA-embedded gill filaments (**Section 11**). The total surface area of the secondary gill lamellae in the gill filaments (S_(SL,GF)_) is calculated as the product of S_V(SL/GF)_ and the gill filament volume (V_(GF)_) (**Eq 7**), which is calculated directly via submersion method (**Section 8**). Using the described methodological approach, we determined a (shrinkage-corrected) S_V(SL/GF)_ of 333.53 cm^2^/cm^3^ and, correspondingly, a S_(SL,GF)_ of 947.24 cm^2^ in a healthy rainbow trout of ~1300 g body weight (**S1F Experimental data**).

**Eq 6**. **Surface area density of the secondary lamellae in the gill filaments**.

$$S_{V(SL/GF)}=(2\times (p/l)\times \sum I_{\left(SL\right)}/\sum P_{\left(GF\right)})\times f_{s}$$

**S**_**V(SL/GF)**_ Surface area density of the secondary lamellae (SL) in the gill filaments (GF), corrected for embedding-related shrinkage

**p/l** Ratio of test point number to cycloid arch length at level of the tissue

**∑I**_**(SL)**_**/∑P**_**(GF)**_ Quotient of the total number of intersections between the epithelial surface of the SL and cycloid arches in all analyzed sections per case and the total number of points hitting section profiles of GF

**f**_**s**_ Linear tissue shrinkage factor for GMA/MMA-embedded GF (0.87)

**Eq 7**. **Total surface area of the secondary lamellae in the gill filaments**.

$$S_{\left(SL,GF\right)}=S_{V(SL/GF)}\times V_{\left(GF\right)}$$

**S**_**(SL,GF)**_ Surface area of the secondary lamellae (SL) in the gill filaments (GF)

**S**_**V(SL/GF)**_ Surface area density of the SL in the GF, corrected for embedding-related shrinkage

**V**_**(GF)**_ Total volume of the GF sample

### 14. Estimation of the total number, the total volume and the mean volume of epithelial cells in the secondary gill lamellae

Unbiased estimates of the total number of epithelial cells (EC) in the secondary lamellae (SL) (N_(EC,SL)_) and of the mean cellular volume of SL-EC (${\bar{v}}_{\left(\mathrm{EC},\mathrm{SL}\right)}$) are determinant measures for the characterization and differentiation of gill epithelial alteration patterns such as cell loss, atrophy, hypertrophy, and hyperplasia. The numerical volume density of epithelial cells in the secondary lamellae (N_V(EC/SL)_) is estimated using IUR sections of SUR sampled, Epon-embedded gill filament (GF) samples (refer to **Figs 1** and **5**, **Sections 9** and **10**). For unbiased estimation of numerical volume densities of cells in their reference tissue compartment, the physical disector method is applied, combined with systematic point counting [24,25,65,107]. The physical disector represents a 3-D stereological test system, used for unbiased counting and sampling of particles, independent of the size, shape, orientation and distribution of the particles within their reference compartment. A physical disector consists of two parallel, corresponding histological sections (*i*.*e*., a “reference” section and a “look-up” section) with a known distance (disector height) between the sections [60,65,107], thus defining a known volume of the tissue between the two sections.

**Fig 14:**
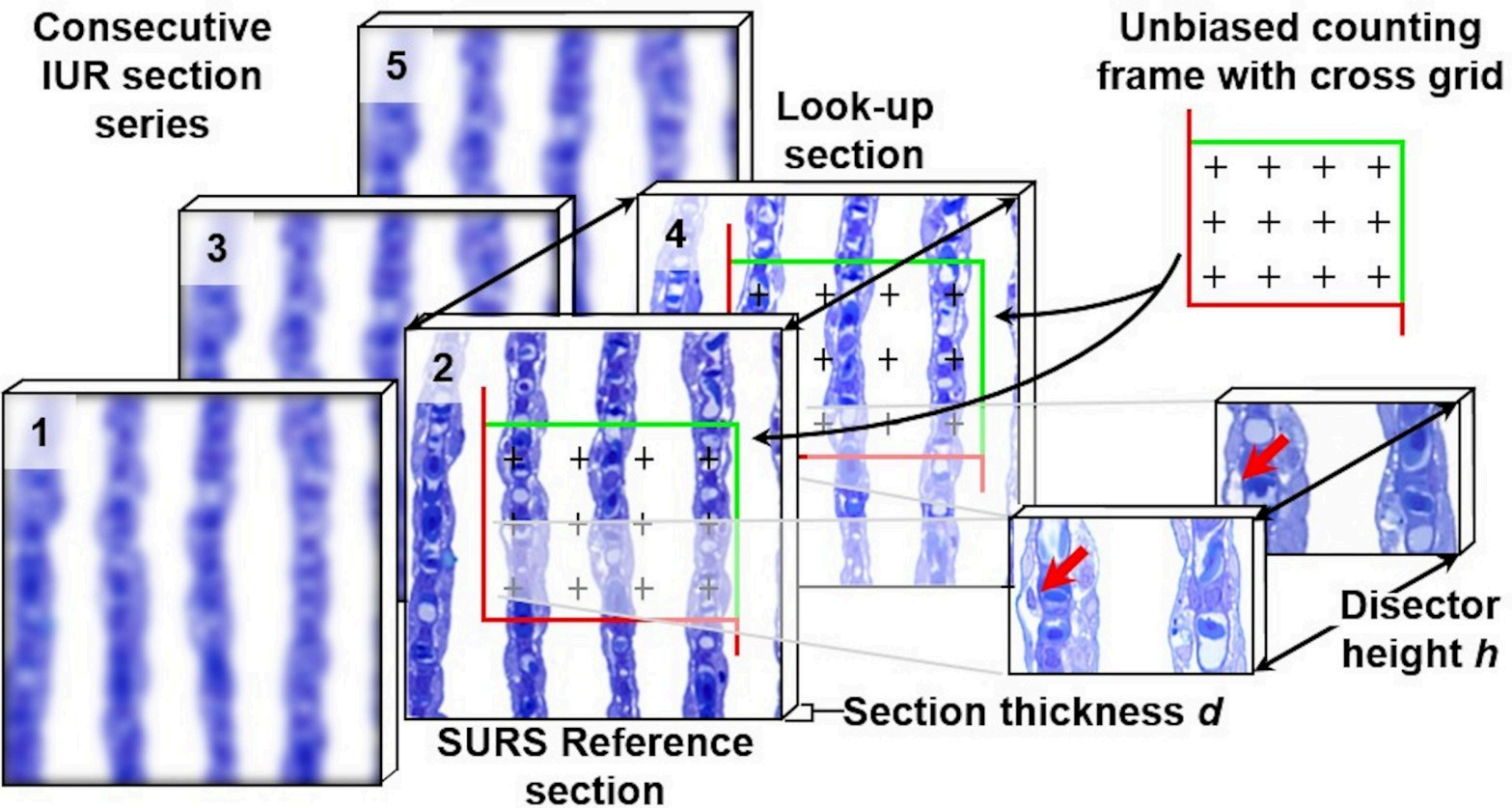
Estimation of the numerical volume density of epithelial cells in the secondary gill lamellae. The number of epithelial cells (EC) per volume unit of secondary lamellae (SL) is estimated, using the physical disector as a 3-D stereological test system for unbiased counting of particles. A physical disector consists of two parallel histological sections (a reference section and a look-up section) with a defined distance (disector height, h). The reference section is SUR sampled from a series of technically impeccable, parallel, consecutive sections with a defined nominal section thickness. The factual physical thickness of the sections (d) defines the disector height. The present example shows corresponding fields of view in a series of 5 consecutive, toluidine blue stained, semithin IUR GF sections with a nominal thickness of 0.5 μm (the examined fields of view in the section are SUR sampled at the given factor of magnification). From the series of five sections, the second section is SUR sampled as reference section. The fourth section (*i*.*e*., with a distance of h = 2 x d = 2 x 0.5 μm = 1 μm) is defined as look-up section. Corresponding section areas in the reference- and the look-up section are overlaid with an unbiased counting frame [110] of known area and a cross grid of equally spaced test points. The volume of the reference compartment defined by the disector probe, *i*.*e*., the volume of SL within the 3-D space defined by both sections of the disector, is given by the disector height and the mean area of the SL section profile(s) (*i*.*e*., the reference compartment) present in the reference- and the look-up section. The section area of SL within the area of the unbiased counting frame is determined by point counting: the number of crosses hitting SL section profiles is counted and multiplied by the area associated with a single point/cross of the grid (*i*.*e*., the quotient of the number of crosses in the counting frame and the area of the counting frame). SL-EC nuclei that are sectioned in the reference section, but not in the look-up section are counted (Q^-^), using the unbiased counting frame (particle sections are only counted if they are completely located within the unbiased counting frame or if they touch one of the “acceptance” (green) border lines. Any particle section profiles touching an “exclusion” (red) line are not counted) [25]. In the presented example, a SL-EC nucleus section profile that is present in the reference section, but absent in the look-up section, is indicated by red arrows. The numerical volume density of the SL-EC is then calculated from the EC number counted in all sections of all samples per case in all analyzed disectors and the cumulative reference compartment (SL) volume in all analyzed disectors (**Eq 8**).

The reference- and look-up sections are usually taken from a series of consecutive sections, sectioned with a defined nominal section thickness (d). For determination of N_V(EC/SL)_, it is recommendable to prepare a series of at least 7 consecutive semithin sections (per sample) [108,109] with a nominal section thickness of 0.5 μm. The mean section profile area of the reference compartment (SL) that is present in the reference- and the look-up section and the disector height define the 3-D reference compartment volume in which the particles of interest (SL-EC) are counted. The disector height (and therefore also the disector volume) depends on the number of sections of the section series located between the reference- and the look-up section and on the factual individual section thickness [25,80]. For accurate analysis results, the nominal section thickness (set on the microtome) therefore needs to be controlled by determination of the factual physical section thickness. The factual thickness of sections of plastic-resin-embedded samples can be expeditiously determined by contact-free spectral reflectance measurement or, more elaborately, by electron microscopy of ultrathin sections of orthogonally re-embedded sections [80]. For counting of cells with the physical disector, the reference- and the look-up section are compared. Cells are counted, if their cell nuclei are sectioned by the reference-, but not by the look-up section. Usually, a disector height of approximately ⅓^rd^ of the mean minimal orthogonal linear projection of the cell nuclei (*i*.*e*., their mean minimal diameter) is chosen, because small nuclei that are completely located between the reference- and the look-up section would be unintentionally overseen during the cell (nuclei) counting process [25,107]. For determination of the appropriate disector height, the mean minimal diameter of a sufficient number (~50) of nuclear cross section profiles of the target cells is determined in the reference section, using appropriate morphometry software tools. To warrant the unbiasedness of the analysis, the reference section is randomly sampled from the section series, and the look-up section is selected according to the previously defined disector height. For estimation of N_V(EC/SL)_, a disector height of 1 μm is recommended (*i*.*e*., in a section series of seven 0.5 μm thick sections (N°1–7), three possible disector section pairs can be sampled, each with one section between the randomly sampled reference- and the look-up section (N°2 and 4, N°3 and 5, or N°4 and 6)). Within the reference section, the examined fields of view are SUR sampled at the given factor of magnification (and photographed). A microscopic magnification of 200x or 400x is recommendable for disector analysis of N_V(EC/SL)_. The corresponding fields of view within the look-up section are then localized and photographed as well. The images of corresponding fields of view in the reference- and the look-up section are appropriately aligned and superimposed with unbiased counting frames of known area [25,110]. For images representing section areas of approximately 500 μm x 300 μm—230 μm x 150 μm, counting frame areas between 200 μm x 100 μm and 150 μm x 70 μm are recommended. Following the rules for sampling and counting of particles with the unbiased counting frame (**Fig 14**), the number of nuclear profiles of the cell type of interest (SL-EC) present in the reference section and absent in the look-up section is counted (Q^-^) [107]. The section profile area of the SL reference compartment within the unbiased counting frame is determined by point counting (**Sections 11** and **12**) [24,26]. The SL volume within the disector volume is then determined from the quotient of the mean SL section profile area within the unbiased counting frames in the reference- and the look-up section and the (known) area of the unbiased counting frame. N_V(EC/SL)_ is calculated from the number of EC (Q^-^) counted in all analyzed disectors per case and the cumulative volume of the SL in these disectors (**Eq 8**). To obtain reliable numerical volume estimates, at least 50 nuclei (*i*.*e*., Q^-^, particles, cells) should be counted per case.

The efficiency of the described approach is doubled, if cell section profiles that are present in the look-up section and absent in the reference section are counted as well (*i*.*e*., interchanging the roles of the reference- and the look-up section)–if done so, it must be considered that this approach technically implies an independent analysis of two separate disectors. The total number of EC in the SL (N_(EC,SL)_) is calculated from the N_V(EC/SL)_ and the volume of the SL in the GF (V_(SL,GF)_). N_V(EC/SL)_ is corrected for the embedding-related shrinkage of GF samples, using the linear tissue shrinkage factor (f_s_) for Epon-embedded tissue (0.95) (**Eq 9**) [111].

The identical images can be used to simultaneously determine the volume density of the EC in the SL (V_V(EC/SL)_) by point counting. For estimation of V_V(EC/SL)_, it is sufficient to analyze either the reference- or the look-up section of a disector. The applicable size of the cross grid, *i*.*e*., the number of points per counting frame, is determined according to the number of examined disectors and the V_V(EC/SL)_ [26]. With a V_V(EC/SL)_ of ~0.50 and an average number of 10–15 analyzed disectors within the section series of 5 samples per case (**S1G Experimental data**), a grid of 10x10 points per counting frame is generally sufficient to determine V_V(EC/SL)_ with an expected relative error probability of ~5% of mean V_V(EC/SL)_ (**S1H Experimental data**).

The total volume of SL-EC (V_(EC,SL)_) in the SL is calculated from the estimates of V_V(EC/SL)_ and V_(SL,GF)_ (**Eqs 10** and **11**). The mean cellular volume of SL-EC (${\bar{v}}_{\left(\mathrm{EC},\mathrm{SL}\right)}$) results from the quotient of V_V(EC/SL)_ and N_V(EC/SL)_, according to **Eq 12**. There are alternative methods for the counting of particles and the estimation of the mean particle volume on a single thick section: the optical disector, where consecutive, parallel IUR sections are generated optically and not physically, and the nucleator [65]. In previously published studies on fish, the nucleator was used in analyses of the liver [112] or gonads [113], in an ecotoxicological study examining the toxic effects of copper, the nucleator was used for estimation of the mean cellular volumes in the gills [21].

**Eq 8**. **Numerical volume density of epithelial cells in the secondary lamellae**.

$$N_{V(EC/SL)}=(\sum Q_{\left(EC\right)}^{-}/(h\times \sum A_{\left(SL\right)}))\times f_{s}^{3}$$

**N**_**V(EC/SL)**_ Numerical volume density of epithelial cells (EC) in the secondary lamellae (SL), corrected for embedding-related tissue shrinkage

**∑Q**^**-**^_**(EC)**_ Cumulative number of all counted EC nuclei in all analyzed disectors per case

**h** Disector height (*i*.*e*., the distance between the reference and the look-up section)

**∑A**_**(SL)**_ Cumulative area of SL section profiles in all disectors per case (for each analyzed disector, the mean area of the SL section profiles in the reference- and the look-up section is determined)

**f**_**s**_ Linear tissue shrinkage factor for Epon-embedded tissue (0.95) [111]

**Eq 9**. **Total number of epithelial cells in the secondary lamellae**.

$$N_{\left(EC,SL\right)}=N_{V(EC/SL)}\times V_{\left(SL,GF\right)}$$

**N**_**(EC,SL)**_ Total number of epithelial cells (EC) in the secondary lamellae (SL)

**N**_**V(EC/SL)**_ Numerical volume density of EC in the SL, corrected for embedding-related tissue shrinkage

**V**_**(SL,GF)**_ Total volume of the SL in the gill filaments (GF)

**Eq 10**. **Volume density of epithelial cells in the secondary lamellae**.

$$V_{V(EC/SL)}=A_{A(EC/SL)}=\sum A_{\left(EC\right)}/\sum A_{\left(SL\right)}=\sum P_{\left(EC\right)}/\sum P_{\left(SL\right)}=P_{P(EC/SL)}$$

**V**_**V(EC/SL)**_ Volume density of the epithelial cells (EC) in the secondary lamellae (SL)

**A**_**A(EC/SL)**_ Area density of EC in the SL

**∑A**_**(EC)**_**/∑A**_**(SL)**_ Quotient of the cumulative section area of EC in all examined reference compartment sections per case and the cumulative section area of SL in the same sections

**∑P**_**(EC)**_**/∑P**_**(SL)**_ Quotient of the total number of points hitting section profiles of EC in all examined sections per case and the total number of points hitting SL in the same sections

**P**_**P(EC/SL)**_ Point density of EC in the SL

**Eq 11**. **Total volume of epithelial cells in the secondary lamellae**.

$$V_{\left(EC,SL\right)}=V_{V(EC/SL)}\times V_{\left(SL,GF\right)}$$

**V**_**(EC,SL)**_ Total volume of the epithelial cells (EC) in the secondary lamellae (SL)

**V**_**V(EC/SL)**_ Volume density of EC in the SL

**V**_**(SL,GF)**_ Total volume of the SL in the gill filaments (GF)

**Eq 12**. **Mean cellular volume of epithelial cells in the secondary lamellae**.

$${\bar{v}}_{\left(EC,SL\right)}=V_{V(EC/SL)}/N_{V(EC/SL)}$$

${\bar{v}}_{\left(\mathrm{EC},\mathrm{SL}\right)}$ Mean cellular volume of the epithelia cells (EC) in the secondary lamellae (SL)

**V**_**V(EC/SL)**_ Volume density of EC in the SL

**N**_**V(EC/SL)**_ Numerical volume density of EC in the SL, corrected for embedding-related tissue shrinkage

### 15. Determination of the true harmonic mean of the diffusion barrier thickness in the secondary gill lamellae

Accurate estimates of the (oxygen) diffusion distance are essential for identification, quantification, comparison and evaluation of alterations of the diffusion resistance across the diffusion barrier in the secondary gill lamellae due to *e*.*g*., epithelial lifting, cell hypertrophy, hyperplasia [3] and atrophy, (experimentally) induced by aquatic pollutants/test substances. The thicknesses of biological barriers, such as glomerular basement membranes [28,111,114] or pulmonary oxygen diffusion distances [29,103] in mammals, or the thickness of the gill diffusion barrier [38,40], are unbiasedly estimated by their true harmonic mean thickness (T_h_), T_h_ is determined from orthogonal intercepts [28,103], as described below.

The true harmonic mean of the diffusion barrier thickness (T_h(DB)_) is determined in transmission electron microscopic (TEM) images captured in ultrathin IUR sections of SUR sampled, Epon-embedded gill filament (GF) specimens (refer to **Fig 5**, **Sections 9** and **10**), using a logarithmic ruler (**S9 Fig**) [28]. If TEM analysis is not available, the method can (as an exception) be applied using light microscopic (LM) images of semithin sections of the respective samples, acquired at high magnifications (**S10 Fig**).

Within the ultra-, respectively semithin IUR sections, fields of view containing SL section profiles are SUR sampled at a given factor of magnification (for TEM analysis of the T_h(DB)_, a magnification factor of 8000-20000x is recommended, and 1000x (oil immersion) for LM-analysis, respectively), photographed, and printed (with plotted size rulers). The factual final magnification of the printed images (M) is determined. For sampling of the diffusion barrier thickness measurement sites, the section images are randomly overlaid with a quadratic line grid printed on a plastic transparency. For TEM images printed in a final magnification of approximately 20000x, a grid size of 1.5 cm x 1.5 cm is recommendable.

**Fig 15:**
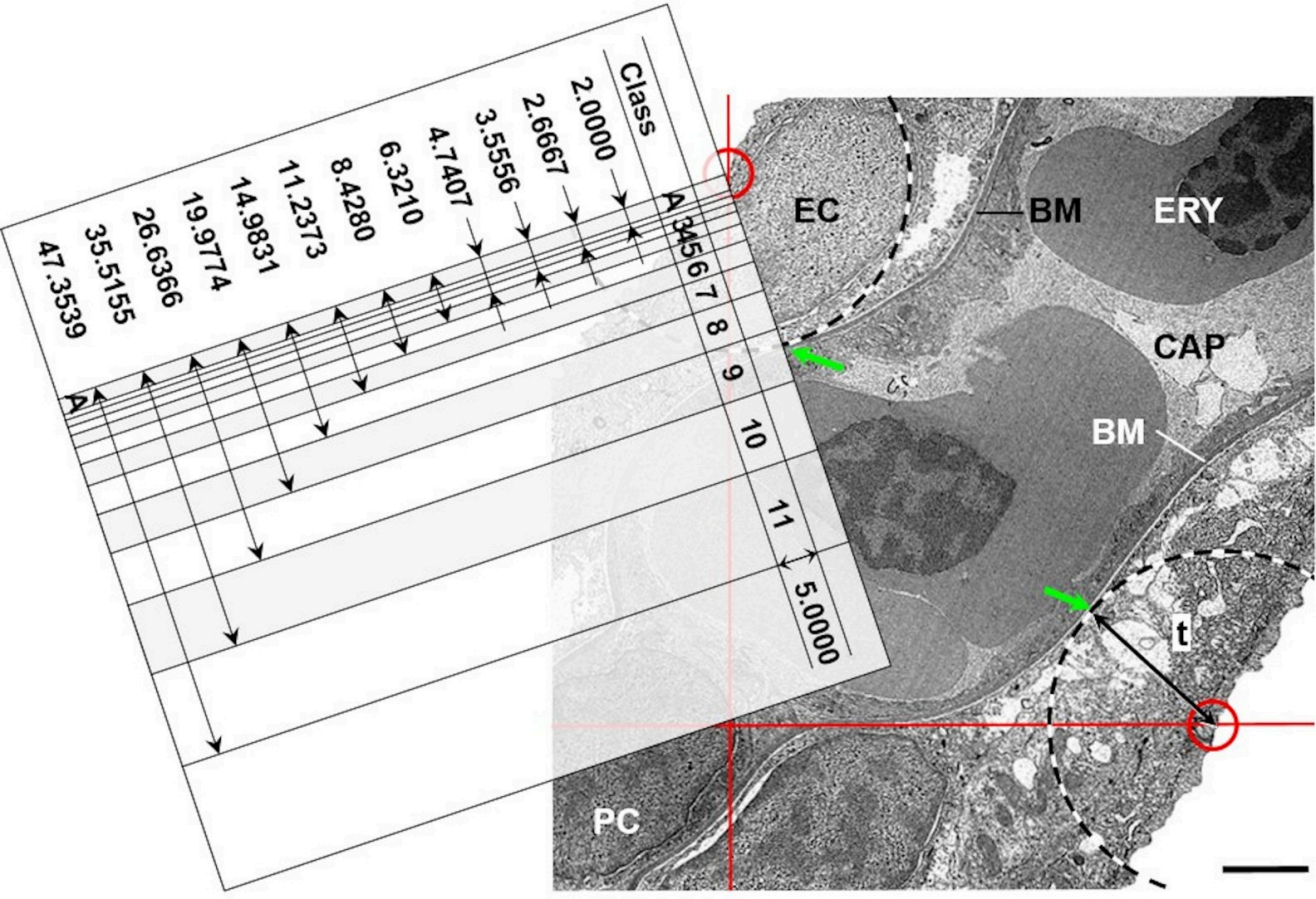
Determination of the true harmonic mean of the diffusion barrier thickness in secondary gill lamellae. A printed transmission electron microscopic (TEM) image of a SUR sampled field of view of an IUR section of a secondary lamella (SL) is superimposed with a test grid of lines (red). The transections of the gridlines with the epithelial SL surface are marked by red circles. At these locations, the shortest distance (t, double arrow) between the epithelial surface of the SL and the inner surface of the blood space (green arrows) is determined (dashed circles). Along these lines, the diffusion barrier thickness is measured, using a superimposed logarithmic ruler. In the shown example, the measured distance falls in class 9. T_h(DB)_ is calculated from the number of measurements and the corresponding classes (**Eq 13**), detailed illustration is given in Hirose et al. [115]. Epon. TEM. Bar = 2 μm.

Diffusion barrier thickness measurements are conducted at the transection points of grid lines and secondary lamellae (SL) surface section lines (**Figs 15** and **S10**). A logarithmic ruler printed on a plastic transparency is used to measure the diffusion distance along the shortest distance between a transection point and the inner surface of the SL vascular space. This distance is not measured linearly, but in terms of ruler “classes” (A, 1–11). Each ruler class is defined by a lower and upper limit measured from the origin of the ruler and a defined midpoint. In a given study, the ruler dimensions are adapted to the thickness of the diffusion distances in the printed TEM/LM images at a given final magnification, so that none of the measurements falls within the initial division “A” [28,111]. The dimensions of the ruler classes are provided in **S1 Table**, as well as ruler copy templates suitable for analysis of apparent diffusion barrier section profile distances on IUR SL sections (**S9 Fig**).

T_h_ is calculated from the number of measurements (observations) made in each ruler class (with defined class-midpoints) per case (**S1I Experimental data**), and the final magnification (M) of the printed IUR section images (**Eq 13**) [111,115,116]. A detailed calculation example is provided in Hirose et al. [115].

**Eq 13**. **True harmonic mean of the diffusion barrier thickness in the secondary lamellae**.

$$T_{h(DB)}=(8/3\pi )\times (10^{6}/M)\times {\bar{l}}_{h(DB)}$$

**T**_**h(DB)**_ True harmonic mean of the diffusion barrier (DB) thickness

**8/3π** Correction factor for oblique sectioning

**M** Final print magnification

**Ī**_**h(DB)**_ Apparent harmonic mean thickness of the diffusion barrier

(Number of observations/(Midpoints x Number of observations))

### 16. Application of laser light sheet fluorescence microscopy (LSFM) of optically cleared trout gill samples in quantitative histomorphological analyses

#### 16.1 LSFM of optically cleared samples and its application for quantitative morphological analyses of trout gills

Determination of quantitative (3-D) morphological (gill) parameters using the “classical” stereological approaches featured above is essentially based on analysis of 2-D histological sections. The necessity to examine 2-D histological sections to acquire information about the 3-D organ/tissue morphology eventually lies in the fact(s) that most tissues are non-transparent (therefore thin, light-permeable sections are needed to visualize the structures of interest) and that the structures of interest are often too small to be recognized by the naked eye (implying the need of optical magnification).

**Fig 16:**
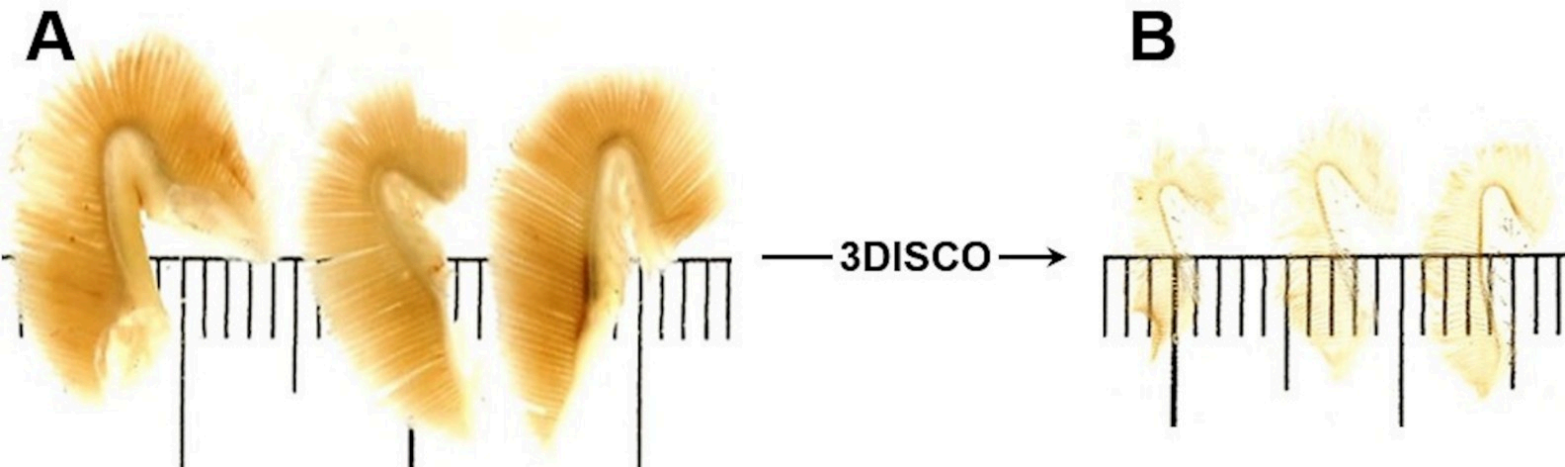
Optical clearing of rainbow trout gills. **A.** Formalin-fixed (non-transparent) rainbow trout gills, placed on a mm ruler. **B.** The identical gills after optical tissue clearing with the 3DISCO protocol. Note the transparency and the shrinkage of the cleared gills. Note that the images in A and B show intact gills (with gill arches). For LSFM-based quantitative morphological analyses, the gill arches are removed from the gills and the gill filaments are cleared after determination of their weight or volume.

In the recent years, LSFM of optically cleared (*i*.*e*., transparent) specimens has emerged as an innovative imaging technology for direct and fast microscopic examination of 3-D samples, elegantly bypassing the necessity of preparation of histological sections [50–53,117]. LSFM of optically cleared samples enables the 3-D representation of complex organ/tissue architectures (**S1 Fig**), and also holds a great potential for quantitative morphological analyses [56–58]. By now, a substantial number of different tissue clearing methods have been developed for generation of optically transparent samples of diverse organs and tissues, as reviewed in Feuchtinger et al. [52], Hong et al. [118], or Ueda et al. [119]. Basically, optical clearing of a tissue is achieved by treatment with diverse chemical compounds that remove and/or alter light diffracting tissue components, such as lipids and cell membranes, thereby adjusting the refractive index of the tissue to that of the surrounding medium [52,120]. For optical clearing of trout gill samples for subsequent LSFM analysis of quantitative morphological parameters, application of the 3DISCO (3-dimensional imaging of solvent cleared organs) (“brain”-) protocol [51] is recommendable, allowing for a fast (overnight) and cost-efficient processing of gill samples, using only few chemical compounds (tetrahydrofuran (THF), dichloromethane (DCM), dibenzylether (DBE) or benzyl alcohol and benzyl benzoate (BABB)). 3DISCO-cleared (gill) samples display a well retained shape, an adequately firm consistency, and a reproducible extent of uniform clearing-related volume-shrinkage of approximately 50% (**Fig 16**) (**S1J Experimental data**).

The 3DISCO protocol used for clearing of gill samples (**S2 Table**) is a slightly altered version of the “Brain (long protocol)”-version of the 3DISCO protocol originally published by Ertürk et al. [51].

**Fig 17:**
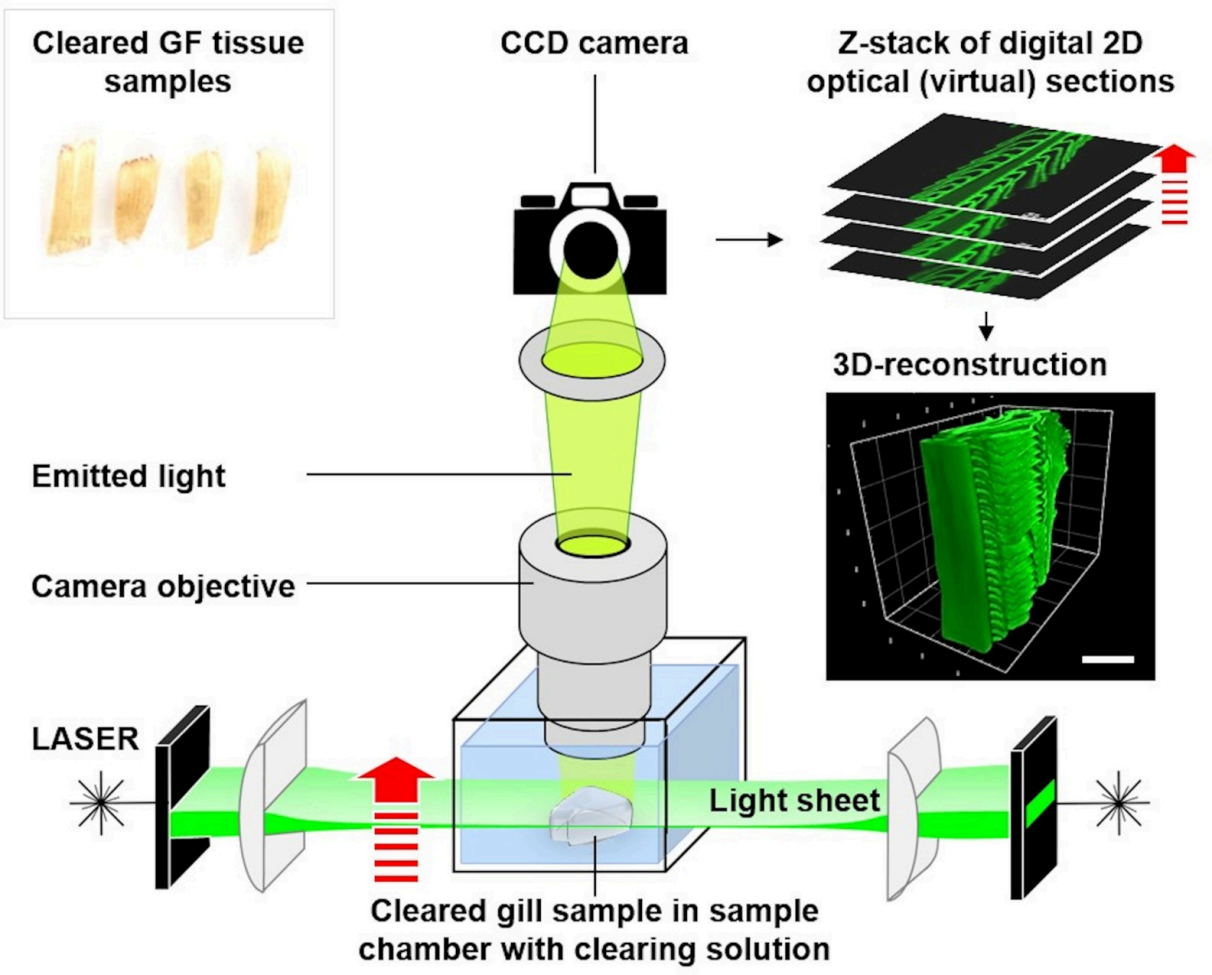
Laser light sheet fluorescence microscopy (LSFM) of an optically cleared gill filament sample. The cleared GF sample in the sample chamber is stepwise illuminated by thin laser light sheets of defined wavelength ranges. Here, autofluorescence signals emitted by GF structures excited by the laser light energy are detected by a digital charge-coupled device (CCD) camera, resulting in a 2-D fluorescent image of the illuminated focus plane in the sample. The sample is gradually moved through the laser light sheet(s) along its vertical axis (red arrow), resulting in the acquisition of a z-stack of serial fluorescence images, used to compute a 3-D reconstruction of the sample. In the present example, autofluorescence images of a 3DISCO-cleared GF sample acquired at 520/40 nm (excitation range) (ex) and 585/40 nm (emission range) (em) are shown. Bar = 200 μm.

Cleared (transparent) gill filament (GF) samples of a size of up to approximately 3 cm x 3 cm x 2 cm (length x width x height) can directly be analyzed *in toto* by LSFM, the principle of LSFM of cleared samples is illustrated in **Fig 17**. The cleared GF sample is placed into the ultramicroscope sample chamber which is filled with an appropriate clearing solution (here: BABB). A sheet (*i*.*e*., a few μm thin plane) of laser light of adjustable wavelength is sent through the transparent sample. Fluorescence signals emitted by organ/tissue structures after excitation by the laser light energy are detected perpendicular to the illumination axis by a digital camera, resulting in a 2-D fluorescent image of the illuminated focus plane in the sample (*i*.*e*., a virtual digital optical section of the illuminated plane). For a uniform illumination of the entire width of a sample, two precisely aligned light sheets are used to simultaneously illuminate the cleared sample from two opposite sides. The fluorescence signals detectable at specific laser wavelength ranges either originate from the (natural) autofluorescence of different organ/tissue components, from *in-* or *ex-vivo* administered fluorescence-labeled tracer substances such as antibodies or lectins [57,121–123], or from the transgene-expression of specific fluorescent reporter molecules, such as EGFP (enhanced green fluorescent protein) or mCherry [124,125]. The sample is moved through the laser light sheet along its vertical axis, with step sizes as small as ≥5 μm, resulting in the acquisition of a z-stack of serial fluorescence images of parallel (virtual) optical sections of the sample. Subsequently, a virtual digital 3-D reconstruction of the sample that can be freely rotated and sectioned is computed from the acquired z-stack of fluorescence images (**S1 Fig**, **S1 Video**), using appropriate image analysis software.

In the present work, an UltraMicroscope II (LaVision BioTec GmbH, Germany) equipped with a SuperK EXTREME EXW12 white laser (NTK Photonics, Germany) and a 2x objective lens (Olympus MVPLAPO 2X/0.5 NA) combined with an Olympus MVX-10 zoom body (Olympus, Germany) was used for LSFM analyses of 3DISCO-cleared gill filament samples. Z-stacks of fluorescence images of 5 μm optical thickness were acquired at 520/40 nm (excitation range) (ex) and 585/40 nm (emission range) (em) for detection of autofluorescence. 3-D images were computed, using ImSpector Pro^64^ (vers. 5.1.328, LaVision Biotec GmbH, Germany) and arivis Vision4D (vers. 3.0, arivis, Germany) software tools.

LSFM-based quantitative morphological analyses of cleared (gill filament) samples basically follow the same sampling- and analysis principles as the “classical” section-based stereological analysis approaches, solely using fluorescence images of 2-D optical (virtual) sections instead of factual (physical) histological slides. The advantage of using LSFM for quantitative morphological analyses, as compared to “classical” stereological analysis approaches, is indeed simply based on the unmatched speed and simplicity of generation of (virtual) optical sections of defined orientations. Moreover, since the cleared samples are not physically sectioned during LSFM analysis, (virtual) optical sections of any orientation (transverse, sagittal, horizontal, VUR, IUR) can be generated successively from the identical specimen. Finally, LSFM-based analyses of the relevant morphological parameters V_V(SL/GF)_ and S_V(SL/GF)_ featured in the present guide can be adequately performed using the natural autofluorescence of 3DISCO-cleared gills, *i*.*e*., no fluorescent labeling of the samples is necessary.

#### 16.2 LSFM-based determination of volume- and surface area densities of secondary gill lamellae in the gill filaments

**Fig 18:**
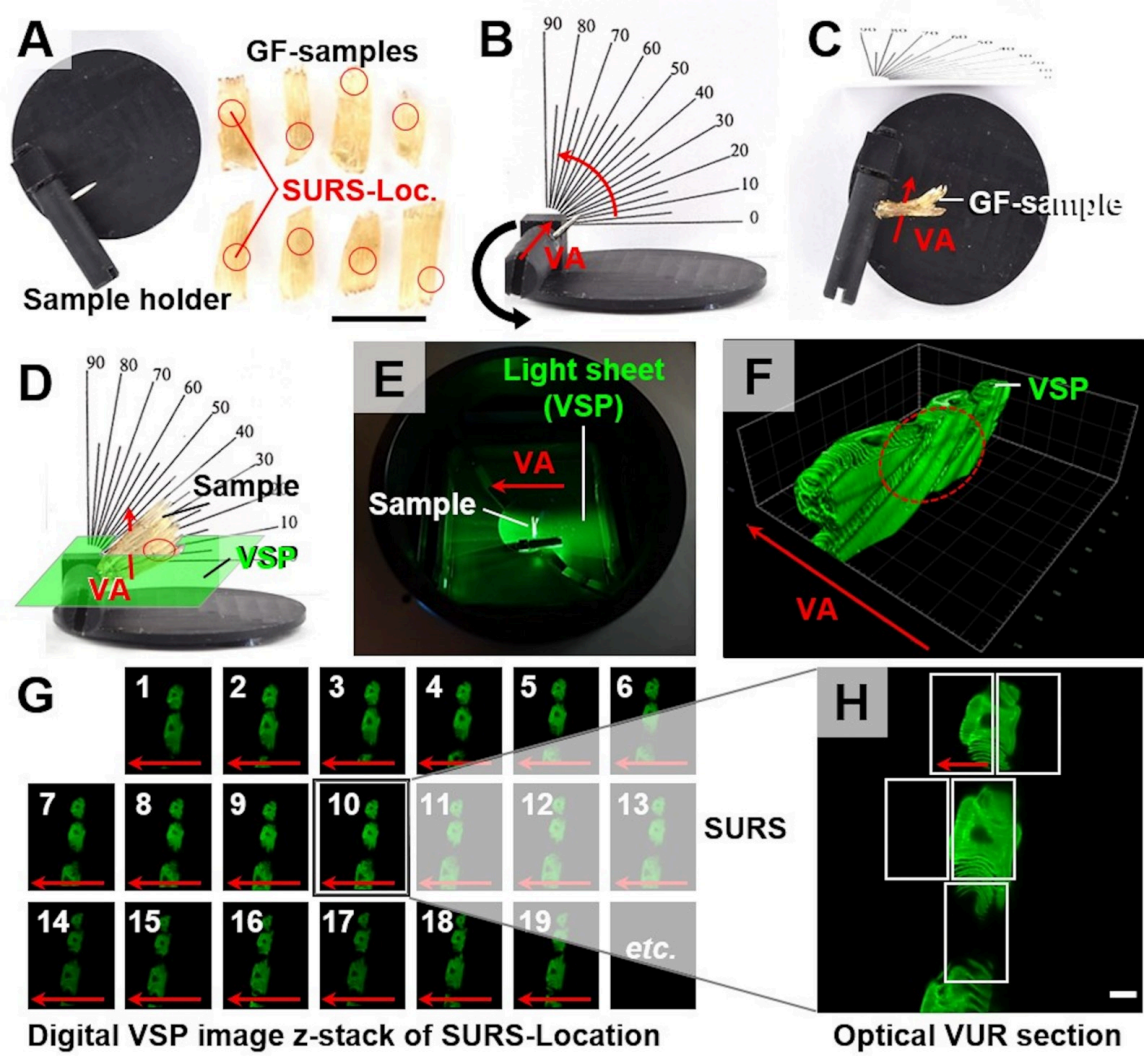
Generation of virtual optical VUR sections of cleared gill filament (GF) samples by LSFM. **A.** Left image side: top view of a sample holder with a rotatable axis equipped with a needle for sample attachment. Right image side: 8 stripe-shaped samples of optically cleared GF containing the SUR sampled locations (**SURS-Loc.**, indicated by red circles). **B-E.** For generation of (virtual, optical) VUR sections, the GF sample is pinned to the rotatable axis (**B**) of the sample holder, so that the opercular side of the sample is oriented perpendicular to the (user-defined, virtual) vertical axis (**VA**, indicated by a red arrow). The sample is then rotated in a defined rotation interval (compare to **Fig 10**). In **D**, a rotation angle of ~40° is shown. A green schematic plane indicates the orientation of a corresponding vertical section plane (**VSP**), relative to the VA and the sample. The sample holder with the attached sample is then transferred into the sample chamber of the LSF-microscope (**E**), maintaining the orientation of the sample to the (horizontal) plane of the laser light sheet. **F, G.** A z-stack series of digital autofluorescence images (*i*.*e*., virtual vertical section planes, parallel to VA) of the SURS-Loc. in the sample is acquired at an appropriate magnification (**G**). In **F**, the 3-D reconstruction of the region of the GF sample that contains the SURS-Loc. is shown. **G, H.** Depending on the applied magnification factor and the examined parameter, one to three images are (systematically) randomly sampled from the z-stack series of digital virtual optical VSP images of the SURS-Loc. (*e*.*g*., N°10) of each GF sample for subsequent analysis of S_V(SL/GF)_ (and V_V(SL/GF)_). For estimation of S_V(SL/GF)_, inclusion of a calibrated size ruler and indication of VA in the VUR image are mandatory. Note that the orientation of the VA of the sample is always recognizable (in the cleared sample, the 3-D reconstruction, the (virtual) VUR section images, and in the SUR sampled fields of view within these VUR sections). Bar = 1 cm in A and = 200 μm in H.

For determination of volume and surface area densities in LSFM virtual optical sections of cleared gill filament (GF) samples, the generally applicable sampling designs and stereological probes are the same as for the “classical” quantitative stereological analysis approaches described above. For practical reasons, the gill filaments of the four (formalin-fixed) gill arches of one side (right or left) are optically cleared *in toto* after determination of the gill filament volume by submersion volumetry/weighing. Per case, 8 GF locations are SUR sampled from the cleared gill filaments, principally as described above (**Section 9**). For subsequent handling and analysis of the samples, it is advantageous to excise gill filament stripes (**Fig 18A**) containing the SUR sampled GF location, rather than using a biopsy punch to excise cylindrical tissue specimens. The SUR sampled cleared GF-stripes are then LSFM-imaged for acquisition of digital (virtual) optical VUR autofluorescence section images for subsequent analysis of V_V(SL/GF)_ and S_V(SL/GF)_, as shown in **Fig 18**. The vertical uniform randomization of the (virtual) optical section plane orientation through the cleared SUR GF samples is achieved by systematically rotating the samples around a fixed vertical axis (for practical reasons, a line perpendicular to the sagittal gill plane is defined as vertical axis) in a predefined interval (*i*.*e*., 22.5° for 8 samples, with the first sample being randomly rotated within the interval of 0°-22.5°, as described in **Section 10**). The SUR sampled cleared GF specimen is pinned onto a needle attached to a rotatable axis mounted on a modified LSFM-sample holder (**Fig 18A**). The position of the sample is aligned so that the vertical axis of the sample is parallel to the rotatable axis of the sample holder. The angle of the VUR section plane relative to the sample is set by rotation of the axis in the predefined interval. The sample holder with the attached sample is then transferred into the sample chamber of the LSF-microscope and imaged at an appropriate wavelength for autofluorescence detection (520/40 nm (ex) and 585/40 nm (em)). The featured approach prevents non-true-to-scale optical distortions of optical 2-D VUR sections that might probably occur in VUR sections virtually computed from the 3-D reconstruction of the specimen, as illustrated in **S11 Fig**. From the acquired series of optical (virtual) VUR section images from each sample, one to three image(s) per SUR sampled GF location are (systematically) randomly sampled for subsequent stereological analysis (**Fig 18G and 18H**).

**Fig 19:**
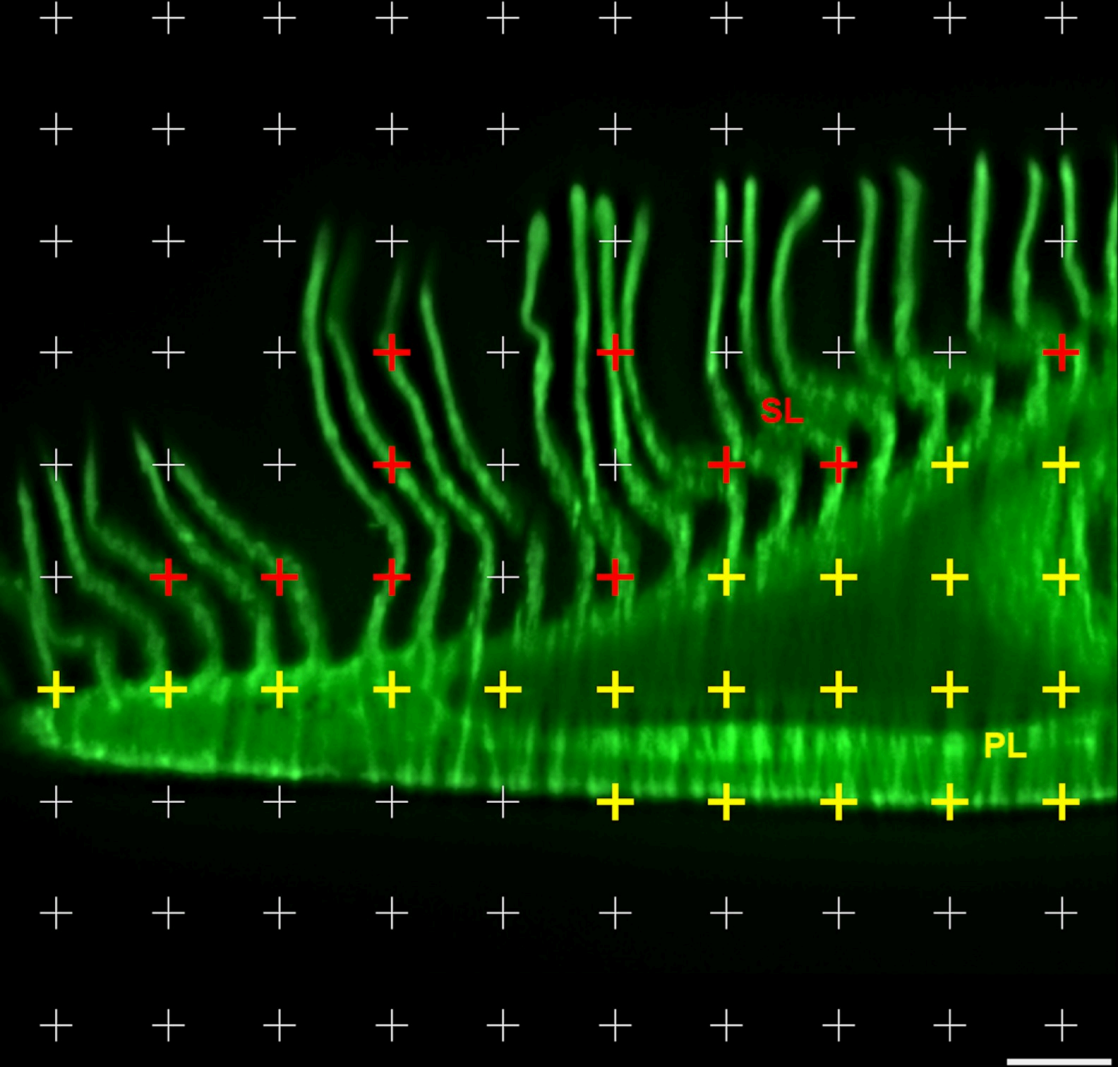
Estimation of V_V(SL/GF)_ in VUR autofluorescence images acquired by LSFM of optically cleared GF samples. For practical reasons, V_V(SL/GF)_ and S_V(SL/GF)_ can be estimated in the same images, *i*.*e*., using SUR sampled fields of view from (virtual) optical VUR sections. In the presented example of a SUR sampled field of view of a (virtual) GF VUR section, the vertical axis is not indicated. A (virtual) grid of equally spaced test points (crosses) is superimposed on the SUR sampled test field. All points hitting GF section profiles (P_(GF)_) (including PL section profiles (P_(PL)_, yellow crosses) and SL section profiles (P_(SL)_, red crosses)) are counted [in the presented example: 21 P_(PL)_ and 10 P_(SL)_, *i*.*e*., 31 P_(GF)_]. V_V(SL/GF)_ is calculated from P_(SL)_ and P_(GF)_, using **Eq 4**. LSFM-autofluorescence image acquired at 520/40 nm (ex) and 585/40 nm (em). Bar = 100 μm.

**Fig 20:**
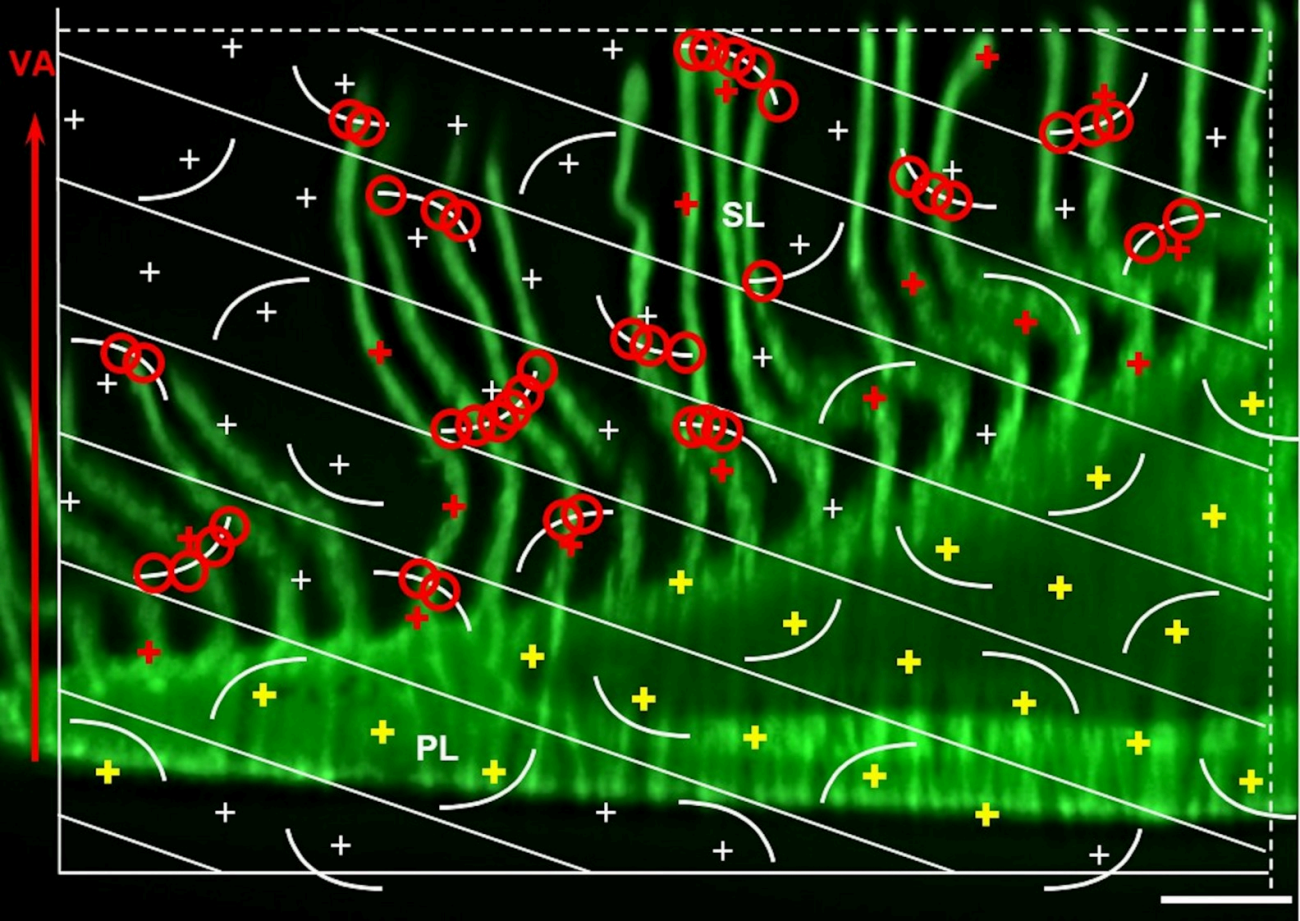
Estimation of S_V(SL/GF)_ in VUR autofluorescence images acquired by LSFM of optically cleared GF samples. Here, a SUR sampled field of view from a (virtual) optical VUR section is shown, generated as described in **Section 16.2** and **Fig 18**. The orientation of the vertical axis (**VA**, red arrow) and the size ruler are indicated. The VUR images are overlaid with a stereological test system combining cycloids and test points. The short side of the rectangular frame of the system is aligned parallel to the orientation of the VA. All points hitting GF section profiles (P_(GF)_, including PL section profiles (P_(PL)_, yellow crosses) and SL section profiles (P_(SL)_, red crosses)) are counted, as well as all intersections of cycloid arches with the epithelial surface of SL section profiles (I_(SL)_, encircled in red). [In the present example, a test system combining 35 cycloid arches and 70 points is used, refer to **Fig 13**. 21 P_(PL)_ and 16 P_(SL)_ (*i*.*e*., 37 P_(GF)_), and 41 I_(SL)_ are counted]. S_V(SL/GF)_ is calculated from the sum of intersections (∑I_(SL)_) and points (∑P_(GF)_), counted in all examined test fields of all sections of all samples per case, using **Eq 6**. LSFM-autofluorescence image acquired at 520/40 nm (ex) and 585/40 nm (em). Bar = 100 μm.

V_V(SL/GF)_ and S_V(SL/GF)_ are determined (**Figs 19** and **20**), using the same point grids and point/cycloid probes, respectively, as illustrated for the analysis of histological (VUR) sections (**Sections 12** and **13**). The total volume (V_(SL,GF)_) and (shrinkage corrected) surface area (S_(SL,GF)_) of the secondary lamellae (SL) in the gill filaments (GF) is calculated from V_V(SL/GF)_, respectively from S_V(SL/GF)_ and the total gill filament volume (V_(GF)_) (**Eqs 4–7**). The extent of 3DISCO-related gill filament tissue shrinkage is calculated from the measured V_(GF)_ prior to and after clearing, as described in **Section 16.1**.

Using the LSFM- and quantitative analysis methods described in **Section 16**, we determined a V_V(SL/GF)_ of 0.288, a (shrinkage-corrected) S_V(SL/GF)_ of 346.24 cm^2^/cm^3^, and a corresponding V_(SL,GF)_ of 0.818 cm^3^ and S_(SL,GF)_ of 983.32 cm^2^ in a healthy rainbow trout of ~1300 g body weight with a total gill filament volume of V_(GF)_ = 2.84 cm^3^ (**S1K Experimental data**).

The unbiasedness of the described approach for LSFM-based quantitative morphological analyses of optically cleared GF samples was confirmed by comparison of the estimates of V_V(SL/GF)_ acquired by LSFM analysis with the respective estimates determined by “classical” quantitative stereological analysis methods in SUR sampled gill samples of four fish, using the methods described above. Here, the estimates of V_V(SL/GF)_ obtained with both approaches were virtually equal, varying by only 2.23 ± 1.25% on the average (*p* = 0.806, paired t-test).

## Discussion

In ecotoxicology studies it is still common practice to limit histopathological and quantitative morphological gill analyses to samples taken from arbitrarily chosen locations and histological (paraffin-) sections with determined orientations, using semiquantitative scoring systems or simple 2-D morphometric analysis techniques [22,126–128]. However, such analysis approaches are inherently not capable to provide unbiased quantitative morphological data [14,25,70,129]. The large discrepancies of no observed effect concentrations (NOEC) partially based on the assessment of rainbow trout gill lesions in different studies examining the same compounds [7] substantiate the necessity for the establishment of standardized, unbiased, representative, reproducible and thus comparable analysis techniques for examination of quantitative morphological gill parameters in ecotoxicological studies. Assessment of quantitative morphological data by unbiased stereological analysis allows for an objective presentation of distinct tissue properties and their statistical comparability [8,14,129]. Quantitative stereological analyses can also identify subtle, yet probably (patho-) biologically relevant alterations (*e*.*g*., increased diffusion barrier thickness), which might escape subjective visual perception in standard histopathologic- or electron microscopic examination. The present work therefore presents the principles of quantitative stereological analyses and provides detailed descriptions of the determination of relevant histomorphological gill parameters in rainbow trout as a widely used fish species in ecotoxicological studies. The featured methods are based on established, state-of-the-art, unbiased (*i*.*e*., model-free) analysis and sampling methods [14] that have previously proven their general suitability for quantitative morphological analyses of gills of fish of various species and sizes [21,36–41]. The protocols presented here are adapted to rainbow trout of 300–2000 g body weight, which are frequently used in ecotoxicology studies. The sampling designs also ensure that enough gill filament material remains for additional analyses. In contrast, several previously published quantitative stereological analysis approaches scheduled a complete embedding of the gills, so that the gills under examination are not available for other analytical methods [36–40]. The methods described in the present work are of course also applicable to other fish species of comparable size and gill structure. The set of the featured quantitative morphological gill parameters (V_(GF)_, V_(SL,GF)_, S_(SL,GF)_, V_(EC,SL)_, N_(EC,SL)_, ${\bar{v}}_{\left(\mathrm{EC},\mathrm{SL}\right)}$, T_h(DB)_) covers the most relevant descriptors, effectively characterizing gill morphology. The described methods and protocols can easily be adapted to any other quantitative morphological parameter of interest in the context of a given study (*e*.*g*., numbers and cell volumes of additional distinct cell types or tissue structures in the gills). The analysis protocols were designed to allow for a feasible and expeditious analysis without restriction of the necessary precision. The recommended numbers of gill filament samples and sections to be generated, the microscopic magnification factors, the dimensions of the applicable stereological probes (*i*.*e*., sizes of cross grids and cycloid probes, areas of counting frames, disector heights) and the indicated numbers of points, intersections, and particles (Q^-^) to be counted for sufficiently precise quantitative stereological estimates were confirmed to warrant reliable analysis results without unnecessary sampling and analysis efforts.

Given the relevance of the precise volume measurement of samples (*i*.*e*., reference compartments) in quantitative stereological studies, we performed detailed analyses to ascertain practicable and accurate methods for volume determination of gill filament samples. Accordingly, we could demonstrate that briefly dabbing of gills on a lab-paper towel is sufficient to remove enough of the fluid attached to the (moist) gill to determine the weight of the gill sample with adequate precision for subsequent unbiased determination of the sample volume from its weight and density. The thus determined average density of formalin-fixed gill filaments of adult rainbow trout of 1.07 ± 0.02 g/cm^3^ may serve as an orientation value in subsequent studies, as data on the gill density have not yet been published.

The shrinkage of samples related to the embedding in histological plastic embedding media, such as GMA/MMA, must also be considered in analyses of any shrinkage-sensitive quantitative morphological parameter. In the present study, we applied unbiased analysis approaches to accurately determine the factual volumes of gill filament samples before (submersion volumetry) and after embedding in GMA/MMA (Cavalieri volumetry with histological sections of precisely measured thicknesses) to calculate the extent of trout gill filament tissue shrinkage related to GMA/MMA-embedding. The obtained result of f_s_ = 0.869 (corresponding to a 3-D volume shrinkage of ~34.31%) conforms to the linear tissue shrinkage factors determined for a variety of other biological tissues embedded in GMA/MMA [111,114]. However, the extent of tissue shrinkage related to histological plastic-embedding may vary, depending on the kind, size and processing of the samples, and the used embedding medium. For example, embedding of glutaraldehyde-fixed gill samples of the armored catfish (*Pterygoplichthys anisitsi*) in methacrylate (Historesin^®^) has previously been reported not to be associated with notable shrinkage [130]. In any quantitative stereological study, the embedding-related tissue shrinkage should therefore be regularly controlled in a sufficient number of representative samples, using appropriate analysis approaches.

The practical feasibility of the described quantitative stereological analysis methods was confirmed by their application on representative trout gill samples in the present work and in previous studies [99]. The obtained relative and absolute values of the examined quantitative stereological gill parameters provided in the present work are, however, not intended to serve as reference values for the rainbow trout in general, since these data may vary substantially with regard to the age, size, and body weight of the examined fish.

The classic unbiased quantitative stereological methods featured in the present guidelines have been described in the 1990`s and earlier. However, these methods still present the gold standard for quantitative morphological analyses in several live science disciplines [25,27]. Nevertheless, the practical implementation of these methods is often cumbersome, work-intensive and time consuming, particularly due to the specific sampling procedures and tissue processing steps associated with quantitative stereological analyses. In recent years, gross and histopathological evaluation has moved fast forward driven by the developments in virtual microscopy, digital image analysis and modern communication technologies [131–133]. Here, we present the application of LSFM imaging of optically cleared tissue samples as a modern, innovative, fast and efficient approach for quantitative morphological analyses of the gills.

For the first time the application of LSFM of optically 3DISCO-cleared trout gill samples is presented, for a straightforward determination of relevant quantitative morphological gill parameters without the need for generation of physical sections. The applied 3DISCO clearing protocol [51] and the LSFM imaging procedure are fast and easily to perform and allow for an excellent 3-D visualization of the complex microscopic gill architecture without additional (*in-* or *ex-vivo*) fluorescent labeling. For the analysis of quantitative morphological gill parameters by LSFM, digital images of virtual optical (autofluorescence) sections of the cleared gill filament samples are analyzed, whereas the random sampling designs and stereological probes are the same as used in the “classical” quantitative stereological analysis approaches. Considering the extent of 3-D volume shrinkage of gill samples associated with the applied 3DISCO clearing protocol (50.72 ± 2.88%), the quantitative morphological analysis results obtained by LSFM analysis are virtually identical to the estimates obtained by “classical” unbiased quantitative stereological analyses of the identical gills, thus proving the applicability of LSFM for quantitative morphological gill analyses. With the significant reduction of the required sample processing steps and the associated expenditure of time, the benefit of the LSFM-based approach for quantitative morphological gill analyses is clearly confirmed. Whereas *e*.*g*., GMA/MMA-embedding of gill samples and subsequent preparation of histological sections usually takes more than 3 days, 3DISCO clearing and LSFM imaging of gill samples is completed within 1–2 days, while the costs of the necessary laboratory consumables and chemicals are comparable. In combination with standard histological analyses of (SUR sampled) FFPE gill filament samples for qualitative histopathological evaluation, LSFM-based analyses can thus significantly contribute to a fast, reliable and unbiased analysis of quantitative morphological gill parameters, which is particularly important for ecotoxicological studies examining high numbers of samples. Remarkably, LSFM analyses of 3DISCO-cleared specimens can be combined with additional (multimodal) histo-technical analyses. Subsequent to LSFM analysis, optically cleared samples can *e*.*g*., be re-embedded in paraffin (or other embedding media) and sectioned for subsequent standard histological as well as immunohistological analyses [134]. Conversely, it is also possible to clear and image (gill) samples that had previously been embedded in paraffin (following deparaffinization of the samples) [135]. For LSFM analysis of optically cleared samples, diverse commercial software tools for 3-D image reconstruction and image analysis are available. These programs also include functions for (semi-) automatic analysis of *e*.*g*., volumes, surfaces and numbers of delimitably identifiable tissue structures, such as specifically fluorescently labeled cells or tissue compartments. Combined with appropriate sampling designs, these software tools can be applied for rapid digital analyses of quantitative morphological parameters, *e*.*g*., for counting and sizing of kidney glomeruli after *in-vivo* labeling with fluorescence-labeled antibodies [57]. For LSFM-based analyses of the interested quantitative morphological parameters in (unlabeled) cleared gill samples, automatic digital image analysis is, however, not applicable, as long as no specific fluorescent labeling of distinct gill structures of interest, such as distinct cell types, is available.

## Conclusion

In summary, the present guidelines thus represent a solid base for standardized, objective quantitative morphological analyses of rainbow trout gills. The broad implementation of the featured methods will significantly contribute to the representativity, unbiasedness, reliability and comparability of analyses results in ecotoxicology studies reporting quantitative morphological gill parameters and therefore add urgently required certainty to the detection of NOEC values as a base for the specification of legal concentration limits of aquatic pollutants. Additionally, the application of the described protocols can help to lower the required number of experimental fishes by avoiding unnecessary repetitions of experiments or studies.

## Supporting information

S1 Fig 3-D architecture of gills demonstrated by laser light sheet microscopy (LSFM) of a solvent-cleared gill. (DOCX)

S2 Fig Cardiac vascular perfusion fixation of rainbow trout gills. (DOCX)

S3 Fig Histological perfusion fixation artifacts in rainbow trout gills. (DOCX)

S4 Fig Ultrastructural perfusion fixation artifacts in rainbow trout gills. (DOCX)

S5 Fig Determination of the gill volume/density in consideration of the attached liquid volume. (DOCX)

S6 Fig Photographic illustration of the determination of the gill volume/density in consideration of attached liquid volume. (DOCX)

S7 Fig Processing of a SUR sampled gill filament sample for generation of IUR sections (*Isector* method). (DOCX)

S8 Fig Processing of a SUR sampled gill filament sample for generation of GMA/MMA-embedded VUR sections. (DOCX)

S9 Fig Ruler copy templates suitable for analysis of apparent diffusion barrier section profile distances. (DOCX)

S10 Fig Determination of T_h(DB)_ in secondary gill lamellae in semithin IUR sections (light microscopic analysis). (DOCX)

S11 Fig Appearance of GF profiles in different vertical section planes (VSP) of virtual 3-D GF-reconstructions. (DOCX)

S1 Table Dimensions of ruler (divisions equidistant on log reciprocal scale). (DOCX)

S2 Table 3DISCO clearing of rainbow trout gills. (DOCX)

S1 Video 3-D reconstruction of rainbow trout gills. (MP4)

S1 Experimental data (DOCX)

S1 Eq Concentration determination by Lambert-Beer Law. (DOCX)

S2 Eq Determination of dyed liquid volume after dilution. (DOCX)
