# Supplementary material for: A practical guide to unbiased quantitative morphological analyses of the gills of rainbow trout (Oncorhynchus mykiss) in ecotoxicological studies
Source: PLoS One. 2020 Dec 9;15(12):e0243462. doi: 10.1371/journal.pone.0243462 (PMC7725368; doi:10.1371/journal.pone.0243462)
Supplement: S1 Fig — (DOCX) [file pone.0243462.s001.docx]

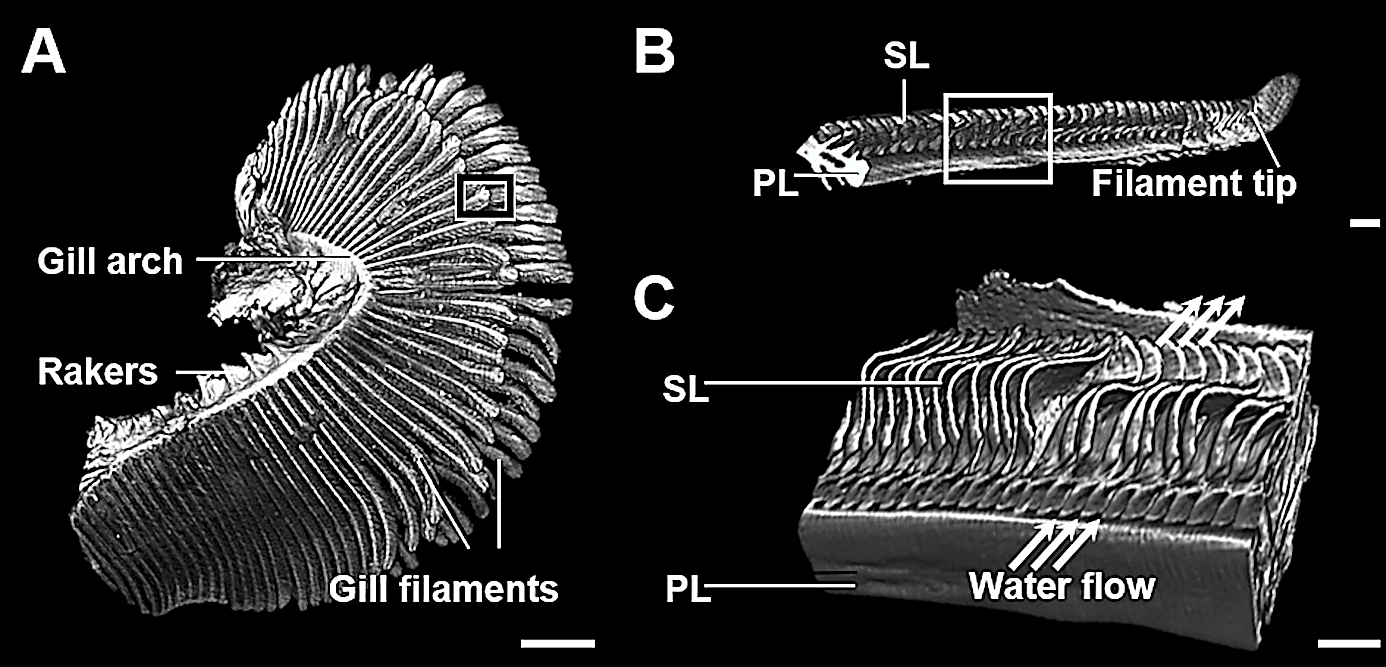


**S1 Fig. 3-D architecture of gills demonstrated by laser light sheet microscopy (LSFM) of a solvent-cleared gill.**

Autofluorescence images of an optically cleared (3DISCO) gill sample are acquired at 520/40 nm excitation range (ex) and 585/40 nm emission range (em). Important morphological structures and the direction of water flow are indicated: **SL**: Secondary lamellae, **PL**: Primary lamellae. **A.** Isolated gill. Note the arrangement of gill filaments in two vertical rows *(i.e.,* hemibranchs). **B.** Detail enlargement of the tip of a gill filament (as indicated in A). **C.** Detail enlargement of a gill filament with secondary lamellae (as indicated in B). Bars = 1 cm in A and = 100 µm in B and C.
