# Supplementary material for: A practical guide to unbiased quantitative morphological analyses of the gills of rainbow trout (Oncorhynchus mykiss) in ecotoxicological studies
Source: PLoS One. 2020 Dec 9;15(12):e0243462. doi: 10.1371/journal.pone.0243462 (PMC7725368; doi:10.1371/journal.pone.0243462)
Supplement: S2 Fig — (DOCX) [file pone.0243462.s002.docx]

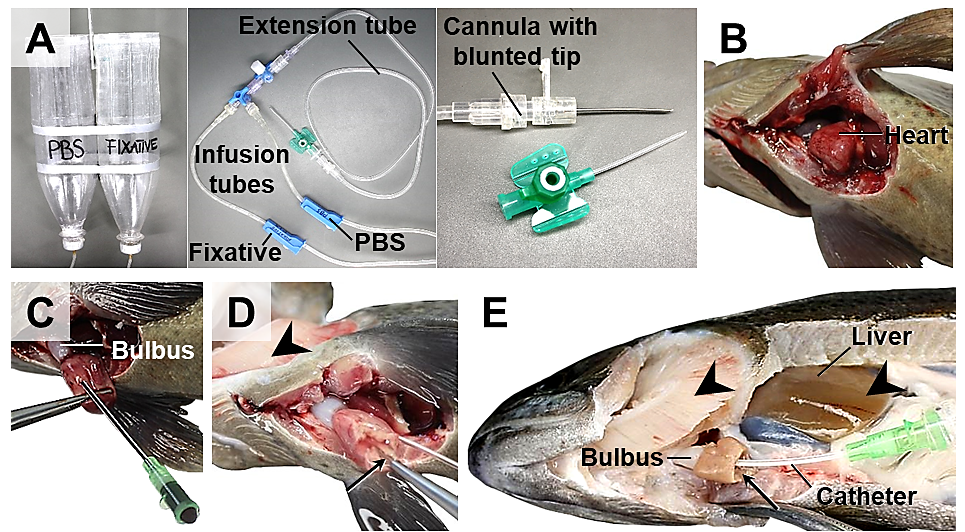


**S2 Fig. Cardiac vascular perfusion fixation of rainbow trout gills.**

Perfusion fixation with formaldehyde-containing fixation solutions should be performed under a laboratory hood or on a well-ventilated necropsy desk, wearing personal protective gear (lab coat, gloves, goggles, respiratory protective mask). **A.** Equipment used for vascular perfusion fixation [1]. Two transparent plastic containers (soda-pop bottles) with cut-off bottoms are connected with infusion tubes and 3-way valves. The bottles are labeled appropriately (buffer and fixation solution) and installed approximately 70 cm above the level of the workbench. The buffer container is filled with chilled (~10°C) phosphate buffered saline (310 mosmol PBS, pH 7.4) [2-4], the fixation solution container is filled with neutrally buffered formaldehyde solution and the tubes are vented. A large diameter (17-20 Gauge) intravenous catheter (Braun^®^Vasofix^®^ safety, B.Braun Melsungen AG, Germany) with a slightly grinded tip (to prevent an unintended penetration of the heart ventricle wall during perfusion) is used as perfusion cannula. To generate a perfusion pressure of approximately 40 mmHg (corresponding to the blood pressure of the ventral aorta [5, 6], the trout is placed approximately 54 cm below the perfusion containers, immediately after euthanasia. **B.** Ventro-lateral dissection of the heart and the bulbus arteriosus. **C.** After incision of the transversal septum and the pericard, the heart ventricle is punctured with the perfusion catheter cannula. Care must be taken not to pierce the bulbus or the ventral aorta. **D.** The cannula is removed and the flexible catheter is carefully pushed forward into the bulbus arteriosus to prevent backflow of buffer and fixative solution. The catheter is then connected to the (buffer-filled) perfusion tube. When perfusion with PBS is started, the heart ventricle is pierced below the entry site of the catheter (arrow in D, E) to create an outflow for the perfusate. PBS-perfusion is continued until the color of gills and liver turns into pale (arrowheads in D, E) and the outflowing perfusate is transparent. Subsequently, the valve of the fixative-solution bearing tube is opened before the valve of the PBS-tube is closed. **E.** Perfusion with fixation solution is continued for approximately 3 minutes. Gills are then dissected and further fixed by immersion as described in **Section 7**.

1. Albl B, Haesner S, Braun-Reichhart C, Streckel E, Renner S, Seeliger F, et al. Tissue Sampling Guides for Porcine Biomedical Models. Toxicol Pathol. 2016;44(3): 414-420.

2. Byrne P, Speare D, Ferguson HW. Effects of a cationic detergent on the gills and blood chemistry of rainbow trout Salmo gairdneri. Dis Aquat Org. 1989;6: 185-196.

3. Hille S. A literature review of the blood chemistry of rainbow trout, Salmo gairdneri Rich. J Fish Biol. 1982;20(5): 535-569.

4. Olson KR, Fromm PO. Preparation of fish tissues for electron microscopy. J Electron Microsc Tech. 1985;2(3): 217-228.

5. Kiceniuk JW, Jones DR. The Oxygen Transport System in Trout (Salmo Gairdneri) During Sustained Exercise. J Exp Biol. 1977;69(1): 247-260.

6. Wood CM, Shelton G. Cardiovascular dynamics and adrenergic responses of the rainbow trout in vivo. J Exp Biol. 1980;87: 247-270.
