# Supplementary material for: A practical guide to unbiased quantitative morphological analyses of the gills of rainbow trout (Oncorhynchus mykiss) in ecotoxicological studies
Source: PLoS One. 2020 Dec 9;15(12):e0243462. doi: 10.1371/journal.pone.0243462 (PMC7725368; doi:10.1371/journal.pone.0243462)
Supplement: S3 Fig — (DOCX) [file pone.0243462.s003.docx]

**
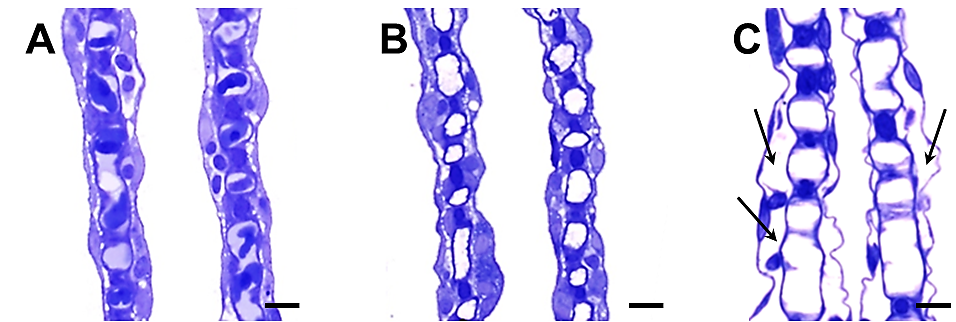
**

**S3 Fig. Histological perfusion fixation artifacts in rainbow trout gills.**

**A. Histology of secondary gill lamellae of a control trout (non-perfused).** Note the presence of nucleated erythrocytes inside the capillaries. **B, C. Histology of perfusion-fixed secondary gill lamellae.** **B.** Adequate preservation of the morphology of gills perfused with ~40 mmHg perfusion pressure. **C.** Severe detachment of the gill epithelium in gills perfused with ~100 mmHg perfusion pressure (arrows). Semithin sections (0.5 µm thickness). Epon. TB. Bars = 10 µm.
