# Supplementary material for: A practical guide to unbiased quantitative morphological analyses of the gills of rainbow trout (Oncorhynchus mykiss) in ecotoxicological studies
Source: PLoS One. 2020 Dec 9;15(12):e0243462. doi: 10.1371/journal.pone.0243462 (PMC7725368; doi:10.1371/journal.pone.0243462)
Supplement: S4 Fig — (DOCX) [file pone.0243462.s004.docx]

**
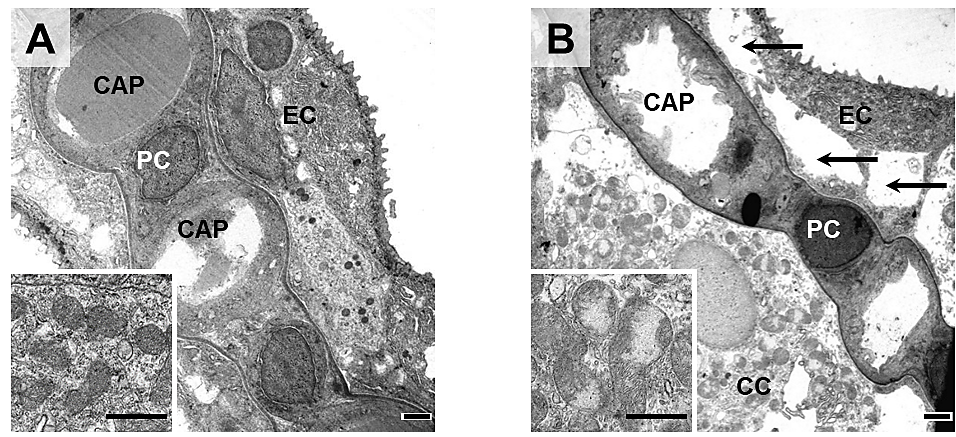
**

**S4 Fig. Ultrastructural perfusion fixation artifacts in rainbow trout gills.**

Representative transmission electron microscopic images of secondary lamellae of a control fish
(**A**, non-perfused), and of a gill perfused with ~100 mmHg (**B**). Capillary (CAP), pillar cell (PC), epithelial cell (EC), chloride cell (CC). Note the detachment of the gill epithelium in **B** (arrows). Inset in B shows severely distended and swollen mitochondria of a chloride cell. TEM. Bars = 1 µm.
