# Supplementary material for: A practical guide to unbiased quantitative morphological analyses of the gills of rainbow trout (Oncorhynchus mykiss) in ecotoxicological studies
Source: PLoS One. 2020 Dec 9;15(12):e0243462. doi: 10.1371/journal.pone.0243462 (PMC7725368; doi:10.1371/journal.pone.0243462)
Supplement: S5 Fig — (DOCX) [file pone.0243462.s005.docx]

### **
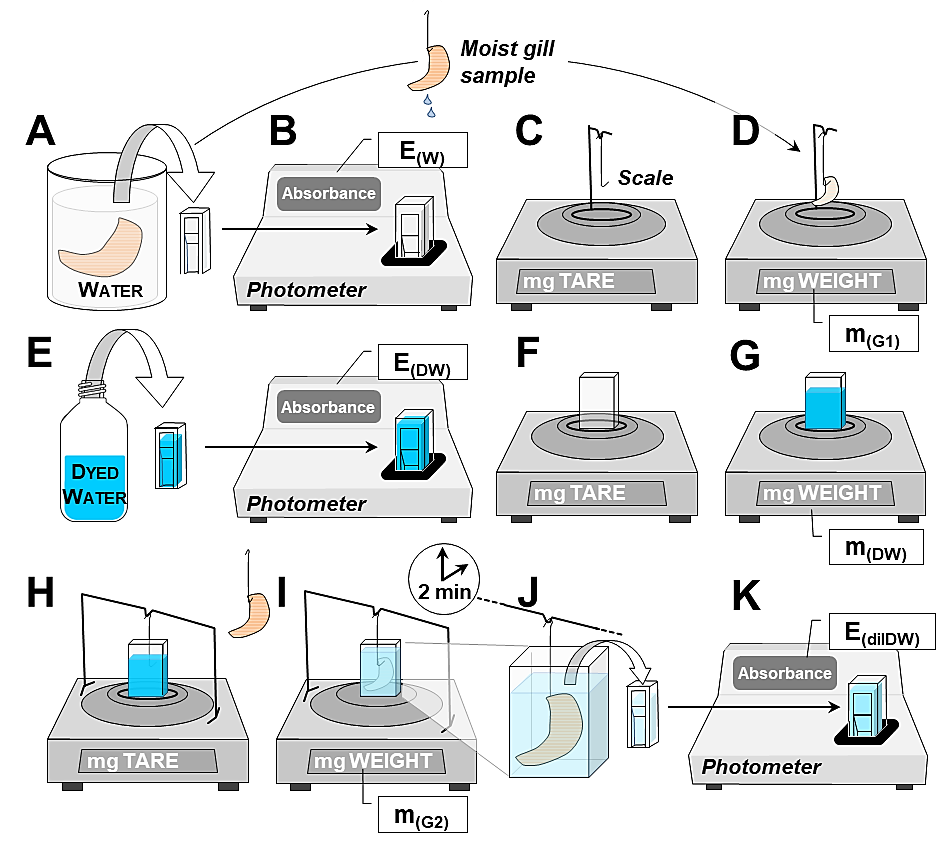
**

### **S5 Fig. Determination of the gill volume/density in consideration of the attached liquid volume.**

### The filamentous-lamellar structure of gills favors binding of large amounts of fluid. When determining the weight or the volume of a tissue sample, the volume of liquid attached to the sample will thus bias the measurement results. The liquid volume attached to a sample of gill tissue can be determined by photometric measurement of the concentration of a dyed fluid before and after submersion of the tissue sample. **A.** The gill tissue sample is transferred to a container with (tab) water. **B.** The absorbance of the water (**E_(W)_**) is determined photometrically. **C, D.** The drained sample weight (including attached water) is measured and recorded (**m_(G1)_**) after taring the scale to the weight of the sample holder. **E.** The absorbance (**E_(DW)_**) of the submersion liquid, a solution of water dyed with 7.5 mg/l Acid Blue 9 (Alfa Aesar by Thermo Fisher GmbH, Germany) is determined (here: **E_(DW_**_)_= 0.666 at 630 nm wavelength). **F, G.** After taring the scale to the weight of the submersion container, the weight of the volume of dyed submersion liquid in the submersion-container is measured (**m_(DW)_**). **H.** A sample holder is positioned on the scale as indicated, so that the wire used to hold the gill sample is sufficiently submerged in the submersion fluid without touching the sides or the bottom of the container. The scale is then zeroed. Note that the sample holder is not placed on the sensor of the scale. **I.** The gill tissue sample is attached to the sample holder, so that the tissue is completely submerged in the submersion fluid without touching the sides or the bottom of the container. After equilibration for approximately two minutes, the weight (representing the cumulative weight of the fluid displaced by the submerged tissue and of the liquid that was attached to the gill tissue) is recorded (**m_(G2)_**). **J.** The absorbance (**E_(dilDW)_**) of the submersion fluid (diluted by the fluid volume that was attached to the gill tissue sample) is determined by photometric measurement and used to calculate the dye concentration in the fluid according to Lambert-Beer law. The quantity of water attached to the gills is determined from the decrease of the dye concentration in the submersion fluid (**S2 Eq**).
