# Supplementary material for: A practical guide to unbiased quantitative morphological analyses of the gills of rainbow trout (Oncorhynchus mykiss) in ecotoxicological studies
Source: PLoS One. 2020 Dec 9;15(12):e0243462. doi: 10.1371/journal.pone.0243462 (PMC7725368; doi:10.1371/journal.pone.0243462)
Supplement: S6 Fig — (DOCX) [file pone.0243462.s006.docx]

**
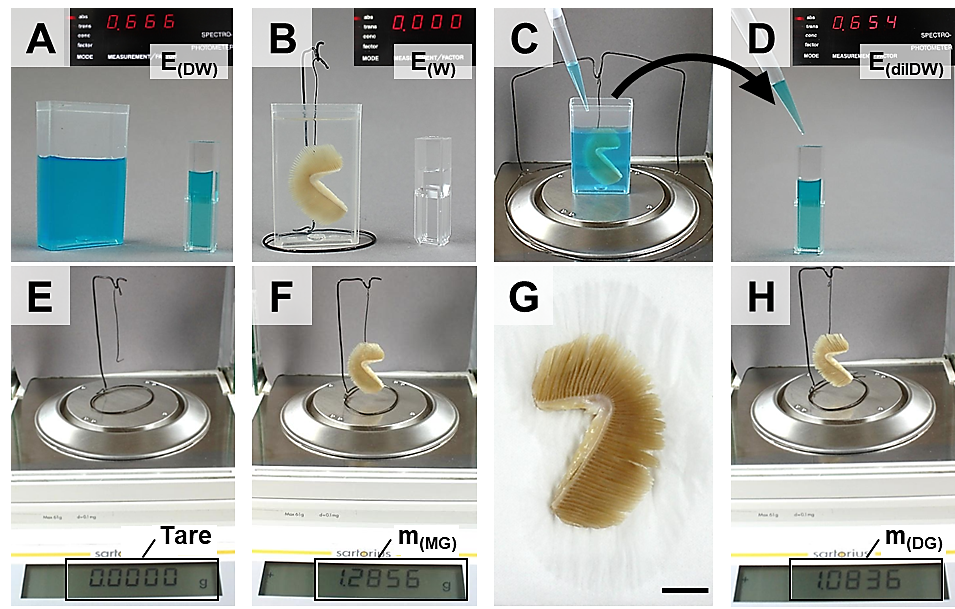
**

**S6 Fig.** **Photographic illustration of the determination of the gill volume/density in consideration of attached liquid volume.**

Container with Acid Blue-9 dyed submersion liquid of known concentration (here: 7.5 mg/l). The dyed submersion liquid has an absorbance of 0.666 (**E_(DW)_**) at 630 nm wavelength. **B.** Gill submerged in water (absorbance of water **E_(W)_** = 0.000). **C.** The gill tissue sample is submerged in dyed submersion liquid, which is diluted by the (undyed) water attached to the gill tissue sample. Previously, the weight of the moist gill tissue sample and the weight of the dyed submersion liquid were measured (not shown here, compare to **S5 Fig**). **D.** Measurement of the absorbance of the diluted submersion liquid (here: **E_(dilDW)_** = 0.654). The dye concentration of the diluted submersion liquid is calculated according to Lambert-Beer law (**S1 Eq**). The volume of (undyed) water that is necessary to cause this concentration decrease (*i.e.,* the volume of the water attached to the gill sample prior to submersion in the submersion liquid) is calculated by subtraction of the dyed liquid volume prior to dilution (**V_P_**) from the diluted dyed liquid volume (*i.e.,* after submersion) (**V_A_**), **V_A_** is calculated from the decrease in concentration previously determined by photometric measurement (**S2 Eq**). **E-F.** The weight of a moist gill tissue sample (including the liquid adhering to the sample is determined (**m_(MG)_**). **G-H.** The moist gill tissue sample is gently blotted dry on a lab-paper towel for approximately 10 seconds and subsequently weighed (**m_(DG)_**). To test the efficacy of the dry-dabbing to remove the water adhering to the gill tissue sample, the volume of the dry-dabbed sample is determined by the submersion method as shown in **Fig 7** and compared to the volume of the identical gill tissue sample corrected for the volume of adhering liquid. Note that for demonstration purposes, the images show gills with incompletely removed gill arches. For analyses of quantitative morphological gill parameters, however, it is recommended to determine the weight, density, and volume of gill filament tissue as the appropriate reference compartment (after complete removal of the gill arches from the gills). Bar = 1 cm.
