# Supplementary material for: A practical guide to unbiased quantitative morphological analyses of the gills of rainbow trout (Oncorhynchus mykiss) in ecotoxicological studies
Source: PLoS One. 2020 Dec 9;15(12):e0243462. doi: 10.1371/journal.pone.0243462 (PMC7725368; doi:10.1371/journal.pone.0243462)
Supplement: S7 Fig — (DOCX) [file pone.0243462.s007.docx]

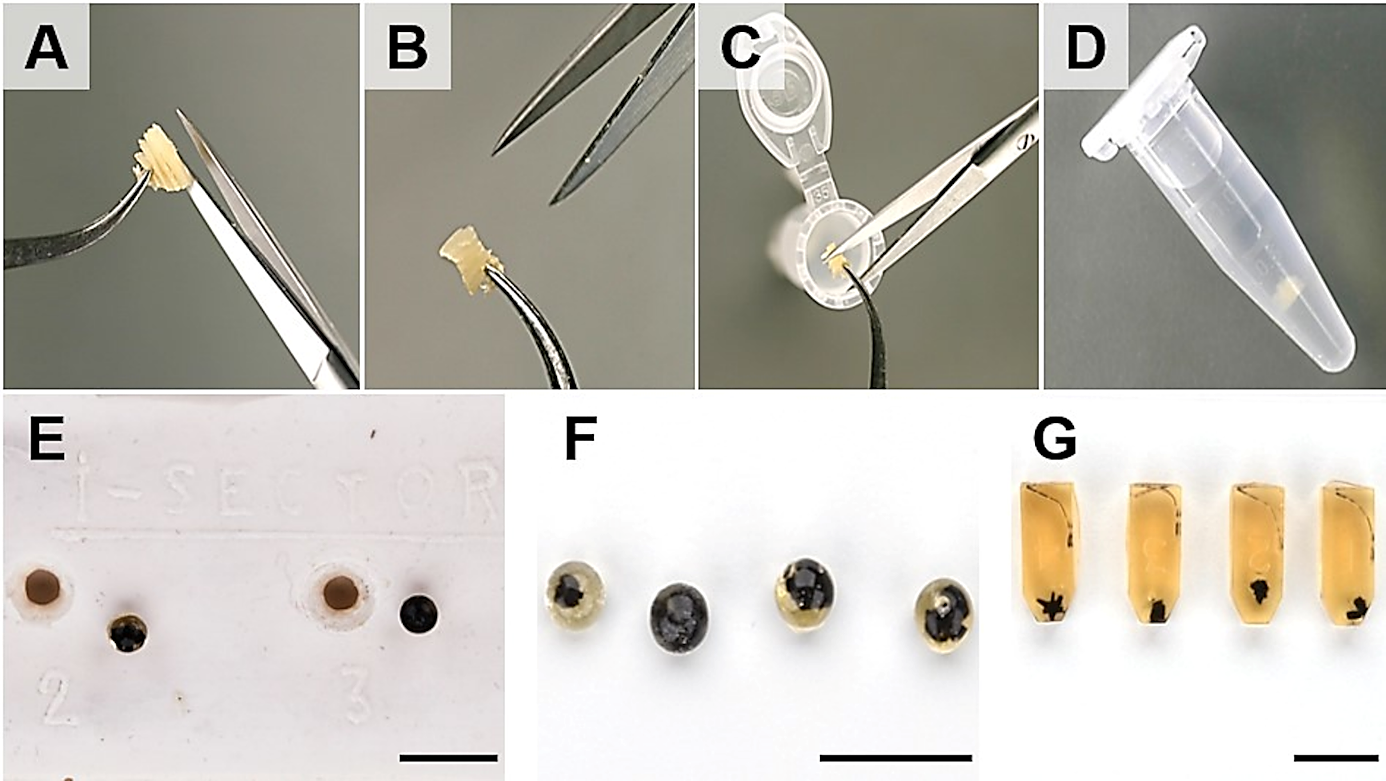


**S7 Fig. Processing of a SUR sampled gill filament sample for generation of IUR sections (*Isector* method).**

**A-D.** GF samples are carefully cut to a size of approximately 1 mm^3^ for subsequent electron microscopic studies. Sharp scissors are used to prevent artificial damage of the fragile secondary gill lamellae. If electron microscopic analyses are scheduled, the samples are postfixed in 2.5% glutaraldehyde dissolved in 0.1 M cacodylate buffer (**D**). **E.** The samples are embedded in Epon, using spherical casting molds. **F.** Polymerized Epon spheres with embedded gill filament tissue samples (compare to **Fig 9**). **G.** After randomization of their orientation, the Epon spheres are embedded in larger Epon blocks for further sectioning of semi- or ultrathin sections. Bars = 1 cm.
