# Supplementary material for: A practical guide to unbiased quantitative morphological analyses of the gills of rainbow trout (Oncorhynchus mykiss) in ecotoxicological studies
Source: PLoS One. 2020 Dec 9;15(12):e0243462. doi: 10.1371/journal.pone.0243462 (PMC7725368; doi:10.1371/journal.pone.0243462)
Supplement: S8 Fig — (DOCX) [file pone.0243462.s008.docx]

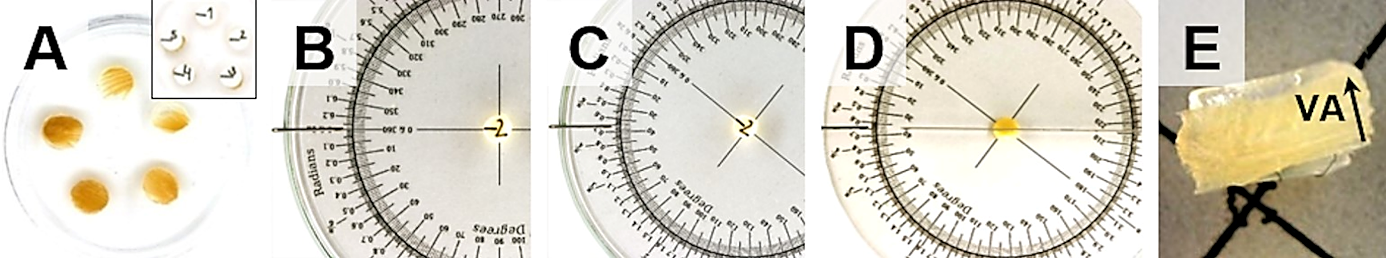


**S8 Fig. Processing of a SUR sampled gill filament sample for generation of GMA/MMA-embedded VUR sections.**

**A.** SUR sampled specimens of agar-embedded gill filament tissue, excised with a biopsy punch. The numbers of the samples and the 0°-180°-lines are marked on the confetti paper placed on the samples (inset). **B-D.** Systematic uniform random rotation and VUR sectioning of samples. The samples are placed on an equiangular circle, corresponding to the 0°-180°-mark on the confetti paper (here: sample N°2, compare to **Fig 10**) (**B**). A vertical section (parallel to the VA) is cut through the sample at the predefined rotation angle (here: 38°). **E.** Section surface of the gill filament tissue sample (embedded in agar for stabilization). VA is still identifiable.
