# Supplementary material for: A practical guide to unbiased quantitative morphological analyses of the gills of rainbow trout (Oncorhynchus mykiss) in ecotoxicological studies
Source: PLoS One. 2020 Dec 9;15(12):e0243462. doi: 10.1371/journal.pone.0243462 (PMC7725368; doi:10.1371/journal.pone.0243462)
Supplement: S9 Fig — (DOCX) [file pone.0243462.s009.docx]

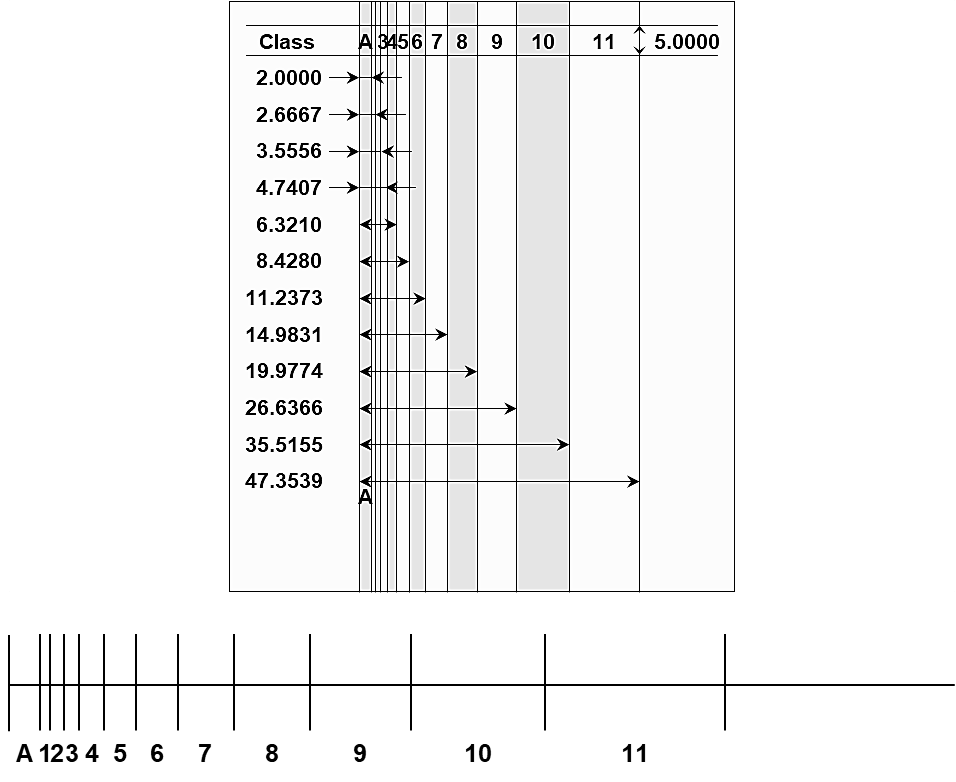


**S9 Fig. Ruler copy templates suitable for analysis of apparent diffusion barrier section profile distances.**

Note that the copy template is adjusted to a foil size of 210 mm x 297 mm (DIN-A4). If transparencies of 8.5 x 14 inch (US-Legal) are used, the settings of the photocopier have to be adjusted accordingly (on the printed ruler, the classes must have the indicated dimensions).
