# Supplementary material for: A practical guide to unbiased quantitative morphological analyses of the gills of rainbow trout (Oncorhynchus mykiss) in ecotoxicological studies
Source: PLoS One. 2020 Dec 9;15(12):e0243462. doi: 10.1371/journal.pone.0243462 (PMC7725368; doi:10.1371/journal.pone.0243462)
Supplement: S10 Fig — (DOCX) [file pone.0243462.s010.docx]

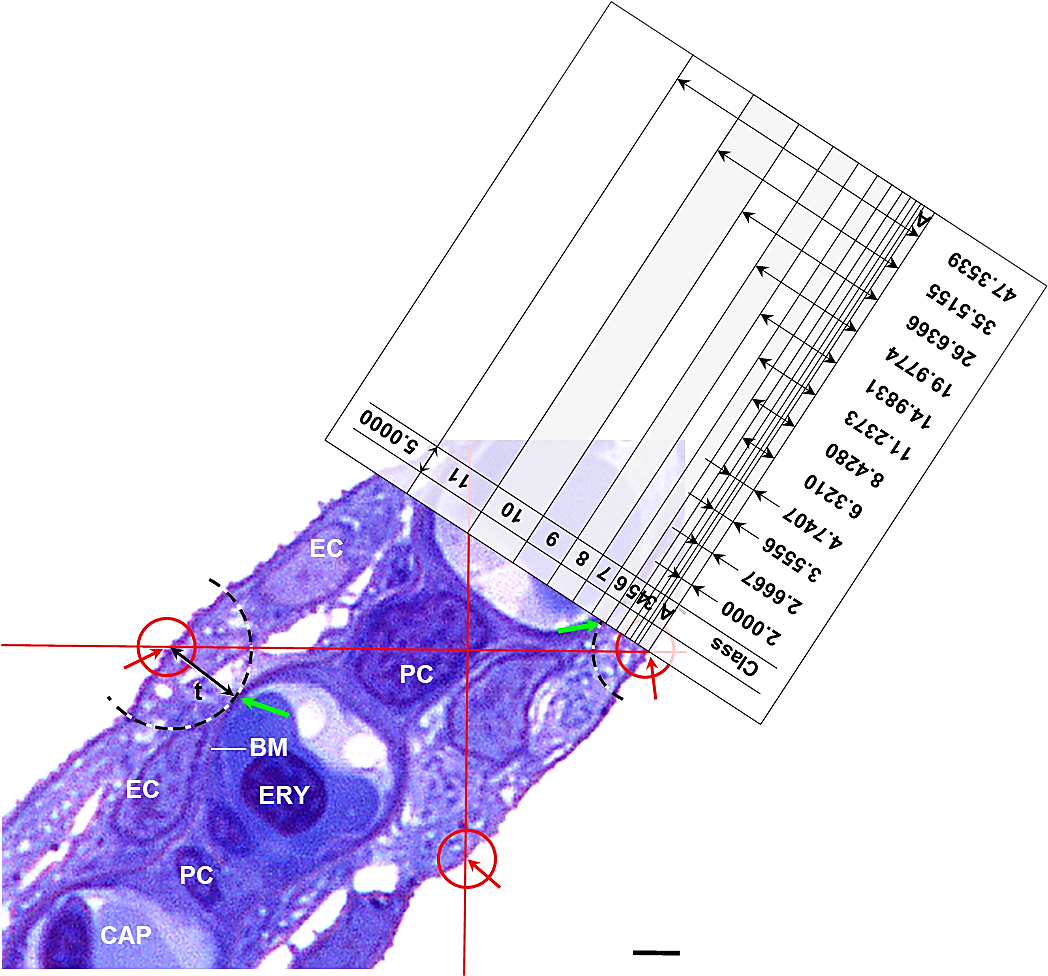


**S10 Fig. Determination of T_h(DB)_ in secondary gill lamellae in semithin IUR sections (light microscopic analysis).**

A printed image of a SUR sampled field of view of an IUR semithin section of a SL acquired at 1000x magnification (oil immersion) is superimposed with a test grid of lines (red). Note that the final print magnification of the analyzed images must be determined for calculation of the true harmonic mean of the diffusion barrier thickness. The transections of the gridlines with the epithelial surface of the SL are marked by red arrows. At these locations, the shortest distance (t, double arrow) between the epithelial surface of the SL and the inner surface of the vascular space of the gill capillary (green arrows) is determined (dashed circles). Along these lines, the diffusion barrier thickness is measured using a superimposed, transparent, logarithmic ruler subdivided into 12 classes. In the shown example, the measured distance falls in class 6. The true harmonic mean of the diffusion barrier thickness is calculated from the number of measurements and the corresponding classes (**Eq 13**).
Semithin section (0.5 µm thickness). Epon. TB. Bar = 2 µm.
