# Supplementary material for: A practical guide to unbiased quantitative morphological analyses of the gills of rainbow trout (Oncorhynchus mykiss) in ecotoxicological studies
Source: PLoS One. 2020 Dec 9;15(12):e0243462. doi: 10.1371/journal.pone.0243462 (PMC7725368; doi:10.1371/journal.pone.0243462)
Supplement: S11 Fig — (DOCX) [file pone.0243462.s011.docx]

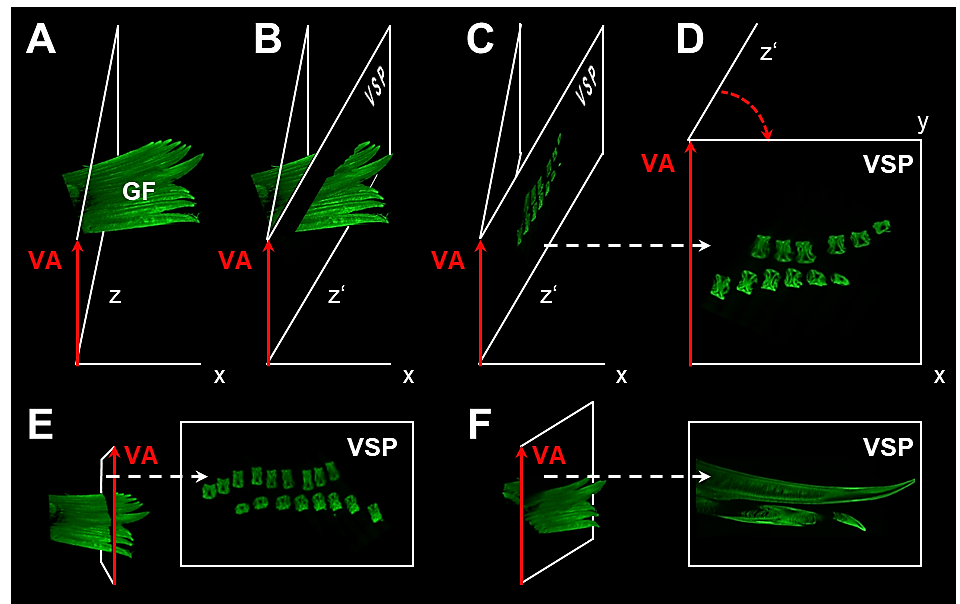


**S11 Fig. Appearance of GF profiles in different vertical section planes (VSP) of virtual 3-D GF-reconstructions.**

**A.** 3-D image of GF tissue, reconstructed from LSFM autofluorescence images of a 3DISCO-cleared gill filament sample. The orientation of a (user-defined) vertical axis (VA) relative to the sample is indicated by a red arrow. The directions of the corresponding x- and z-axes are indicated. **B.** The orientation of a random VSP (VA-z’ plane) parallel to the VA is indicated. **C.** Section profiles of gill filaments in the VSP. Note that the profile areas are distorted due to the projection to the viewing plane. **D.** Appearance of the perpendicular aspect of the GF-VSP profiles after (virtually) turning the VSP around the VA.
**E, F.** Appearance of GF profiles in different VSP orientations (orthogonal (**E**) and parallel (**F**) VSP orientation relative to the longitudinal axis of the gill filaments). Note that the purpose of this figure is only to demonstrate the different appearance of VSP section profiles of GF tissue samples. For quantitative stereological analyses of S_V(SL/GF)_, optical VUR sections must be generated as shown
in **Fig 18**.
