# Supplementary material for: A practical guide to unbiased quantitative morphological analyses of the gills of rainbow trout (Oncorhynchus mykiss) in ecotoxicological studies
Source: PLoS One. 2020 Dec 9;15(12):e0243462. doi: 10.1371/journal.pone.0243462 (PMC7725368; doi:10.1371/journal.pone.0243462)
Supplement: S1 Table — (DOCX) [file pone.0243462.s012.docx]

**S1 Table. Dimensions of ruler (divisions equidistant on log reciprocal scale).**

| **Class** | **Lower Limit, mm** | **1/Lower Limit, mm** | **Midpoint, mm** |
| --- | --- | --- | --- |
| **Class A** | 2.000 | 0.5000 | 0.4375 |
| **Class 1** | 2.6667 | 0.3750 | 0.3281 |
| **Class 2** | 3.5556 | 0.2813 | 0.2461 |
| **Class 3** | 4.7407 | 0.2109 | 0.1846 |
| **Class 4** | 6.3210 | 0.1582 | 0.1384 |
| **Class 5** | 8.4280 | 0.1187 | 0.1038 |
| **Class 6** | 11.2373 | 0.0890 | 0.0779 |
| **Class 7** | 14.9831 | 0.0667 | 0.0584 |
| **Class 8** | 19.9774 | 0.0501 | 0.0438 |
| **Class 9** | 26.6366 | 0.0375 | 0.0328 |
| **Class 10** | 35.5155 | 0.0282 | 0.0246 |
| **Class 11** | 47.3539 | 0.0211 | 0.0211 |

***Class A:*** *The initial division in which no measurement of the diffusion barrier (DB) can be obtained.* ***Class 1-11:*** *Ruler classes.* ***Lower Limit:*** *Begin of the next ruler class in size, measured from ruler origin.* ***Midpoint of classes n:*** *((1/Lower Limit of Class n) + (1/Lower Limit of Class n+1))/2.*
