# Supplementary material for: A practical guide to unbiased quantitative morphological analyses of the gills of rainbow trout (Oncorhynchus mykiss) in ecotoxicological studies
Source: PLoS One. 2020 Dec 9;15(12):e0243462. doi: 10.1371/journal.pone.0243462 (PMC7725368; doi:10.1371/journal.pone.0243462)
Supplement: S2 Table — (DOCX) [file pone.0243462.s013.docx]

**S2 Table. 3DISCO clearing of trout gill tissue (modified according Ertürk et al. [1]).**

| **Chemical** | **Time** | **Remarks** |
| --- | --- | --- |
| Starting material: Formalin-fixed gill filament tissue samples (clear tissue prior to SURS) | | |
| Tab water | 1h | Wash out of fixative |
| 50% THF | 1h |  |
| 70% THF | 1h |  |
| 80%THF | 1h |  |
| 100% THF | 1h |  |
| 100% THF | over night |  |
| 100% THF | 1h |  |
| 100% DCM | 30 min^a^ | ^a^until tissue sample sinks |
| BABB | 20 |  |
| BABB | ≥3h |  |
| *Store in BABB in the dark at 4-8°C. Use BABB as medium for LSFM analysis and allow for temperature equilibration of the sample before starting image acquisition.* | | |

**SURS**: Systematic uniform random sampling; **THF**: Tetrahydrofuran; **DCM**: Dichloromethane; **BABB**: Benzyl alcohol-benzyl benzoate.

1. Ertürk A, Becker K, Jährling N, Mauch CP, Hojer CD, Egen JG, et al. Three-dimensional imaging of solvent-cleared organs using 3DISCO. Nat Protoc. 2012;7(11): 1983-1995.
