# Supplementary material for: A practical guide to unbiased quantitative morphological analyses of the gills of rainbow trout (Oncorhynchus mykiss) in ecotoxicological studies
Source: PLoS One. 2020 Dec 9;15(12):e0243462. doi: 10.1371/journal.pone.0243462 (PMC7725368; doi:10.1371/journal.pone.0243462)
Supplement: S1 Experimental data — (DOCX) [file pone.0243462.s015.docx]

**S1 Experimental Data**

**A. Comparison of V_V(SL/GF)_ in left and right gills.**

*In a preliminary test, we compared the estimated volume densities of secondary lamellae in the gill filaments (V_V(SL/GF)_) of the right and left gills of a healthy rainbow trout (body weight ~1100 g). The estimated values for V_V(SL/GF)_ of the left and right gills (0.278 ± 0.01) (MEAN ± SD) did not differ significantly (p = 0.200, paired t-test).*

**B. Sample number for quantitative morphological analyses of rainbow trout gills.**

*The recommended sample numbers represent orientation values based on previous studies [1] and unpublished preliminary experiments. For example, we compared the values of V_V(SL/GF)_ obtained by analysis of VUR sections of variable numbers of SURS gill filament tissue samples of a rainbow trout of ~1300 g body weight. The V_V(SL/GF)_ values estimated in 18, 15, 12, 9, 6 and 3 SUR samples did not differ significantly (p = 0.845, Kruskal-Wallis-Test), indicating the adequacy of 5 samples per fish for analysis of this parameter with a total coefficient of variance (CV) of 1.7%.*

**C. Examination of gill filament tissue shrinkage during GMA/MMA-embedding.**

*The extent of tissue shrinkage during GMA/MMA-embedding was examined in 12 SUR sampled, cylindrical (2 mm diameter) specimens of formalin-fixed rainbow trout gill filament tissue samples, exactly as described in* ***Fig 14****. The volume of the samples (prior to embedding) was determined from their weight and density (referring to* ***Section 8****). After embedding, the GMA/MMA block was exhaustively serially sectioned with a nominal section thickness of 1.5 µm (1437 sections in total). The factual mean individual section thickness (1.386 ± 0.19 µm) was determined by spectral reflectance measurement (F20, Filmetrics®, USA) [2]. The volume of the GMA/MMA-embedded tissue samples was determined according to the Cavalieri principle from the gill tissue section profile areas measured in every 40th section of the series (36 sections in total).*

**D. Estimation of V_V(SL/GF)_ in VUR sections of SUR sampled gill filament tissue samples.**

*Using the approach described in* ***Section 12****, a mean V_V(SL/GF)_ of 0.286 ± 0.01 (MEAN ± SD) was determined in healthy rainbow trout (n = 2) of ~1100-1300 g body weight.*

**E. Determination of the sufficient number of points hitting section profiles of gill tissue (in all examined fields of view in all sections of all samples per case) to achieve an estimate of V_V(SL/GF)_ with an expected relative error probability of 5% of the mean V_V(SL/GF)_.**

*Using the described procedures, we estimated the volume densities and total volumes of secondary gill lamellae in the gill filaments of a healthy rainbow trout of ~1300 g body weight. 4 SURS samples of the gill filament tissue compartment were taken and processed for generation of VUR GMA/MMA-sections, as described in* ***Sections 9*** *and* ***10****. At 100x microscopic magnification, 46 test field areas were SUR sampled in the VUR sections per case, using a microscopy and stereology system with newCAST™ software (Version 3.6.2.0, Visiopharm, Denmark) and overlaid with a 8x8 point grid. 1338 points were counted hitting section profiles of the GF reference compartment, and 392 points hitting SL section profiles per case. The V_V(SL/GF)_ accounted for 0.293 and the V_(SL,GF)_ for 0.832 cm³. A reduction of the examined test fields (and of the correspondingly counted points) to 15 (with 421 points hitting GF section profiles) did not significantly alter the estimates of V_V(SL/GF)_ (p = 0.976, Kruskal-Wallis-Test), confirming that a total number of ~600 points hitting the reference compartment per case and analysis of a section area sampling fraction of approximately 10% of the total gill tissue section area is sufficient.*

**F. Estimation of S_V(SL/GF)_ and S_(SL,GF)_ in VUR sections of SUR sampled GMA/MMA-embedded gill filament tissue samples.**

*Applying the described method, we estimated S_V(SL/GF)_ in VUR sections of 4 SUR sampled, GMA/MMA-embedded gill filament tissue samples taken from a healthy rainbow trout of ~1300  g body weight. 55 test fields were SUR sampled and photographed at 100x microscopic magnification. The images were enlarged to a final magnification of 300x and digitally overlaid with a stereological test system of 70 points and 35 cycloids (i.e., 2 points per cycloid test arch, cycloid test arch length is calculated from the rectangular frame width as l = 1/10 frame width), aligned to the vertical axis of the VUR gill filament tissue section. 2145 points hitting gill filament tissue section profiles, and 580 intersections between cycloid test lines and the epithelial surface of the secondary lamellae were counted. A (shrinkage-corrected) S_V(SL/GF)_ of
333.53 cm²/cm³ and, correspondingly, a S_(SL,GF)_ of 947.24 cm² was estimated. A 80% reduction of the examined test fields (and of the correspondingly counted points and intercepts) did not significantly alter the estimates of S_V(SL,GF)_ (p = 0.918, Kruskal-Wallis-Test), confirming that a section area sampling fraction of approximately 10% (i.e., a total number of ~15-20 test field areas at 100x microscopic magnification) was sufficient in this example.*

**G. Estimation of N_V(EC/SL)_, V_V(EC/SL)_ and v̅_(EC,SL)_ in IUR sections of SUR sampled Epon-embedded gill filament tissue samples using the physical disector method.**

*In a previous study [1], we used the described disector method for estimation of the number and mean cellular volume of epithelial cells in the secondary gill lamellae of 5 healthy rainbow trout of 269.4 ± 29.0 g body weight. 186 ± 19 Q- were counted on 22 disectors. N_V(EC/SL)_ was estimated as 808.42 ± 87.39 x 1/10^6^ µm^3^, with a mean volume density V_V(EC/SL)_ of 0.514 ± 0.01 and mean cellular SL-EC volume v̅_(EC,SL)_ of 643.60 ± 70.67 µm³.*

**H. Determination of the sufficient number of points hitting section profiles of gill tissue (in all examined fields of view in all sections of all samples per case) to achieve an estimate of V_V(EC/SL)_ with an expected relative error probability of 5% of the mean V_V(EC/SL)_.**

*Using the described procedures, we estimated V_V(EC/SL)_ of a healthy rainbow trout of ~1300 g body weight. 5 SURS samples of the gill filament tissue compartment were taken and processed for generation of VUR GMA/MMA-sections, as described in* ***Sections 9*** *and* ***10****. At 200x microscopic magnification, 15 test field areas (counting frame area of 150 µm x 100 µm) were SUR sampled in the VUR sections per case, using a microscopy and stereology system with newCAST™ software (Version 3.6.2.0, Visiopharm, Denmark) and overlaid with a 8x8 point grid. 287 points were counted hitting section profiles of the SL reference compartment, and 151 points hitting EC section profiles per case. The V_V(EC/SL)_ accounted for 0.526. A reduction of the examined test fields (and of the correspondingly counted points) from 44 (with 885 points hitting SL section profiles) to 15 did not significantly alter the estimates of V_V(EC/SL)_ (p = 0.280, Kruskal-Wallis-Test), confirming that a total number of ~280 points hitting the reference compartment (SL) per case and a grid of 10x10 points per counting frame is sufficient.*

**I. Estimation of T_h(DB)_ in IUR (ultrathin) sections of SUR sampled Epon-embedded gill filament tissue samples (TEM).**

*Using the described method in a previous study [1], we analyzed the true harmonic mean of the diffusion barrier thickness in 5 healthy rainbow trout of 269.4 ± 29.0 g body weight (performing 263 ± 30 measurements in 14.7 ± 0.8 SUR sampled fields of view per case), yielding an average T_h(DB)_ of 3.06 ± 0.12 µm.*

**J. Examination of gill filament tissue shrinkage during 3DISCO clearing.**

*We analyzed the extent of clearing-related gill filament tissue shrinkage by measuring the volumes of 8 individual samples of formalin-fixed gill filament tissue prior to and after 3DISCO clearing. Volumetry was performed using the submersion method described in* ***Section 8****, with 20°C 0.9% saline (ρ = 1.0046 g/cm³) or, respectively, BABB (ρ = 1.0756 ± 0.017 g/cm³) as submersion liquid. On the average, a 3DISCO-associated 3-D volume shrinkage of 50.72 ± 2.88% was determined (referring to a corresponding linear tissue shrinkage factor f_s_ of 0.79 ± 0.01).*

**K. Estimation of V_V(SL/GF)_ and V_(SL,GF)_ as well as (shrinkage-corrected) S_V(SL/GF)_ and S_(SL,GF)_ using SUR sampled virtual optical VUR gill filament tissue sections.**

*V_V(SL/GF)_ was analyzed by LSFM at 100x final magnification, using SUR sampled virtual optical VUR sections acquired in 8 SUR sampled specimens of 3DISCO-cleared gill filament tissue samples, derived from a healthy rainbow trout of ~1300 g body weight. Within the digital images of the virtual optical VUR sections, 63 fields of view were SUR sampled. In these images, V_V(SL/GF)_ was estimated by point counting, using a cross grid of 10x10 points (****Fig 22****). In total, 3030 points hitting the reference compartment were counted. V_V(SL/GF)_ was thus determined as 0.288 and V_(SL,GF)_ as 0.818 cm^3^ for a total gill filament volume of V_(GF)_ = 2.84 cm^3^. A reduction of the examined test fields (and of the correspondingly counted points) to 12 (546 points hitting the digital gill filament tissue section profiles) did not significantly alter the estimates of V_(SL,GF)_ (p = 0.891, Kruskal-Wallis-Test), confirming that a total number of ~600 points hitting the reference compartment per case is sufficient.*

*S_V(SL/GF)_ was analyzed in the same (virtual) optical VUR sections (****Fig 23****) used for estimation of V_V(SL/GF)_. The images were enlarged to a final magnification of 300x (i.e., the true physical factor of magnification of the (printed) image) and overlaid with a test system combining 35 cycloid arches and 70 test points. Per case, 3080 points hitting the gill filament tissue and 951 intersections of the cycloids with the epithelial surface of the secondary gill lamellae were counted. S_V(SL/GF)_ was thus determined as (shrinkage-corrected) 346.24 cm²/cm³, and S_(SL,GF)_ as 983.32 cm^2^. A reduction of the examined VUR section images (and correspondingly counted points) to 8 (and 411 points hitting the digital gill filament tissue section profiles) did not significantly affect the estimates of S_(SL,GF)_ (p = 0.796, Kruskal-Wallis-Test), confirming the robustness of the applied sampling designs and analysis approaches.*

1. Birzle CF. Etablierung und Validierung quantitativ-morphologischer Parameter bei Regenbogenforellen im Rahmen ökotoxikologischer Fragestellungen. Doctoral dissertation, Ludwig-Maximilians-Universität München. 2015.

2. Matenaers C, Popper B, Rieger A, Wanke R, Blutke A. Practicable methods for histological section thickness measurement in quantitative stereological analyses. PLoS One. 2018;13(2): e0192879.
