# Supplementary material for: A practical guide to unbiased quantitative morphological analyses of the gills of rainbow trout (Oncorhynchus mykiss) in ecotoxicological studies
Source: PLoS One. 2020 Dec 9;15(12):e0243462. doi: 10.1371/journal.pone.0243462 (PMC7725368; doi:10.1371/journal.pone.0243462)
Supplement: S1 Eq — (DOCX) [file pone.0243462.s016.docx]

##### S1 Eq. Concentration determination by Lambert-Beer Law.

$\boldsymbol{c=}{\boldsymbol{E}_{\boldsymbol{\lambda}}}/{\boldsymbol{(}\mathcal{E}_{\boldsymbol{\lambda}}\boldsymbol{\times d)}}$

### **c** Concentration of the absorbing substance in the liquid

### **E_λ_** Absorbance of the material for light of wavelength λ

### **Ɛ_λ_** Extinction coefficient at the wavelength λ

### **d** Path length of the cuvette
