# Supplementary material for: A practical guide to unbiased quantitative morphological analyses of the gills of rainbow trout (Oncorhynchus mykiss) in ecotoxicological studies
Source: PLoS One. 2020 Dec 9;15(12):e0243462. doi: 10.1371/journal.pone.0243462 (PMC7725368; doi:10.1371/journal.pone.0243462)
Supplement: S2 Eq — (DOCX) [file pone.0243462.s017.docx]

##### S2 Eq. Determination of dyed liquid volume after dilution.

$\boldsymbol{V}_{\boldsymbol{A}}\boldsymbol{=}\boldsymbol{V}_{\boldsymbol{P}}\boldsymbol{\times}{\boldsymbol{c}_{\boldsymbol{P}}}/{\boldsymbol{c}_{\boldsymbol{A}}}$

### **V_A_** Volume of the dyed liquid volume after submersion (*i.e.,* diluted by the liquid attached to the wet gill filament sample)

### **V_P_** Volume of the dyed liquid prior to submersion of the (wet) gill filament sample

### **c_P_** Concentration of the dyed submersion liquid prior to submersion of the (wet) gill filament sample

**c_A_** Concentration of the dyed submersion liquid after submersion of the (wet) gill filament sample (*i.e.,* diluted)
